# Supplementary material for: Lifestyle and demographic associations with 47 inflammatory and vascular stress biomarkers in 9876 blood donors
Source: Commun Med (Lond). 2024 Mar 16;4:50. doi: 10.1038/s43856-024-00474-2 (PMC10944541; doi:10.1038/s43856-024-00474-2)
Supplement: Supplementary file 2 — Supplementary Information [file 43856_2024_474_MOESM2_ESM.pdf]

Sex, age, BMI, and smoking associations with 47 inflammatory and vascular stress biomarkers in 9,876 blood donors

Bertram Kjerulff[1-3*], Joseph Dowsett[4], Rikke Louise Jacobsen[4], Josephine Gladov[1-3], Margit Hørup Larsen[4], Agnete Troen Lundgaard[5], Karina Banasik[5], David Westergaard[5,6], Susan Mikkelsen[1], Khoa Manh Dinh[1], Lotte Hindhede[1], Kathrine Agergård Kaspersen[1,3], Michael Schwinn[4], Anders Juul[6,7,8], Betina Poulsen[4], Birgitte Lindegaard[6,9], Carsten Bøcker Pedersen[3,10], Clive Eric Sabel[3,11,], Henning Bundgaard[6,12], Henriette Svarre Nielsen[6,13,14], Janne Amstrup Møller[4], Jens Kjærgaard Boldsen[1,3], Kristoffer Sølvsten Burgdorf[5], Lars Vedel Kessing[6,15], Linda Jenny Handgaard[4], Lise Wegner Thørner[4], Maria Didriksen[4], Mette Nyegaard[16], Niels Grarup[17], Niels Ødum[18], Pär I Johansson[4,14], Poul Jennum[6,19], Ruth Frikke-Schmidt[6,20], Sanne Schou Berger[21], Søren Brunak[5], Søren Jacobsen[6], Thomas Folkmann Hansen[5,23], Tine Kirkeskov Lundquist[4], Torben Hansen[17], Torben Lykke Sørensen[6,24], Torben Sigsgaard[3,11], Kaspar René Nielsen[25], Mie Topholm Bruun[26], Henrik Hjalgrim[6,27-29], Henrik Ullum[30], Klaus Rostgaard[27,28], Erik Sørensen[4], Ole Birger Pedersen[6,31#], Sisse Rye Ostrowski[4,6#], Christian Erikstrup[1-3#]

Table of contents

Item	Page
Quality control	
Supplementary Table 1 – Kit LOT number	1
Supplementary Figure 1 – internal control plot	2
Supplementary Figure 2 – Regional differences	3
Supplementary Figure 3 – Storage time	4
Supplementary Figure 4 – Time of day	5
Supplementary Figure 5 – Time of day	6
Supplementary Figure 6 – Density plots	10
Results	
Supplementary Table 2 – Age of smokers	14
Supplementary Figure 7 – Directed acyclic graphs (DAGs)	15
Supplementary Figure 8 – CRP and age	16
Supplementary Table 3–49 – Details for each biomarker	17

Supplementary Table 1

Kit LOT numbers used.

**Panel (all human)**	**LOT numbers**
Proinflammatory Panel 1	K0081327, K0081744
Cytokine Panel 1	K0081333, K0081702
Cytokine Panel 2	K0081180, K0081550,
TH17 Panel 1 (excluded)	K0081212
Angiogenesis Panel 1	K0081316, K0081690
Chemokine Panel 1	K0081263, K0081682
Vascular Injury Panel 2	K0081395, K0081715

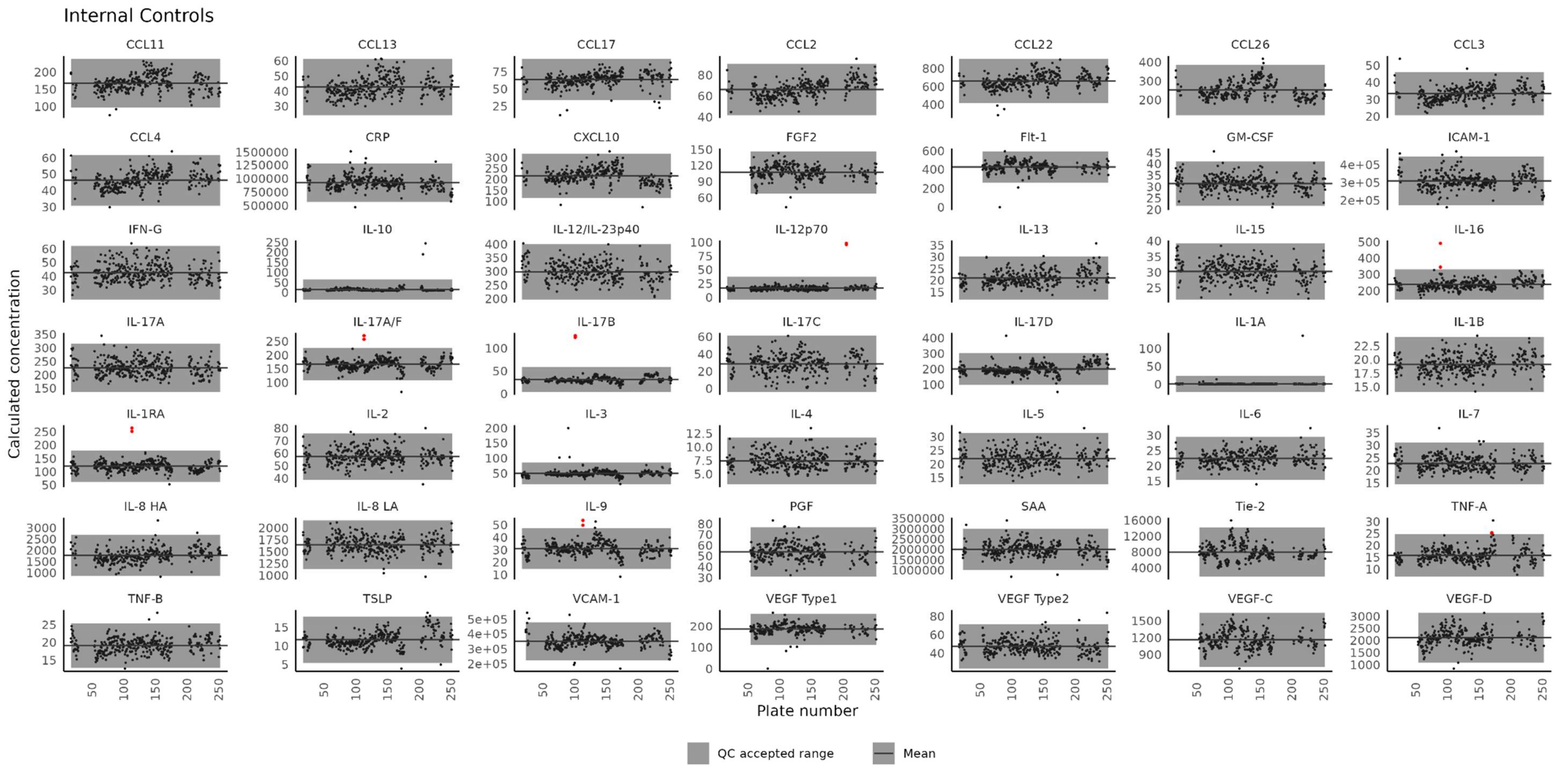

Supplementary Figure 1: Plot of internal controls. Samples are excluded (red) if both internal controls fall outside the QC accepted range, which is above or below 3 standard deviations from the mean.

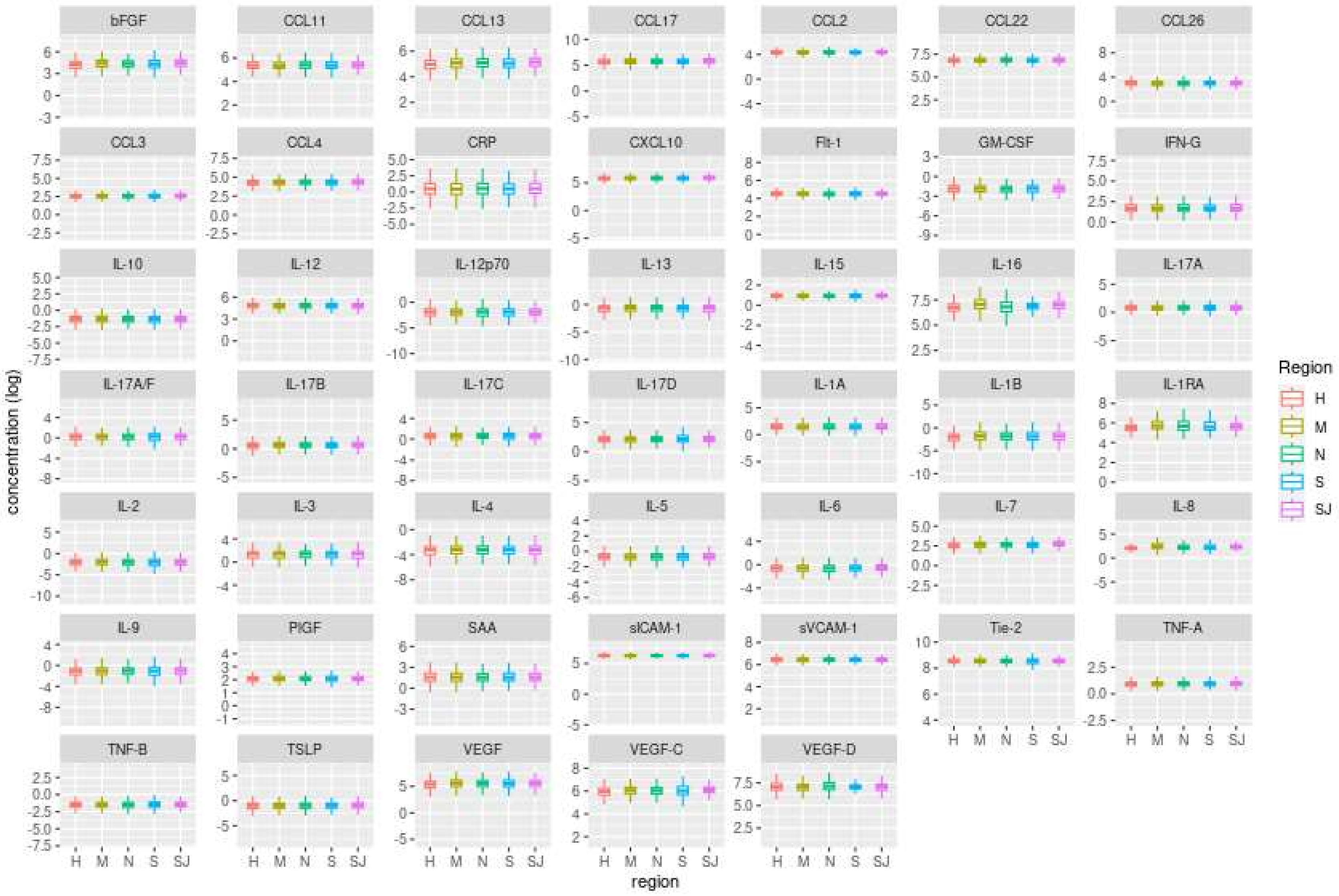

Supplementary Figure 2: Boxplot of regional differences. H is Capital Region, M is Central Denmark Region, N is North Denmark, S is South Denmark, and Sj is Zealand. Boxes displaying median and interquartile ranges.

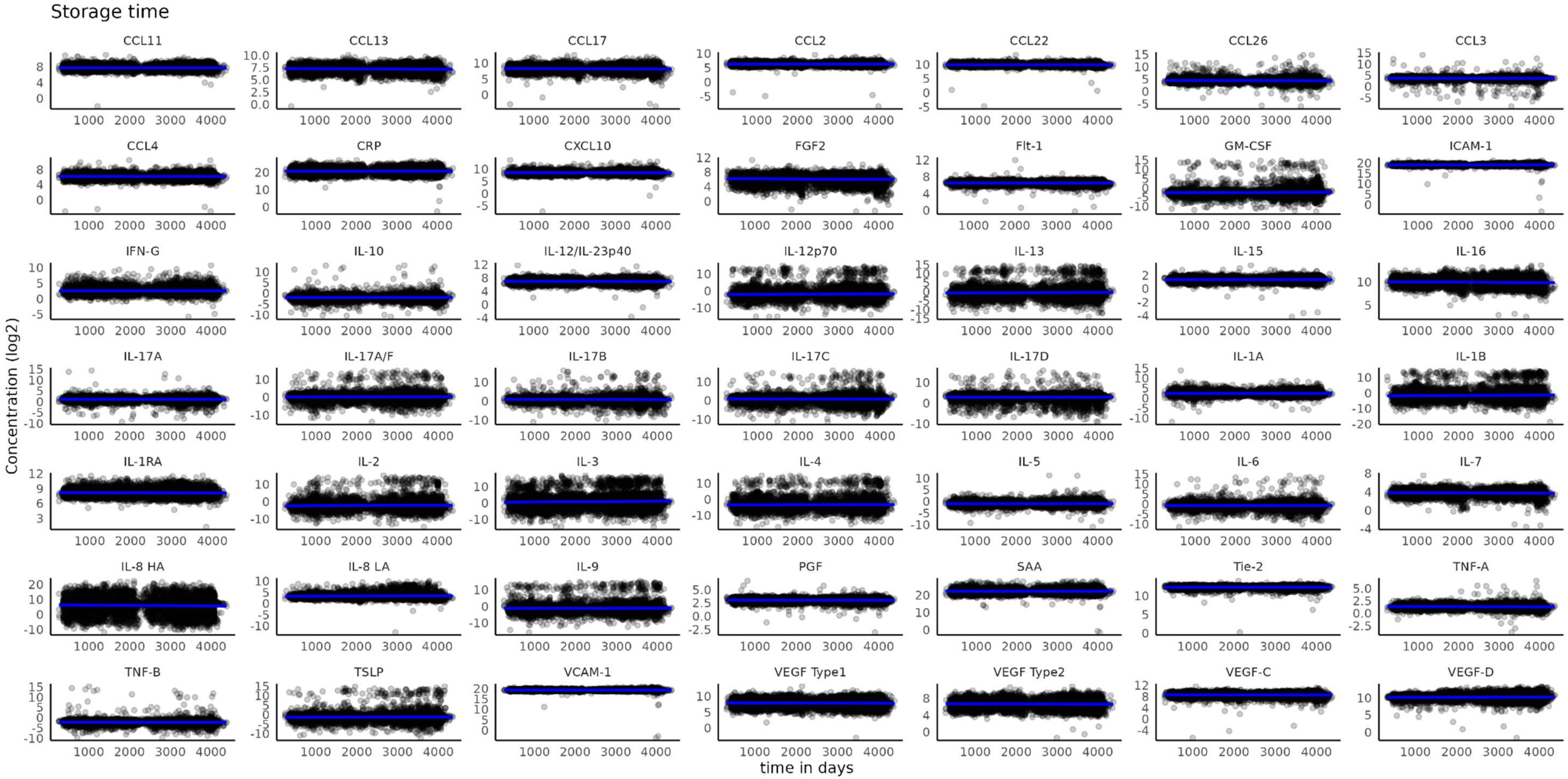

Supplementary Figure 3: The effect of storage time in days for each biomarker.

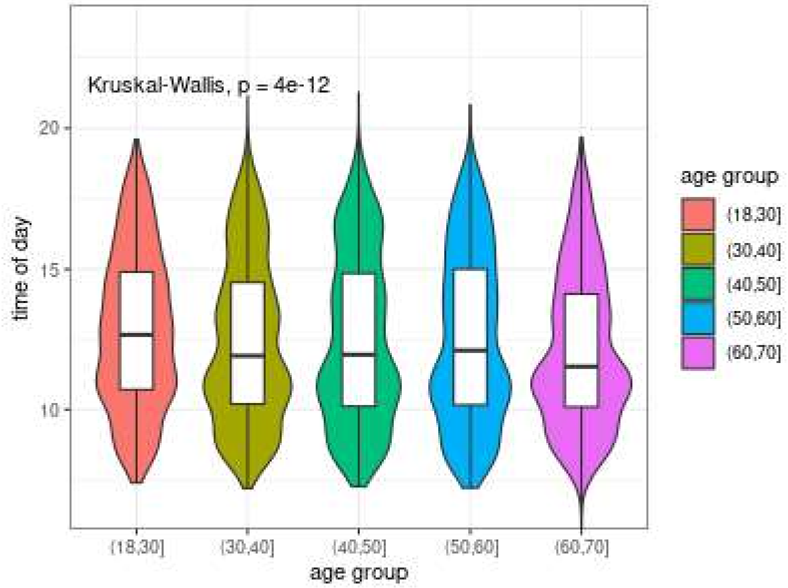

Supplementary Figure 4: *Decreasing median time of day of donations with age. Violin plot with overlaid box plot of the time of day for donations in each age group. Y-axis is hour of the day from 5 am to 10 PM. Boxes displaying median and interquartile ranges. Vertical lines from min to max. n=9,563 participants.*

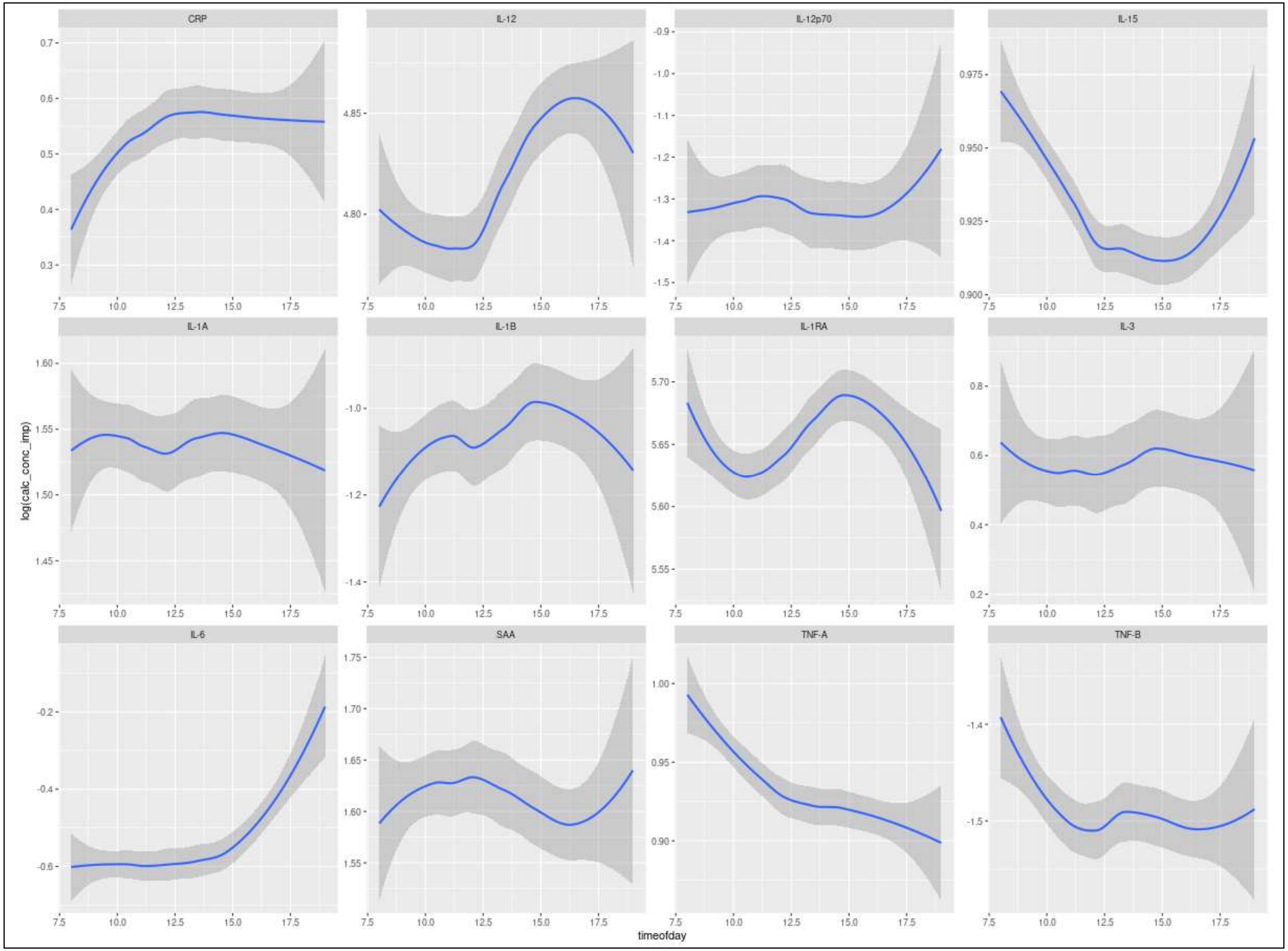

CRP
IL-12
IL-12p70
IL-15
IL-1A
IL-1B
IL-1RA
IL-3
IL-6
SAA
TNF-A
TNF-B
log(calc_conc_imp)
timeofday

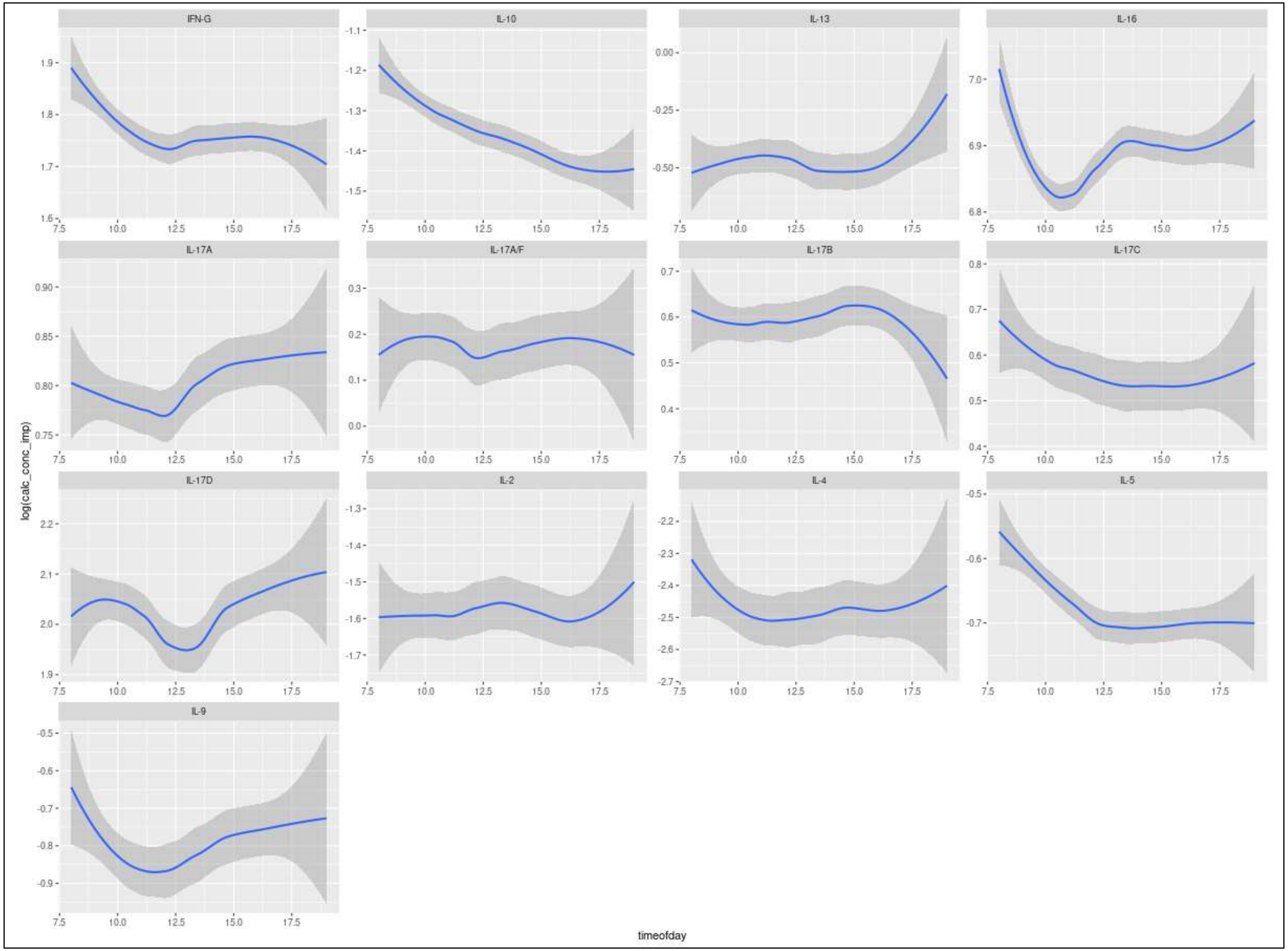

IFN-G
IL-10
IL-13
IL-16
IL-17A
IL-17A/F
IL-17B
IL-17C
IL-17D
IL-2
IL-4
IL-5
IL-9
log(calc_conc_imp)
timeofday

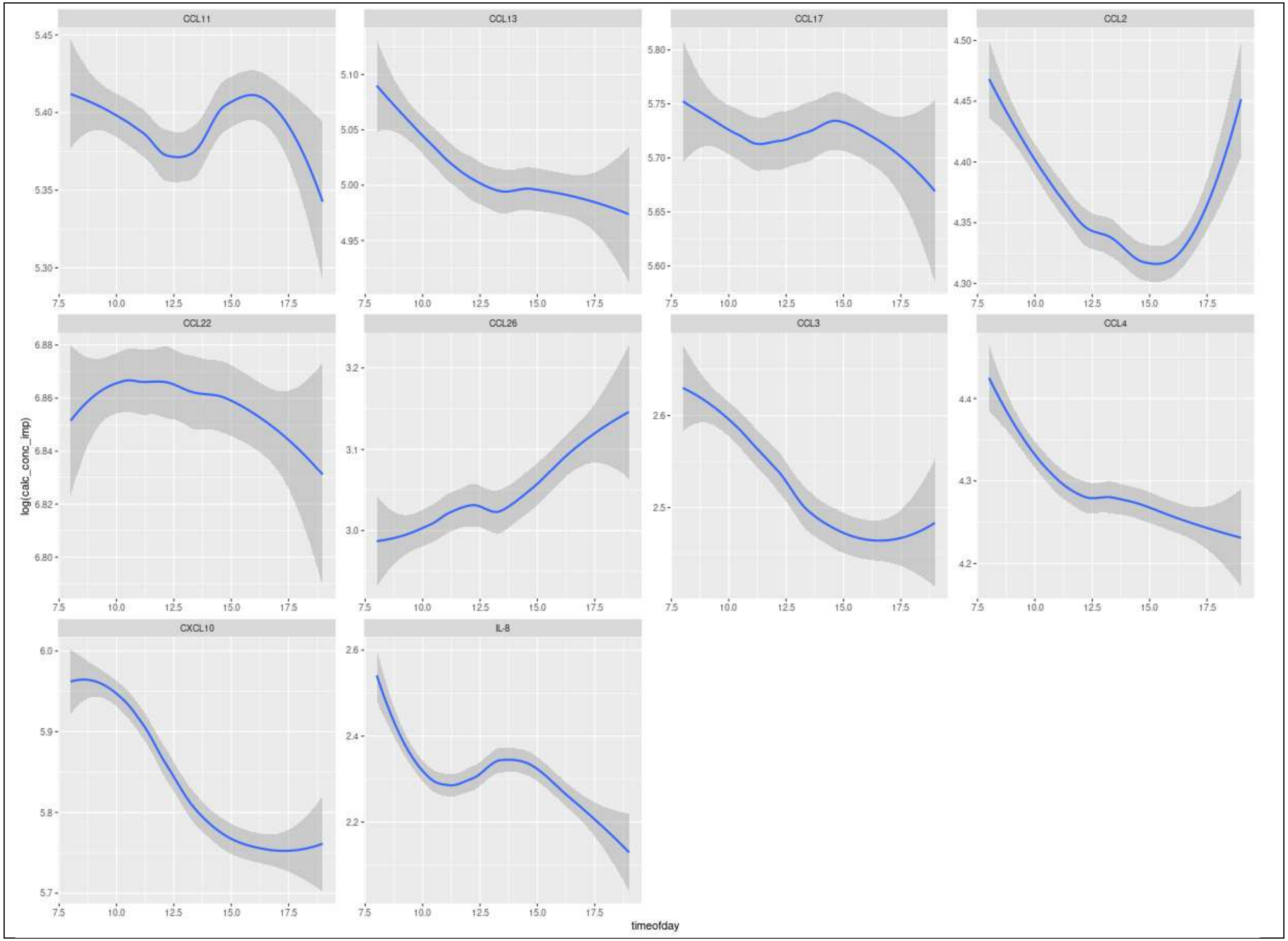

CCL11
CCL13
CCL17
CCL2
CCL22
CCL26
CCL3
CCL4
CXCL10
IL-8
log(calc_conc_imp)
timeofday

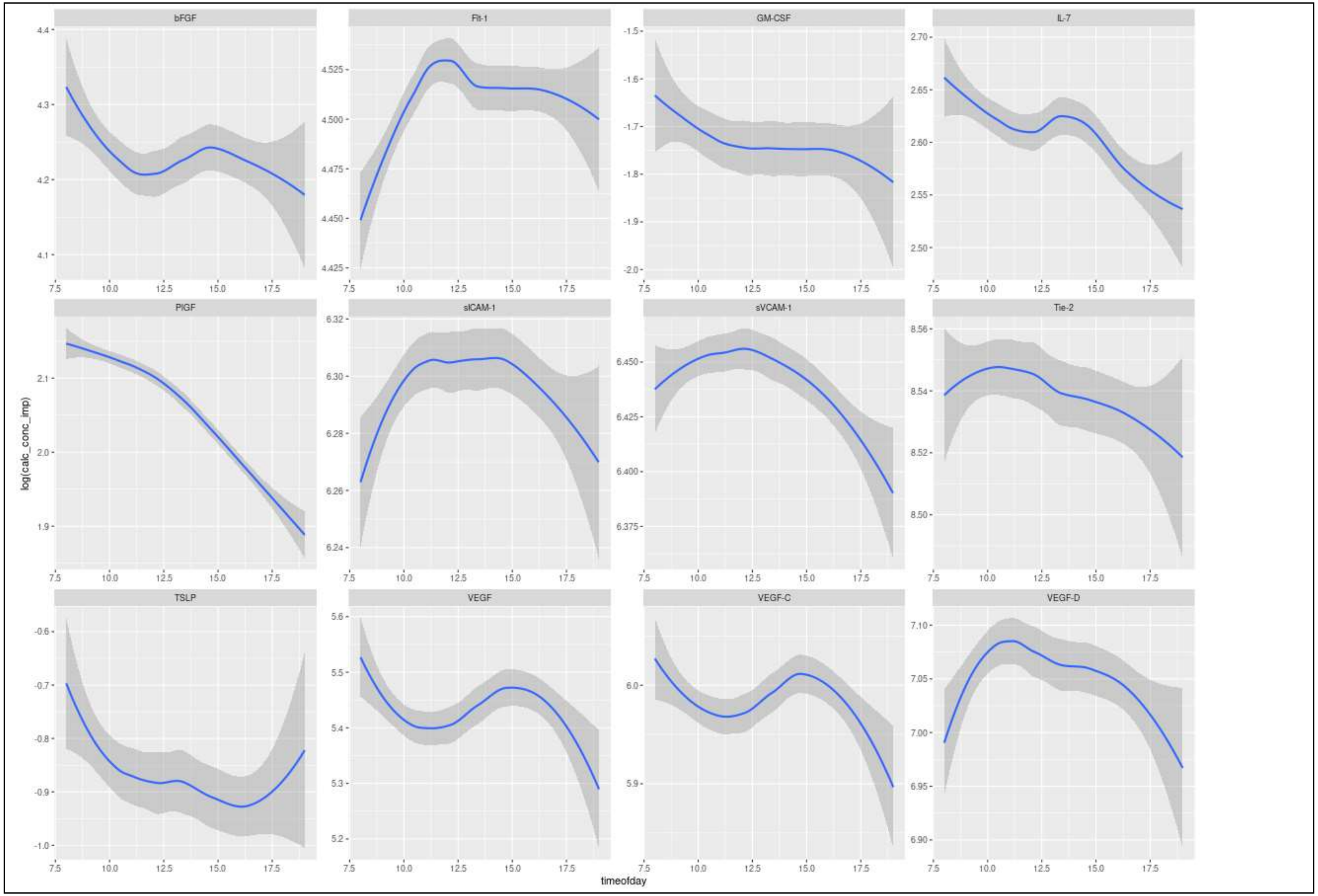

Supplementary Figure 5: Log(concentration) and time of day for each assay. Plotted with smoothed conditional means and local polynomial regression fitting with x-axis limits set to the hours 8–19.

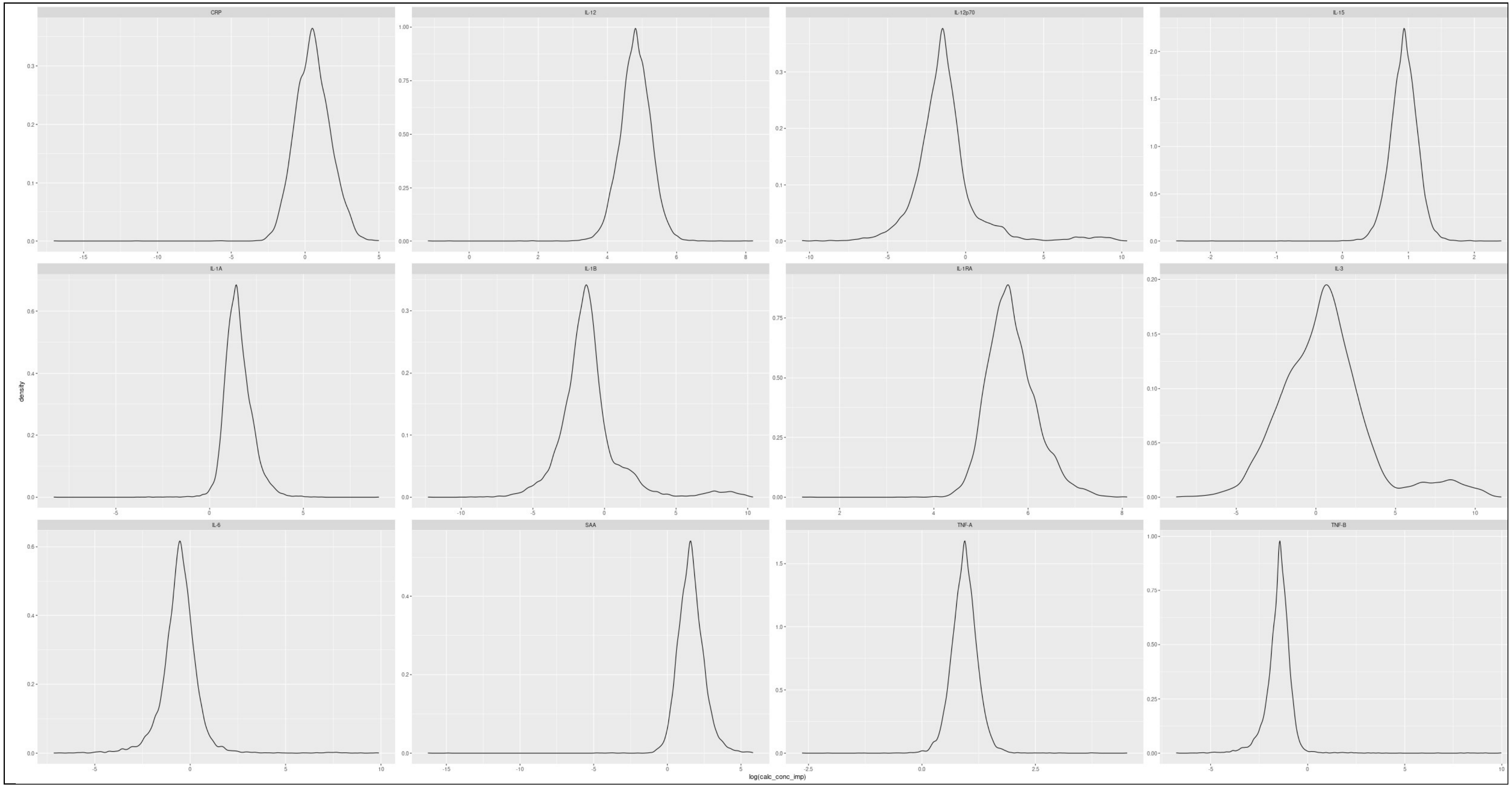

CRP
IL-12
IL-12p70
IL-15
IL-1A
IL-1B
IL-1RA
IL-3
IL-6
SAA
TNF-A
TNF-B
density
log(calc_conc_imp)

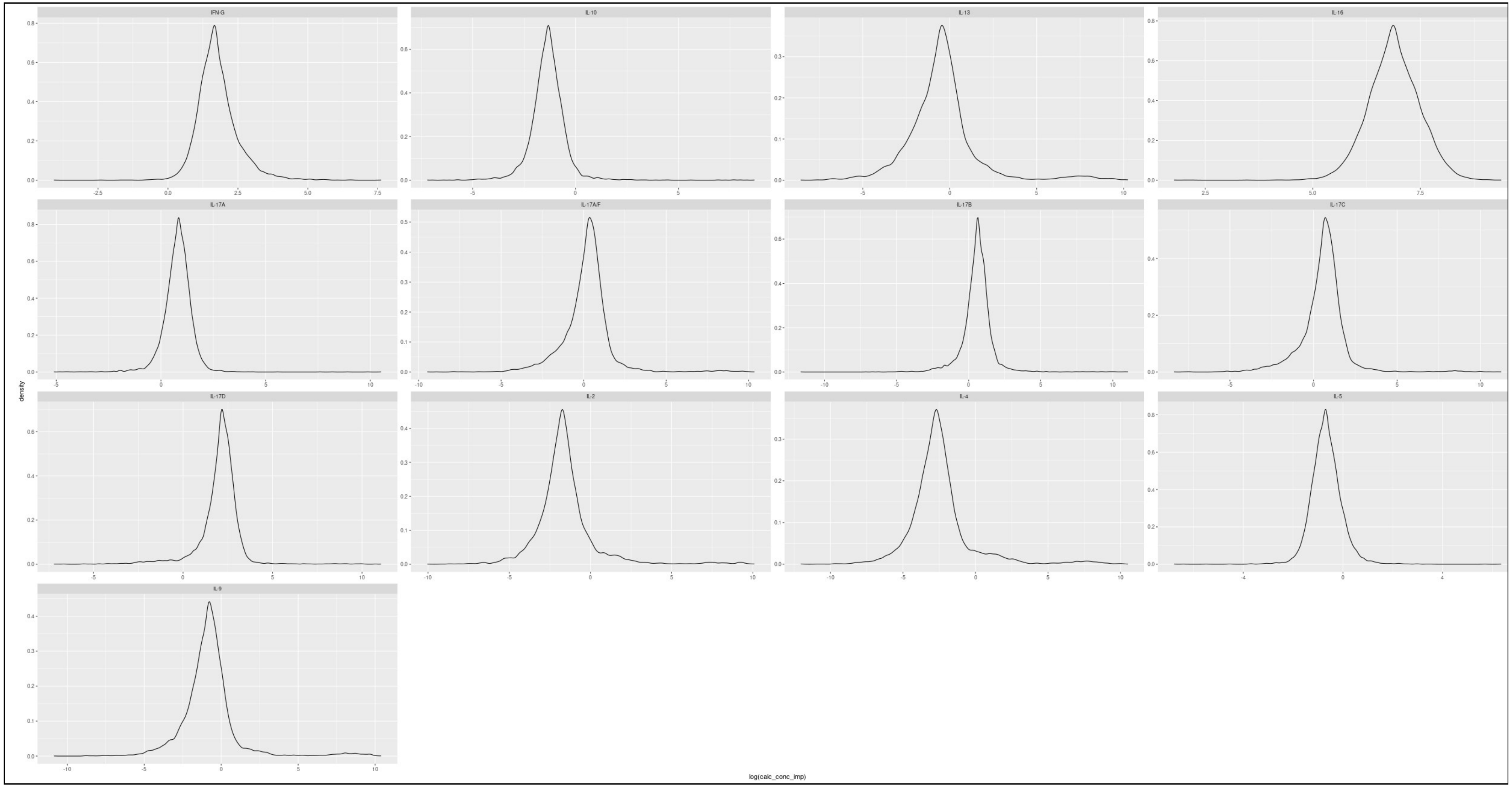
IFN-G
IL-10
IL-13
IL-16
IL-17A
IL-17A/F
IL-17B
IL-17C
IL-17D
IL-2
IL-4
IL-5
IL-9
density
log(calc_conc_imp)

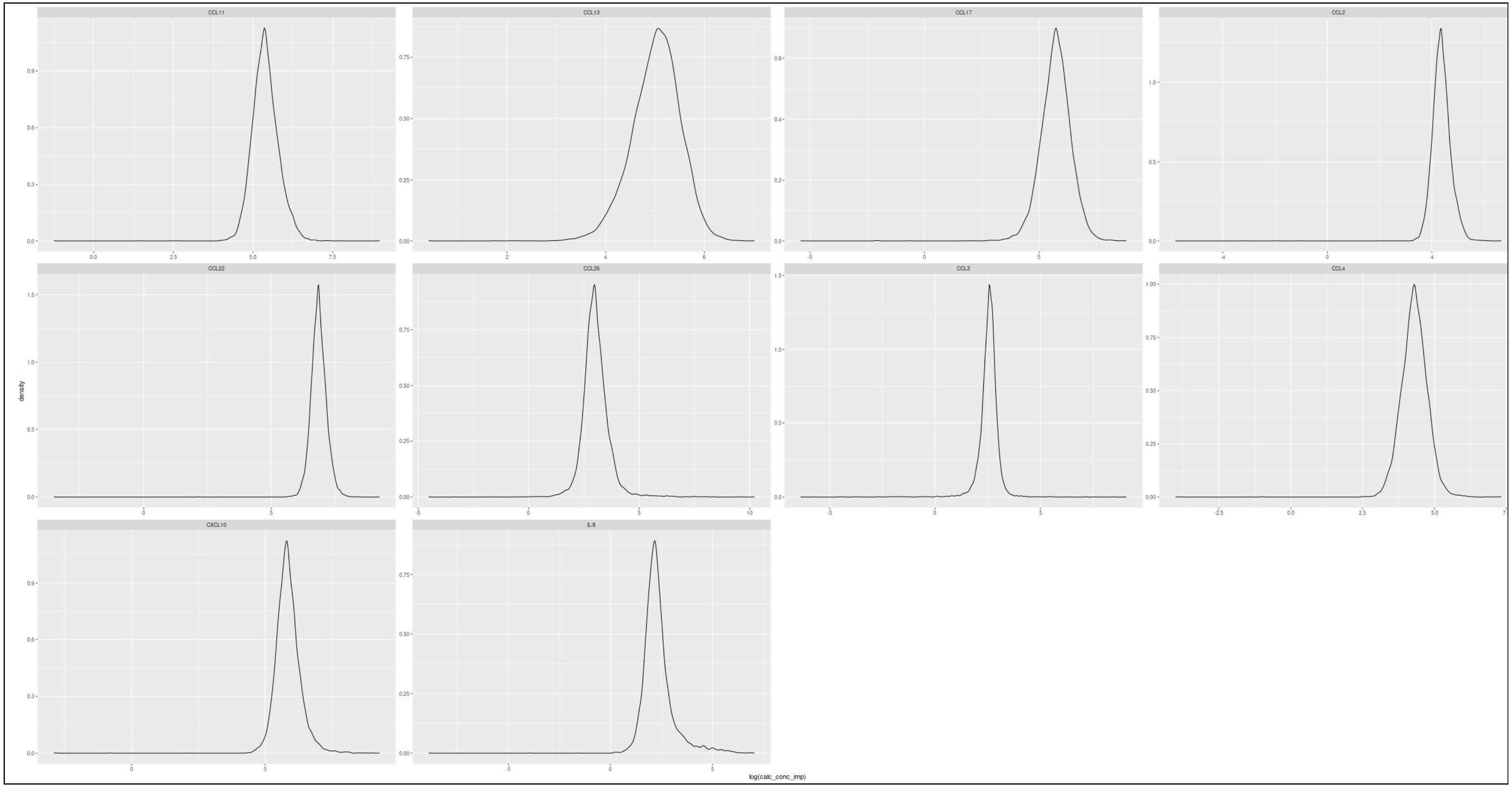
CCL11
CCL13
CCL17
CCL2
CCL22
CCL26
CCL3
CCL4
CXCL10
IL-8
density
log(calc_conc_imp)

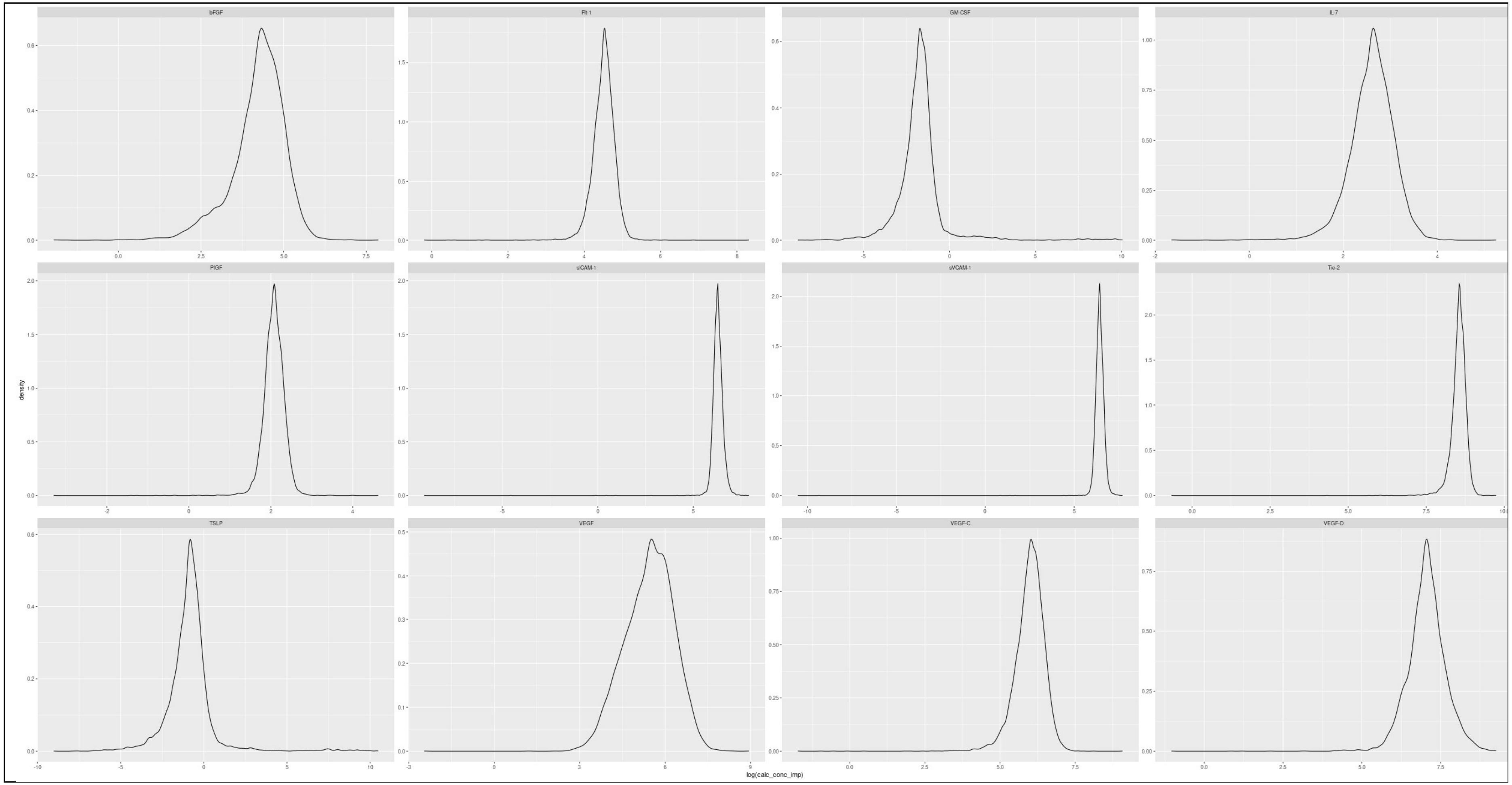

Supplementary Figure 6: Density plots of log(concentrations) for all assays after median normalization.

Supplementary Results

Supplementary Table 2

Number and proportion of smokers in each age group.

**Age group**	**smokers**	**Non-smokers**	**%**
18–30	304	1,584	16.1%
30–40	251	1,660	13.1%
40–50	245	1,663	12.8%
50–60	249	1,649	13.1%
60+	222	1,536	12.6%

1. Association between sex, age and biomarkers:

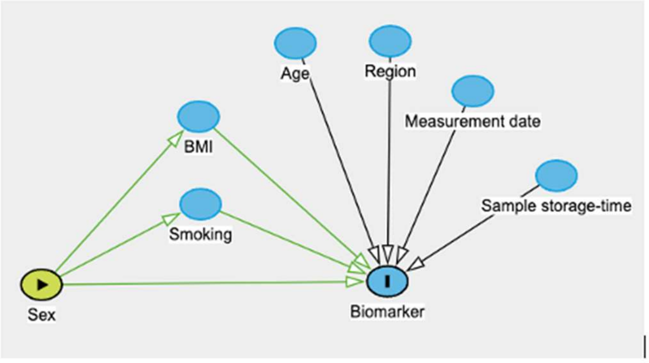

2. Association between BMI and biomarkers:

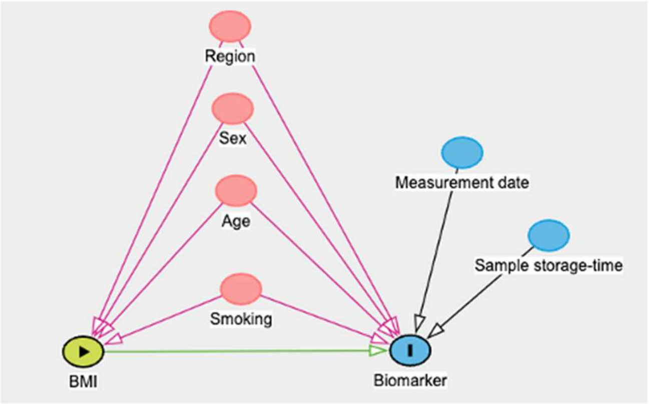

3. Association between smoking and biomarkers:

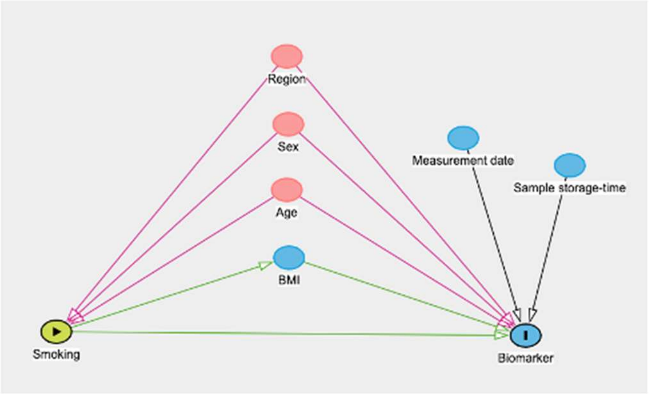

Supplementary Figure 7: *Directed acyclic graphs (DAGs) for each of the three models illustrating the causal structure of the chosen variables. BMI, body mass index.*

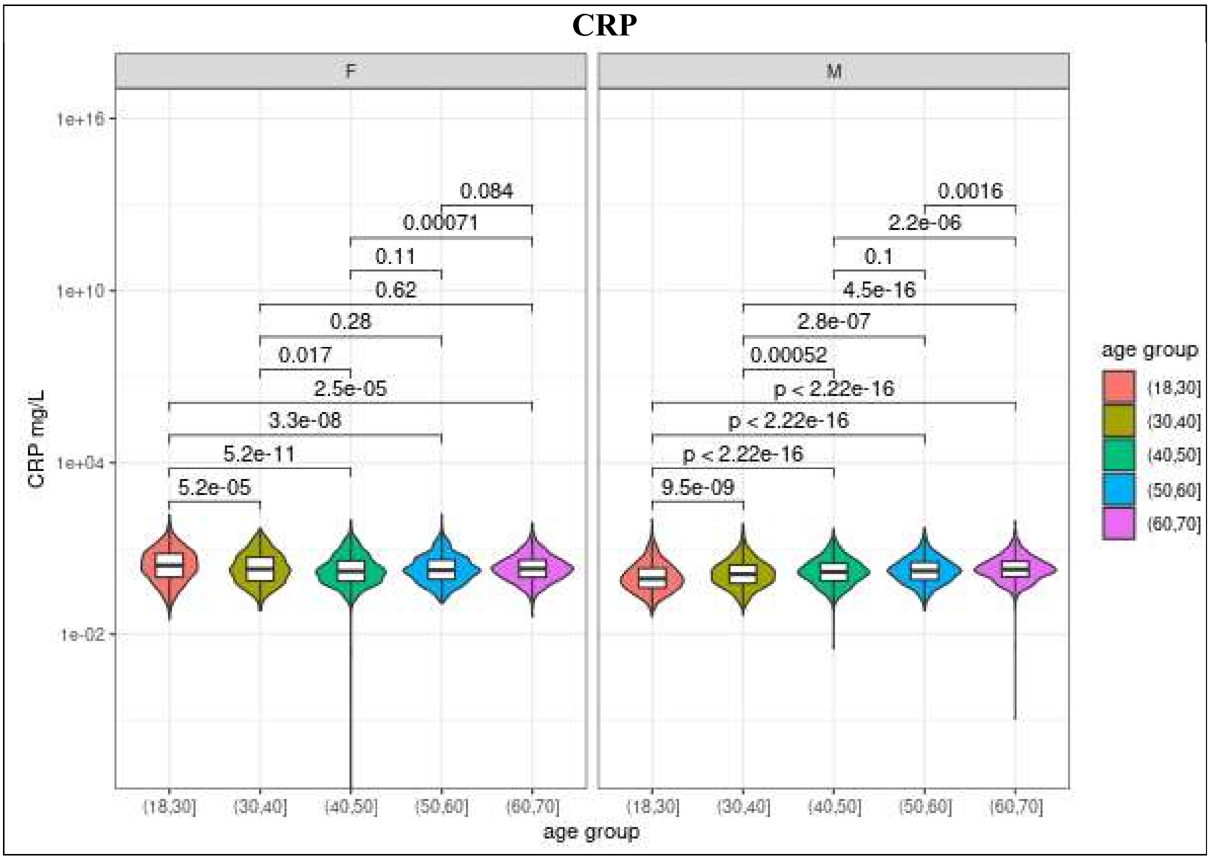

Supplementary Figure 8: *Violin plots with overlaid boxplots of the log10(concentration) of CRP for males (M) and females (F) in the five age groups. Test by Wilcoxon ranksum. N=9,503 participants. Boxes displaying median and interquartile ranges. Vertical lines from min to max.*

Overview of the influence of sex, age, and BMI on the individual biomarkers.

This section provides an overview per biomarker level. Biomarkers are sorted alphabetically within groups

Description

Concentrations are provided as medians of normalized concentration with IQR in all individuals and in each stratified group.
Proportions are results from linear regressions adjusted for sex, age, smoking, BMI, region, sample storage time, and measurement date as relevant. Proportions are displayed as percentage change estimate and [95% confidence interval]. If the coefficient is statistically significant after Bonferroni correction, the value is in **bold**. Percentage change in the BMI groups shown in **bold** are the ones for which the BMI group was statistically significantly higher in regression model compared to normal after Bonferroni correction.

Detection range shows the % of samples in the detection range.
Concentrations for males and females shows the median and interquartile range of the concentration for the given stratification.
Sex difference shows the estimated % difference in concentration in females compared to males. If coefficient is statistically significant after Bonferroni correction the value is in **bold**.
Sex*age shows the significance of the interaction term (the difference in change with a 10-year age increase).
BMI group*sex shows the significance of the interaction term between BMI group and sex. That is the significance of the difference in effect of going from normal BMI to overweight or obese between sexes.
BMI group concentrations show the median concentration for the BMI group stratified by age group and sex.
Percentage change in the BMI analysis shows the estimated % change in concentrations from regression model for the BMI group compared to normal BMI group.
Smoking*sex shows the significance of the interaction term between smoking and sex. That is the significance of the difference in effect of smoking between sexes.

As stated in methods in the main text, the Bonferroni correction was performed within each of the biomarker groupings. The significance levels were 0.00416 for proinflammatory and growth factor biomarkers, 0.00385 for T cell derived, and 0.005 for chemokines.

Contents

Table S3: CRP (C reactive protein, PTX1, Uniprot:P02741)........ 19
Table S4: IL-1α (Interleukin 1 alpha, IL1A, IL1F1, Uniprot:P01583) 19
Table S5: IL-1β (Interleukin 1 beta, IL1B, IL1F2, Uniprot:P01584)........ 20
Table S6: IL-1RA (Interleukin-1 receptor antagonist, IL1RN, DIRA, Uniprot:P18510).... 21
Table S7: IL-3 (Interleukin-3, MCGF, MULTI-CSF, Uniprot:P08700) 22
Table S8: IL-6 (Interleukin-6, BSF2, HGF, HSF, IFNB2, Uniprot:P05231) 23
Table S9: IL-12/IL-23p40 (Interleukin-12 subunit beta, CLMF, IL12B, NKSF, Uniprot:P29460) 24
Table S10: IL-12p70 (Interleukin-12, Uniprot:P29459) 25
Table S11: IL-15 (Interleukin-15, Uniprot:P40933) 26
Table S12: SAA (Serum amyloid A, Uniprot:P0DJI8/9)........ 27
Table S13: TNFα (Tumor Necrosis Factor-alpha, TNF, DIF, TNFSF2, TNLG1F, Uniprot:P01375) 28
Table S14: TNFβ (Tumor Necrosis Factor-beta, Lymphotoxin alpha, LTA, TNFSF1, TNLG1E, Uniprot:P01374) 29
Table S15: IFNγ (Interferon gamma, IFNG, IFG, IFI, Uniprot:P01579)........ 31
Table S16: IL-2 (Interleukin-2, TCGF, Uniprot:P60568)........ 31
Table S17: IL-4 (Interleukin-4, 4BSF, Uniprot:P05112)........ 32
Table S18: IL-5 (Interleukin-5, Uniprot:P05113)........ 33
Table S19: IL-9 (Interleukin-9, HP40, Uniprot:P15248)........ 35
Table S20: IL-10 (Interleukin-10, CSIF, GVHDS, Uniprot:P22301) 36
Table S21: IL-13 (Interleukin-13, P600, Uniprot:P35225)........ 37
Table S22: IL-16 (Interleukin-16, LCF, NPRprIL-16, Uniprot:Q14005) 38
Table S23: IL-17A (Interleukin-17A, CTLA8, Uniprot:Q16552)........ 39
Table S24: IL-17A/F (IL17F, Interleukin-17A and F heterodimer, Uniprot:Q96PD4)....... 40
Table S25: IL-17B (Interleukin-17B, Uniprot:Q9UHF5) 41
Table S26: IL-17C (Interleukin-17C, Uniprot:Q9P0M4) 42
Table S27: IL-17D (Interleukin-17D, Uniprot:Q8TAD2)........ 43
Table S28: CCL2 (monocyte chemoattractant protein 1, MCP1, GDCF-2, HC11, Uniprot:P13500) 44
Table S29: CCL3 (macrophage inflammatory protein 1α, SCYA3, MIP1α, Uniprot:P10147) 45
Table S30: CCL4 (macrophage inflammatory protein 1β, MIP1β, SCYA4, LAG1, Uniprot:P13236) 46
Table S31: CCL11 (Eotaxin, Uniprot:P51671)........ 47
Table S32: CCL13 (monocyte chemoattractant protein 4, MCP4, Uniprot:Q99616) 48
Table S33: CCL17 (thymus- and activation-regulated chemokine, TARC, ABCD-2, SCYA17, Uniprot:Q92583) 49
Table S34: CCL22 (macrophage derived chemokine, MDC, ABCD-1, Uniprot:O00626). 50
Table S35: CCL26 (Eotaxin-3, MIP-4α, IMAC, Uniprot:Q9Y258)........ 51
Table S36: CXCL10 (Interferon gamma-induced protein 10, IP10, IFI10, SCYB10, Uniprot:P02778) 52
Table S37: IL-8 (Interleukin-8, CXCL8, GCP-1, LECT, LUCT, LYNAP, MDNCF, Uniprot:P10145) 53
Table S38: bFGF (Basic Fibroblast growth factor, FGF2, HBGF-2, Uniprot:P09038)....... 54
Table S39: Flt1 (Vascular Endothelial Growth Factor Receptor 1, Uniprot:P17948)........ 55
Table S40: GM-CSF (Granulocyte-macrophage colony-stimulating factor, CSF2, Uniprot:P04141) 56
Table S41: IL-7 (Interleukin-7, Uniprot:P13232) 57
Table S42: PlGF (Placental growth factor, PGF, Uniprot:P49763)........ 58
Table S43: sICAM1 (soluble intracellular adhesion molecule 1, CD54, BB2, Uniprot:Q99930/P05362)........ 59
Table S44: sVCAM1 (soluble Vascular cell adhesion protein 1, CD106, INCAM-100, Uniprot:P19320) 60
Table S45: TIE2 (TEK tyrosine kinase, CD202B, CMCM, Uniprot:Q02763) 61
Table S46: TSLP (thymic stromal lymphopoietin, Uniprot:Q969D9) 62
Table S47: VEGF-A (Vascular endothelial growth factor A, MVCD1, VPF, Uniprot:P15692) 63
Table S48 VEGF-C (Vascular endothelial growth factor C, Flt-4L, LMPH1D, VRP, Uniprot:P49767) 64
Table S49: VEGF-D (Vascular endothelial growth factor D, c-fos induced growth factor, Uniprot:O43915)........ 65

Supplementary Table 3: CRP (C reactive protein, PTX1, Uniprot:P02741)

Sex and age-stratified median concentrations

AGE GROUP	Biomarker/cohort information		Median Concentration			Age Group (% change in concentration)		
	**N measured**	**Detection Range**	**Males**	**Females**	**Sex difference**	**Males**	**Females**	**Sex × age**
		(% in range)	(mg/l)	(mg/l)	F relative to M (%)	change/10 years (%)	change/10 years (%)	Interaction P
**All**	9503	100	1.4 (0.68–2.9)	1.9 (0.85–4.6)	**237[192 : 290]**	**12[9.1 : 14]**	**-7.2[-9.3 : -5.1]**	**9.08E-31**
**18–29**	1879	100	0.89 (0.43–2.0)	2.5 (1.0–6.5)	245[44 : 725]	-5.1[-26 : 22]	-16[-35 : 7.7]	0.488
**30–39**	1920	100	1.3 (0.61–2.6)	1.9 (0.74–4.9)	207[-5.6 : 896]	-4.3[-25 : 21]	-20[-37 : 1.1]	0.288
**40–49**	1939	99.9	1.5 (0.75–3.0)	1.6 (0.73–3.7)	145[-43 : 959]	15[-8.1 : 44]	-1.8[-22 : 24]	0.336
**50–59**	1928	100	1.6 (0.82–3.3)	1.7 (0.85–4.0)	-14[-84 : 367]	2.2[-18 : 27]	9.7[-12 : 37]	0.655
**60–69**	1837	99.9	1.9 (1.0–3.6)	2.0 (1.0–3.9)	-16[-96 : 1638]	2.1[-27 : 42]	7.4[-24 : 52]	0.835

BMI and age-stratified median concentrations

AGE GROUP	Normal BMI		Overweight BMI					Obese BMI				
	**Males**	**Females**	**Males**	**Females**	**Males**	**Females**	**Overweight × sex**	**Males**	**Females**	**Males**	**Females**	**Obese × sex**
	Concentration (mg/l)	Concentration (mg/l)	Concentration (mg/l)	Concentration (mg/l)	%change	%change	Interaction P	Concentration (mg/l)	Concentration (mg/l)	%change	%change	Interaction P
**All**	0.96 (0.46–1.9)	1.3 (0.61–2.7)	1.7 (0.87–3.2)	2.4 (1.3–5.1)	**76[64 : 88]**	**19[8.5 : 30]**	0.098	3.1 (1.8–5.6)	5.9 (2.6–12)	**235[203 : 270]**	**30[11 : 53]**	**0.003**
**18–29**	0.74 (0.36–1.6)	2.0 (0.76–4.8)	1.2 (0.64–2.6)	3.8 (1.6–8.7)	**69[41 : 103]**	3.0[-19 : 32]	0.572	2.4 (1.4–5.4)	8.4 (3.9–15)	**243[152 : 367]**	9.0[-31 : 71]	0.397
**30–39**	0.81 (0.42–1.7)	1.2 (0.55–2.8)	1.4 (0.74–2.7)	2.3 (1.2–5.2)	**69[45 : 97]**	28[5.1 : 56]	0.122	3.0 (1.7–6.4)	6.3 (3.5–14)	**291[217 : 382]**	58[11 : 127]	0.044
**40–49**	0.96 (0.46–1.8)	0.97 (0.55–2.1)	1.7 (0.96–3.4)	1.8 (1.1–3.7)	**83[59 : 112]**	19[-1.6 : 44]	0.741	3.0 (1.8–5.1)	5.5 (2.0–11)	**236[175 : 309]**	38[-0.83 : 91]	0.043
**50–59**	1.1 (0.55–2.3)	1.1 (0.59–2.1)	1.7 (0.96–3.2)	2.4 (1.4–4.7)	**61[41 : 85]**	30[8.5 : 57]	0.005	3.1 (2.0–6.3)	5.7 (2.5–12)	**213[157 : 282]**	43[1.1 : 102]	0.011
**60–69**	1.4 (0.76–2.5)	1.3 (0.76–2.3)	2.0 (1.2–3.9)	2.6 (1.5–4.8)	**48[28 : 72]**	**38[14 : 67]**	0.002	3.3 (1.8–5.8)	5.2 (2.6–10.)	**143[94 : 205]**	48[3.8 : 110]	0.006

Smoking and age-stratified median concentrations

AGE GROUP	Non-smokers		Smokers		% Change in Concentration smokers vs non-smokers		
	**Males**	**Females**	**Males**	**Females**	**Males**	**Females**	**Smoking × sex**
	Concentration (mg/l)	Concentration (mg/l)	Concentration (mg/l)	Concentration (mg/l)	change (%)	change (%)	Interaction P
**All**	1.4 (0.68–2.8)	1.8 (0.84–4.4)	1.7 (0.77–3.7)	2.2 (0.82–5.1)	**23[12 : 34]**	5.4[-3.6 : 15]	0.02
**18–29**	0.89 (0.44–2.1)	2.5 (0.97–6.5)	1.0 (0.46–2.0)	2.5 (1.0–6.4)	1.3[-18 : 26]	-15[-31 : 4.7]	0.248
**30–39**	1.3 (0.60–2.6)	1.8 (0.74–4.8)	1.5 (0.71–3.4)	2.4 (0.71–6.6)	17[-5.0 : 44]	8.0[-12 : 32]	0.591
**40–49**	1.5 (0.74–2.9)	1.5 (0.72–3.4)	2.2 (0.92–3.8)	1.6 (0.73–4.1)	**28[4.2 : 56]**	7.1[-12 : 30]	0.221
**50–59**	1.6 (0.80–3.1)	1.7 (0.85–3.8)	2.3 (1.0–4.8)	2.2 (0.68–4.9)	**40[17 : 69]**	8.2[-10 : 31]	0.052
**60–69**	1.8 (0.97–3.3)	2.0 (1.0–4.0)	2.5 (1.3–6.2)	1.9 (0.91–3.6)	**40[14 : 72]**	9.0[-10 : 32]	0.079

Supplementary Table 4: IL-1α (Interleukin 1 alpha, IL1A, IL1F1, Uniprot:P01583)

Sex and age-stratified median concentrations

AGE GROUP	Biomarker/cohort information		Median Concentration			Age Group (% change in concentration)		
	**N measured**	**Detection Range** (% in range)	**Males** (pg/ml)	**Females** (pg/ml)	**Sex difference** F relative to M (%)	**Males** change/10 years (%)	**Females** change/10 years (%)	**Sex × age** Interaction P
**All**	9872	99.8	4.4 (3.0–7.3)	4.2 (2.9–6.5)	-5.4[-15 : 4.7]	1.0[-0.55 : 2.6]	0.30[-1.2 : 1.9]	0.517
**18–29**	1994	99.9	4.3 (3.0–7.2)	4.1 (2.8–6.5)	44[-16 : 149]	**29[9.7 : 51]**	8.0[-7.7 : 26]	0.121
**30–39**	2010	99.8	4.6 (3.2–7.0)	4.2 (2.8–6.7)	12[-49 : 147]	5.9[-9.7 : 24]	-0.48[-15 : 17]	0.59
**40–49**	2011	99.6	4.3 (2.9–6.9)	4.1 (2.8–6.5)	-56[-84 : 23]	-10[-23 : 5.3]	5.8[-10 : 25]	0.16
**50–59**	1985	99.8	4.5 (3.1–7.7)	4.3 (2.9–6.4)	6.0[-70 : 280]	6.5[-9.6 : 26]	4.0[-12 : 23]	0.841
**60–69**	1872	99.6	4.6 (3.0–7.7)	4.3 (2.9–6.5)	603[-40 : 8106]	46[12 : 90]	5.3[-21 : 40]	0.101

BMI and age-stratified median concentrations

AGE GROUP	Normal BMI		Overweight BMI					Obese BMI				
	**Males** Concentration (pg/ml)	**Females** Concentration (pg/ml)	**Males** Concentration (pg/ml)	**Females** Concentration (pg/ml)	**Males** %change	**Females** %change	**Overweight × sex** Interaction P	**Males** Concentration (pg/ml)	**Females** Concentration (pg/ml)	**Males** %change	**Females** %change	**Obese × sex** Interaction P
**All**	4.3 (3.0–7.3)	4.1 (2.8–6.3)	4.6 (3.1–7.6)	4.1 (2.9–6.6)	4.0[-0.75 : 8.9]	2.3[-3.9 : 8.9]	0.929	4.4 (3.0–6.8)	4.4 (3.0–6.7)	-1.6[-8.1 : 5.4]	3.4[-7.8 : 16]	0.091
**18–29**	4.3 (3.0–7.4)	4.2 (2.9–6.7)	4.3 (3.0–7.3)	3.9 (2.7–6.1)	1.2[-9.5 : 13]	-13[-26 : 1.4]	0.21	5.1 (3.3–7.0)	4.1 (2.8–6.0)	4.4[-14 : 26]	-11[-33 : 18]	0.714
**30–39**	4.4 (3.0–7.2)	4.2 (2.8–6.6)	4.9 (3.4–7.2)	4.2 (2.7–6.5)	9.6[-0.84 : 21]	-4.5[-16 : 9.0]	0.189	4.5 (3.0–6.8)	4.4 (3.0–6.9)	-0.85[-14 : 14]	-1.4[-23 : 26]	0.312
**40–49**	4.4 (2.9–7.4)	3.9 (2.8–6.2)	4.3 (2.9–7.1)	4.0 (2.8–6.8)	-5.4[-14 : 4.6]	7.9[-5.5 : 23]	0.119	4.1 (2.8–6.1)	4.3 (2.9–5.8)	-11[-22 : 2.3]	11[-12 : 39]	0.055
**50–59**	4.2 (2.8–7.4)	4.1 (2.7–6.1)	4.7 (3.2–7.9)	4.1 (3.0–6.3)	10[-0.70 : 22]	6.9[-6.8 : 23]	0.878	4.7 (3.4–7.9)	4.7 (3.3–7.7)	13[-2.8 : 31]	23[-5.3 : 59]	0.249
**60–69**	4.5 (3.2–7.2)	4.3 (2.9–6.0)	4.8 (3.0–8.3)	4.3 (3.0–6.8)	2.6[-8.7 : 15]	8.3[-7.2 : 26]	0.479	3.9 (2.7–6.9)	4.3 (2.6–6.6)	-6.5[-22 : 12]	-8.9[-32 : 22]	0.916

Smoking and age-stratified median concentrations

AGE GROUP	Non-smokers		Smokers		% Change in Concentration smokers vs non-smokers		
	**Males** Concentration (pg/ml)	**Females** Concentration (pg/ml)	**Males** Concentration (pg/ml)	**Females** Concentration (pg/ml)	**Males** change (%)	**Females** change (%)	**Smoking × sex** Interaction P
**All**	4.4 (3.0–7.2)	4.1 (2.8–6.4)	4.8 (3.3–8.5)	4.3 (2.9–6.9)	**11[4.2 : 18]**	4.3[-2.0 : 11]	0.164
**18–29**	4.3 (3.0–7.1)	4.1 (2.8–6.7)	4.4 (3.0–7.5)	3.9 (2.6–5.5)	4.3[-8.8 : 19]	-12[-23 : 1.0]	0.087
**30–39**	4.6 (3.2–7.0)	4.2 (2.8–6.7)	4.7 (3.2–7.3)	4.6 (3.0–6.3)	0.99[-12 : 16]	6.3[-7.2 : 22]	0.603
**40–49**	4.3 (2.9–6.7)	4.0 (2.8–6.3)	5.0 (3.5–8.9)	4.4 (3.2–7.2)	**20[4.5 : 39]**	**17[2.0 : 33]**	0.746
**50–59**	4.4 (2.9–7.4)	4.2 (2.8–6.3)	5.1 (3.5–10)	4.3 (3.1–7.6)	**26[9.5 : 45]**	8.1[-6.1 : 25]	0.132
**60–69**	4.5 (3.0–7.5)	4.3 (2.9–6.4)	4.8 (3.3–8.9)	4.5 (2.9–7.1)	5.1[-11 : 24]	6.5[-9.1 : 25]	0.911

Supplementary Table 5: IL-1β (Interleukin 1 beta, IL1B, IL1F2, Uniprot:P01584)

Sex and age-stratified median concentrations

AGE GROUP	Biomarker/cohort information		Median Concentration			Age Group (% change in concentration)		
	**N measured**	**Detection Range**	**Males**	**Females**	**Sex difference**	**Males**	**Females**	**Sex × age**
		(% in range)	(pg/ml)	(pg/ml)	F relative to M (%)	change/10 years (%)	change/10 years (%)	Interaction P
**All**	9862	53.4	0.15 (0.071–0.30)	0.15 (0.066–0.27)	0.21[-16 : 20]	**8.0[5.0 : 11]**	**6.7[3.8 : 9.7]**	0.543
**18–29**	1989	47.8	0.14 (0.054–0.25)	0.14 (0.066–0.24)	25[-53 : 233]	2.9[-23 : 37]	-3.1[-27 : 28]	0.767
**30–39**	2006	50.4	0.16 (0.067–0.28)	0.14 (0.065–0.25)	4.2[-77 : 367]	15[-15 : 56]	10[-18 : 49]	0.842
**40–49**	2010	51.1	0.16 (0.072–0.31)	0.14 (0.056–0.25)	-22[-88 : 414]	-8.9[-32 : 22]	-6.7[-31 : 26]	0.909
**50–59**	1986	58.4	0.16 (0.077–0.34)	0.16 (0.069–0.32)	-48[-95 : 423]	1.2[-25 : 36]	12[-17 : 51]	0.628
**60–69**	1871	59.9	0.17 (0.086–0.36)	0.16 (0.075–0.33)	-60[-99 : 2130]	8.3[-30 : 68]	26[-21 : 100]	0.641

BMI and age-stratified median concentrations

AGE GROUP	Normal BMI		Overweight BMI					Obese BMI				
	**Males**	**Females**	**Males**	**Females**	**Males**	**Females**	**Overweight × sex**	**Males**	**Females**	**Males**	**Females**	**Obese × sex**
	Concentration (pg/ml)	Concentration (pg/ml)	Concentration (pg/ml)	Concentration (pg/ml)	%change	%change	Interaction P	Concentration (pg/ml)	Concentration (pg/ml)	%change	%change	Interaction P
**All**	0.15 (0.067–0.28)	0.14 (0.063–0.25)	0.16 (0.069–0.32)	0.16 (0.073–0.31)	6.4[-2.1 : 16]	7.1[-4.3 : 20]	0.148	0.19 (0.088–0.38)	0.15 (0.066–0.31)	**34[19 : 52]**	-4.5[-22 : 17]	0.112
**18–29**	0.13 (0.052–0.24)	0.13 (0.065–0.23)	0.14 (0.054–0.24)	0.16 (0.078–0.29)	7.3[-12 : 31]	21[-8.8 : 60]	0.471	0.19 (0.10–0.29)	0.15 (0.068–0.34)	52[7.5 : 115]	45[-13 : 143]	0.778
**30–39**	0.15 (0.066–0.26)	0.13 (0.061–0.24)	0.15 (0.064–0.28)	0.15 (0.069–0.25)	6.0[-12 : 28]	-7.1[-28 : 19]	0.823	0.19 (0.084–0.44)	0.15 (0.079–0.31)	43[9.8 : 86]	-14[-46 : 36]	0.669
**40–49**	0.15 (0.076–0.28)	0.13 (0.058–0.23)	0.16 (0.068–0.31)	0.15 (0.063–0.30)	-4.6[-21 : 15]	13[-11 : 45]	0.064	0.18 (0.084–0.36)	0.14 (0.047–0.25)	6.6[-17 : 37]	-6.4[-39 : 43]	0.667
**50–59**	0.15 (0.067–0.29)	0.15 (0.064–0.31)	0.17 (0.077–0.35)	0.16 (0.071–0.35)	18[-1.6 : 43]	14[-12 : 46]	0.871	0.20 (0.11–0.38)	0.17 (0.081–0.34)	40[7.2 : 83]	1.6[-36 : 62]	0.374
**60–69**	0.17 (0.091–0.36)	0.16 (0.073–0.33)	0.16 (0.085–0.33)	0.19 (0.086–0.34)	1.3[-16 : 23]	0.75[-22 : 30]	0.465	0.18 (0.089–0.46)	0.17 (0.063–0.32)	27[-5.7 : 71]	-25[-54 : 20]	0.174

Smoking and age-stratified median concentrations

AGE GROUP	Non-smokers		Smokers		% Change in Concentration smokers vs non-smokers		
	**Males**	**Females**	**Males**	**Females**	**Males**	**Females**	**Smoking × sex**
	Concentration (pg/ml)	Concentration (pg/ml)	Concentration (pg/ml)	Concentration (pg/ml)	change (%)	change (%)	Interaction P
**All**	0.15 (0.069–0.29)	0.15 (0.066–0.27)	0.17 (0.086–0.37)	0.16 (0.073–0.31)	**24[10 : 39]**	**18[5.8 : 32]**	0.579
**18–29**	0.14 (0.053–0.24)	0.14 (0.066–0.24)	0.15 (0.065–0.28)	0.16 (0.087–0.30)	**28[0.25 : 62]**	21[-4.4 : 54]	0.769
**30–39**	0.15 (0.065–0.28)	0.14 (0.065–0.25)	0.18 (0.092–0.34)	0.15 (0.065–0.25)	**33[2.0 : 73]**	1.3[-22 : 31]	0.15
**40–49**	0.16 (0.072–0.29)	0.14 (0.056–0.25)	0.16 (0.074–0.37)	0.14 (0.065–0.35)	5.4[-19 : 37]	21[-5.4 : 55]	0.448
**50–59**	0.16 (0.074–0.33)	0.15 (0.068–0.32)	0.20 (0.096–0.40)	0.17 (0.077–0.34)	**40[9.2 : 81]**	20[-7.4 : 55]	0.38
**60–69**	0.16 (0.086–0.35)	0.16 (0.074–0.33)	0.19 (0.11–0.44)	0.18 (0.084–0.42)	14[-13 : 49]	**32[2.2 : 72]**	0.419

Supplementary Table 6: IL-1RA (Interleukin-1 receptor antagonist, IL1RN, DIRA, Uniprot:P18510)

Sex and age-stratified median concentrations

AGE GROUP	Biomarker/cohort information		Median Concentration			Age Group (% change in concentration)		
	**N measured**	**Detection Range**	**Males**	**Females**	**Sex difference**	**Males**	**Females**	**Sex × age**
		(% in range)	(pg/ml)	(pg/ml)	F relative to M (%)	change/10 years (%)	change/10 years (%)	Interaction P
**All**	9732	100	272 (203–389)	270 (200–382)	4.4[-2.3 : 12]	1.4[0.34 : 2.4]	0.84[-0.18 : 1.9]	0.466
**18–29**	1972	100	251 (183–363)	256 (186–364)	19[-17 : 71]	-2.5[-12 : 8.3]	-8.7[-18 : 1.2]	0.377
**30–39**	1982	99.9	267 (191–383)	270 (195–387)	48[-16 : 161]	1.7[-9.3 : 14]	-8.0[-18 : 3.1]	0.221
**40–49**	1979	100	272 (208–395)	272 (205–385)	60[-18 : 211]	0.14[-9.7 : 11]	-9.3[-18 : 0.81]	0.187
**50–59**	1960	100	283 (212–403)	270 (208–390)	-4.4[-58 : 119]	3.0[-7.5 : 15]	4.0[-6.6 : 16]	0.899
**60–69**	1839	99.9	288 (215–411)	274 (214–388)	-55[-89 : 94]	-7.3[-21 : 8.6]	5.2[-11 : 24]	0.28

BMI and age-stratified median concentrations

AGE GROUP	Normal BMI		Overweight BMI					Obese BMI				
	**Males**	**Females**	**Males**	**Females**	**Males**	**Females**	**Overweight × sex**	**Males**	**Females**	**Males**	**Females**	**Obese × sex**
	Concentration (pg/ml)	Concentration (pg/ml)	Concentration (pg/ml)	Concentration (pg/ml)	%change	%change	Interaction P	Concentration (pg/ml)	Concentration (pg/ml)	%change	%change	Interaction P
**All**	241 (180–330)	243 (183–328)	286 (215–404)	291 (222–411)	**19[15 : 22]**	4.5[0.28 : 8.8]	0.34	382 (286–531)	388 (284–547)	**56[49 : 64]**	10[2.3 : 19]	0.669
**18–29**	229 (169–320)	237 (175–324)	276 (208–383)	284 (209–421)	**22[13 : 31]**	2.5[-7.5 : 13]	0.537	446 (280–669)	400 (287–569)	**92[69 : 118]**	3.9[-14 : 25]	0.122
**30–39**	231 (173–315)	230 (174–315)	271 (201–391)	287 (223–378)	**20[12 : 29]**	11[0.86 : 22]	0.441	407 (308–579)	431 (312–660)	**73[56 : 91]**	**31[10 : 56]**	0.465
**40–49**	237 (185–321)	248 (187–321)	289 (214–396)	290 (212–427)	**16[8.3 : 24]**	5.7[-3.0 : 15]	0.43	376 (292–522)	374 (281–492)	**55[41 : 69]**	7.6[-7.3 : 25]	0.481
**50–59**	263 (194–343)	245 (189–342)	282 (223–412)	293 (229–403)	**17[9.0 : 25]**	2.3[-6.5 : 12]	0.685	357 (273–487)	381 (268–577)	**43[30 : 57]**	7.4[-9.2 : 27]	0.35
**60–69**	255 (194–352)	251 (199–344)	306 (228–438)	302 (233–422)	**19[11 : 28]**	3.5[-5.5 : 13]	0.991	363 (274–499)	371 (282–529)	**32[19 : 48]**	6.8[-10 : 27]	0.122

Smoking and age-stratified median concentrations

AGE GROUP	Non-smokers		Smokers		% Change in Concentration smokers vs non-smokers		
	**Males**	**Females**	**Males**	**Females**	**Males**	**Females**	**Smoking × sex**
	Concentration (pg/ml)	Concentration (pg/ml)	Concentration (pg/ml)	Concentration (pg/ml)	change (%)	change (%)	Interaction P
**All**	271 (203–385)	268 (199–380)	296 (213–440)	284 (209–400)	**11[6.1 : 15]**	**7.8[3.5 : 12]**	0.383
**18–29**	254 (185–363)	255 (185–357)	255 (180–383)	261 (192–387)	1.9[-6.7 : 11]	4.4[-4.3 : 14]	0.707
**30–39**	269 (193–380)	270 (194–390)	272 (187–440)	273 (209–356)	4.9[-5.0 : 16]	-3.8[-13 : 5.9]	0.217
**40–49**	270 (207–396)	270 (202–381)	305 (230–404)	298 (229–441)	**11[0.77 : 21]**	**13[3.9 : 24]**	0.7
**50–59**	278 (209–397)	270 (207–385)	311 (262–439)	307 (222–442)	**20[9.9 : 32]**	**11[1.5 : 22]**	0.231
**60–69**	286 (213–401)	274 (213–388)	327 (236–511)	298 (220–419)	**19[8.3 : 32]**	**15[4.9 : 27]**	0.61

Supplementary Table 7: IL-3 (Interleukin-3, MCGF, MULTI-CSF, Uniprot:P08700)

Sex and age-stratified median concentrations

AGE GROUP	Biomarker/cohort information		Median Concentration			Age Group (% change in concentration)		
	N measured	Detection Range	Males	Females	Sex difference	Males	Females	Sex × age
		(% in range)	(pg/ml)	(pg/ml)	F relative to M (%)	change/10 years (%)	change/10 years (%)	Interaction P
**All**	9789	28.4	4.1 (2.1–6.2)	4.1 (2.1–6.1)	-7.4[-20 : 6.6]	0.66[-1.5 : 2.9]	1.9[-0.30 : 4.1]	0.439
**18–29**	1978	27.3	4.1 (2.0–6.0)	4.1 (2.1–6.0)	-6.8[-55 : 93]	1.7[-18 : 26]	6.9[-13 : 32]	0.736
**30–39**	1995	26.2	4.1 (2.1–6.1)	3.8 (1.8–5.9)	-63[-88 : 15]	-16[-34 : 5.6]	7.1[-15 : 35]	0.14
**40–49**	1993	27.7	4.3 (2.2–6.2)	4.2 (2.1–6.2)	66[-64 : 656]	-2.0[-22 : 24]	-13[-31 : 11]	0.506
**50–59**	1972	29.4	4.1 (2.1–6.2)	4.1 (2.1–6.1)	-54[-92 : 168]	-10[-28 : 13]	3.0[-18 : 30]	0.404
**60–69**	1851	31.7	4.1 (2.2–6.2)	4.1 (2.1–6.2)	-90[-100 : 166]	-23[-46 : 9.8]	11[-24 : 61]	0.166

BMI and age-stratified median concentrations

AGE GROUP	Normal BMI		Overweight BMI					Obese BMI				
	Males	Females	Males	Females	Males	Females	Overweight × sex	Males	Females	Males	Females	Obese × sex
	Concentration (pg/ml)	Concentration (pg/ml)	Concentration (pg/ml)	Concentration (pg/ml)	%change	%change	Interaction P	Concentration (pg/ml)	Concentration (pg/ml)	%change	%change	Interaction P
**All**	4.1 (2.0–6.1)	4.0 (2.0–6.1)	4.2 (2.1–6.2)	4.1 (2.1–6.1)	5.2[-1.4 : 12]	1.5[-6.9 : 11]	0.173	4.2 (2.0–6.1)	4.1 (2.2–6.2)	0.45[-8.7 : 10]	10[-5.9 : 29]	0.752
**18–29**	4.1 (2.0–6.0)	4.1 (2.0–6.0)	4.1 (1.9–6.0)	4.1 (2.2–5.9)	9.8[-5.5 : 28]	5.5[-14 : 30]	0.659	3.3 (1.6–5.7)	4.3 (2.4–6.2)	-10[-31 : 16]	14[-22 : 67]	0.202
**30–39**	4.1 (2.1–6.0)	3.6 (1.8–5.9)	4.1 (2.0–6.0)	3.8 (1.8–5.8)	1.3[-12 : 17]	13[-7.0 : 37]	0.832	4.3 (2.1–6.3)	4.0 (1.8–6.0)	2.7[-16 : 26]	25[-12 : 78]	0.877
**40–49**	4.2 (2.3–6.2)	4.1 (2.0–6.1)	4.3 (2.4–6.4)	4.1 (2.2–6.2)	11[-3.9 : 29]	12[-8.3 : 36]	0.307	4.6 (2.0–6.3)	4.5 (2.4–6.6)	-1.3[-19 : 21]	56[11 : 119]	0.212
**50–59**	4.0 (1.9–6.1)	4.1 (2.1–6.1)	4.3 (2.3–6.2)	4.1 (2.0–6.2)	7.6[-6.7 : 24]	-2.3[-19 : 18]	0.427	4.1 (2.3–6.1)	4.1 (2.1–5.9)	9.8[-10 : 35]	-5.7[-34 : 35]	0.423
**60–69**	4.0 (2.2–6.2)	4.2 (2.1–6.3)	4.2 (2.3–6.3)	4.1 (2.1–6.2)	-0.97[-15 : 15]	-16[-31 : 3.4]	0.461	4.1 (1.7–5.9)	4.1 (2.1–6.4)	-7.9[-27 : 17]	-24[-48 : 11]	0.948

Smoking and age-stratified median concentrations

AGE GROUP	Non-smokers		Smokers		% Change in Concentration smokers vs non-smokers		
	Males	Females	Males	Females	Males	Females	Smoking × sex
	Concentration (pg/ml)	Concentration (pg/ml)	Concentration (pg/ml)	Concentration (pg/ml)	change (%)	change (%)	Interaction P
**All**	4.1 (2.1–6.1)	4.1 (2.0–6.1)	4.3 (2.2–6.3)	4.0 (2.1–6.0)	4.1[-4.7 : 14]	-0.43[-8.7 : 8.6]	0.478
**18–29**	4.0 (1.9–5.9)	4.1 (2.1–6.0)	4.2 (2.2–6.2)	4.1 (1.8–6.0)	6.6[-11 : 27]	-10[-25 : 7.0]	0.176
**30–39**	4.1 (2.0–6.0)	3.7 (1.8–5.9)	4.6 (2.6–6.8)	3.7 (1.9–5.9)	16[-5.2 : 42]	7.5[-12 : 31]	0.6
**40–49**	4.4 (2.3–6.3)	4.2 (2.1–6.3)	4.2 (1.8–6.0)	4.0 (2.0–5.8)	-6.4[-24 : 16]	-2.4[-20 : 19]	0.778
**50–59**	4.2 (2.1–6.2)	4.1 (2.0–6.1)	4.5 (2.5–5.9)	4.1 (2.4–6.0)	9.1[-10 : 32]	9.5[-10 : 33]	0.979
**60–69**	4.1 (2.3–6.2)	4.1 (2.1–6.3)	4.3 (2.0–6.6)	4.2 (2.1–5.8)	-3.0[-22 : 20]	-4.6[-23 : 18]	0.917

Supplementary Table 8: IL-6 (Interleukin-6, BSF2, HGF, HSF, IFNB2, Uniprot:P05231)

Sex and age-stratified median concentrations

AGE GROUP	Biomarker/cohort information		Median Concentration			Age Group (% change in concentration)		
	N measured	Detection Range	Males	Females	Sex difference	Males	Females	Sex × age
		(% in range)	(pg/ml)	(pg/ml)	F relative to M (%)	change/10 years (%)	change/10 years (%)	Interaction P
**All**	9873	95.6	0.56 (0.34–0.86)	0.58 (0.36–0.92)	**41[26 : 57]**	**16[14 : 18]**	**10[8.3 : 12]**	**2.80E-05**
**18–29**	1994	92.9	0.43 (0.25–0.63)	0.49 (0.30–0.73)	12[-40 : 110]	-17[-31 : -0.70]	-14[-28 : 2.7]	0.778
**30–39**	2010	95.1	0.49 (0.30–0.73)	0.54 (0.32–0.90)	16[-55 : 196]	2.4[-15 : 24]	3.2[-15 : 25]	0.951
**40–49**	2011	95.9	0.55 (0.34–0.83)	0.57 (0.33–0.87)	178[-13 : 788]	28[6.7 : 53]	3.8[-14 : 25]	0.116
**50–59**	1986	96.1	0.63 (0.42–0.96)	0.63 (0.41–1.0)	117[-45 : 751]	29[7.9 : 53]	13[-5.0 : 35]	0.319
**60–69**	1872	97.9	0.75 (0.51–1.1)	0.72 (0.48–1.1)	-59[-96 : 309]	-8.5[-29 : 17]	5.4[-19 : 37]	0.446

BMI and age-stratified median concentrations

AGE GROUP	Normal BMI		Overweight BMI					Obese BMI				
	Males	Females	Males	Females	Males	Females	Overweight × sex	Males	Females	Males	Females	Obese × sex
	Concentration (pg/ml)	Concentration (pg/ml)	Concentration (pg/ml)	Concentration (pg/ml)	%change	%change	Interaction P	Concentration (pg/ml)	Concentration (pg/ml)	%change	%change	Interaction P
**All**	0.47 (0.29–0.70)	0.46 (0.30–0.69)	0.59 (0.39–0.88)	0.69 (0.47–1.0)	**23[17 : 30]**	**13[5.9 : 22]**	**3.83E-07**	0.79 (0.53–1.2)	1.1 (0.71–1.6)	**71[59 : 85]**	20[5.8 : 36]	**4.07E-09**
**18–29**	0.39 (0.23–0.57)	0.43 (0.26–0.60)	0.46 (0.27–0.66)	0.60 (0.37–0.90)	**24[9.1 : 42]**	2.7[-14 : 23]	0.052	0.64 (0.47–1.2)	0.91 (0.65–1.6)	**113[70 : 166]**	10[-21 : 53]	0.156
**30–39**	0.41 (0.25–0.60)	0.40 (0.26–0.63)	0.50 (0.32–0.75)	0.66 (0.43–0.94)	**26[12 : 42]**	**30[11 : 52]**	0.007	0.71 (0.50–1.0)	0.98 (0.68–1.5)	**85[57 : 118]**	44[7.7 : 91]	0.01
**40–49**	0.45 (0.29–0.67)	0.42 (0.27–0.64)	0.56 (0.35–0.82)	0.65 (0.42–0.93)	**26[12 : 41]**	20[3.4 : 39]	0.005	0.78 (0.55–1.1)	1.0 (0.71–1.7)	**71[46 : 101]**	26[-3.0 : 63]	**2.44E-04**
**50–59**	0.54 (0.36–0.85)	0.52 (0.33–0.73)	0.66 (0.45–0.95)	0.74 (0.51–1.1)	**25[12 : 39]**	10[-5.0 : 27]	0.008	0.94 (0.54–1.3)	1.2 (0.87–1.7)	**68[43 : 97]**	11[-16 : 46]	**0.001**
**60–69**	0.63 (0.46–0.99)	0.59 (0.41–0.87)	0.76 (0.53–1.1)	0.77 (0.57–1.2)	10[-1.0 : 23]	9.4[-5.3 : 26]	0.006	0.90 (0.61–1.3)	1.1 (0.74–1.4)	**40[18 : 66]**	15[-12 : 50]	0.005

Smoking and age-stratified median concentrations

AGE GROUP	Non-smokers		Smokers		% Change in Concentration smokers vs non-smokers		
	Males	Females	Males	Females	Males	Females	Smoking × sex
	Concentration (pg/ml)	Concentration (pg/ml)	Concentration (pg/ml)	Concentration (pg/ml)	change (%)	change (%)	Interaction P
**All**	0.54 (0.33–0.83)	0.57 (0.35–0.90)	0.63 (0.40–1.0)	0.66 (0.42–1.1)	**23[15 : 32]**	**22[14 : 31]**	0.82
**18–29**	0.42 (0.25–0.62)	0.48 (0.30–0.70)	0.47 (0.26–0.66)	0.53 (0.33–0.82)	4.9[-10 : 22]	4.2[-11 : 21]	0.951
**30–39**	0.48 (0.29–0.73)	0.52 (0.31–0.85)	0.52 (0.34–0.71)	0.63 (0.41–1.2)	5.8[-10 : 25]	**21[3.2 : 42]**	0.247
**40–49**	0.54 (0.34–0.80)	0.56 (0.32–0.85)	0.63 (0.45–0.97)	0.59 (0.40–1.1)	**28[9.4 : 51]**	**18[1.2 : 37]**	0.441
**50–59**	0.61 (0.42–0.93)	0.61 (0.40–0.98)	0.81 (0.51–1.2)	0.77 (0.53–1.2)	**29[11 : 50]**	**38[18 : 60]**	0.566
**60–69**	0.69 (0.49–1.0)	0.69 (0.46–1.0)	0.98 (0.69–1.6)	0.79 (0.57–1.2)	**58[35 : 84]**	**28[10 : 48]**	0.048

Supplementary Table 9: IL-12/IL-23p40 (Interleukin-12 subunit beta, CLMF, IL12B, NKSF, Uniprot:P29460)

Sex and age-stratified median concentrations

AGE GROUP	Biomarker/cohort information		Median Concentration			Age Group (% change in concentration)		
	**N measured**	**Detection Range** (% in range)	**Males** (pg/ml)	**Females** (pg/ml)	**Sex difference** F relative to M (%)	**Males** change/10 years (%)	**Females** change/10 years (%)	**Sex × age** Interaction P
**All**	9872	100	116 (88–151)	131 (99–176)	**12[5.6 : 19]**	**-4.9[-5.7 : -4.0]**	**-4.3[-5.1 : -3.4]**	0.337
**18–29**	1994	100	125 (99–161)	147 (111–187)	-0.62[-26 : 34]	**-15[-22 : -6.7]**	-9.1[-17 : -0.91]	0.326
**30–39**	2010	100	122 (92–157)	136 (102–184)	58[-3.0 : 156]	-6.8[-16 : 2.7]	**-15[-23 : -6.4]**	0.188
**40–49**	2011	100	113 (85–146)	125 (96–167)	13[-37 : 103]	-5.2[-13 : 3.8]	-5.2[-14 : 4.2]	0.997
**50–59**	1985	100	110 (81–146)	125 (95–166)	-24[-63 : 58]	-1.5[-10 : 8.2]	6.4[-3.1 : 17]	0.254
**60–69**	1872	99.9	110 (84–143)	125 (94–170)	-0.23[-77 : 331]	5.1[-10 : 23]	8.1[-8.6 : 28]	0.811

BMI and age-stratified median concentrations

AGE GROUP	Normal BMI		Overweight BMI					Obese BMI				
	**Males** Concentration (pg/ml)	**Females** Concentration (pg/ml)	**Males** Concentration (pg/ml)	**Females** Concentration (pg/ml)	**Males** %change	**Females** %change	**Overweight × sex** Interaction P	**Males** Concentration (pg/ml)	**Females** Concentration (pg/ml)	**Males** %change	**Females** %change	**Obese × sex** Interaction P
**All**	111 (85–144)	126 (95–168)	118 (89–153)	134 (101–176)	**7.9[5.0 : 11]**	3.2[-0.47 : 7.0]	0.887	125 (97–167)	148 (115–199)	**18[13 : 23]**	6.5[-0.33 : 14]	0.64
**18–29**	122 (95–152)	145 (111–184)	129 (103–175)	150 (110–188)	**12[5.0 : 19]**	2.8[-5.7 : 12]	0.406	142 (104–186)	149 (114–203)	16[3.9 : 29]	-1.3[-16 : 16]	0.44
**30–39**	114 (88–149)	131 (98–175)	124 (93–163)	135 (104–182)	6.4[0.11 : 13]	-3.1[-11 : 5.1]	0.317	124 (103–171)	150 (114–205)	**16[6.1 : 26]**	3.5[-11 : 20]	0.798
**40–49**	106 (78–134)	119 (90–163)	113 (86–144)	130 (96–167)	**9.5[3.4 : 16]**	1.1[-6.3 : 9.0]	0.615	129 (97–173)	139 (114–173)	**29[19 : 39]**	2.1[-10 : 16]	0.117
**50–59**	102 (73–135)	118 (89–150)	112 (82–147)	129 (100–170)	7.8[1.7 : 14]	7.5[-0.68 : 16]	0.237	119 (94–155)	146 (111–200)	**20[10 : 30]**	10[-4.9 : 28]	0.493
**60–69**	104 (82–130)	119 (87–156)	112 (85–147)	130 (100–173)	8.4[1.2 : 16]	4.8[-4.4 : 15]	0.409	116 (92–156)	158 (119–208)	14[2.0 : 27]	9.7[-7.7 : 30]	0.064

Smoking and age-stratified median concentrations

AGE GROUP	Non-smokers		Smokers		% Change in Concentration smokers vs non-smokers		
	**Males** Concentration (pg/ml)	**Females** Concentration (pg/ml)	**Males** Concentration (pg/ml)	**Females** Concentration (pg/ml)	**Males** change (%)	**Females** change (%)	**Smoking × sex** Interaction P
**All**	119 (91–154)	136 (104–180)	92 (69–124)	100 (74–138)	**-22[-25 : -20]**	**-27[-29 : -24]**	0.037
**18–29**	128 (101–163)	151 (117–192)	107 (81–142)	118 (91–154)	**-17[-23 : -10]**	**-24[-29 : -18]**	0.105
**30–39**	124 (95–163)	138 (106–187)	101 (77–127)	104 (81–150)	**-22[-28 : -15]**	**-23[-30 : -17]**	0.754
**40–49**	117 (89–149)	129 (100–170)	81 (65–113)	102 (70–130)	**-26[-32 : -19]**	**-26[-31 : -20]**	0.955
**50–59**	115 (86–149)	129 (100–169)	78 (61–106)	93 (69–131)	**-30[-36 : -25]**	**-27[-33 : -21]**	0.407
**60–69**	112 (87–145)	131 (100–176)	90 (67–125)	85 (65–116)	**-18[-26 : -9.6]**	**-33[-39 : -27]**	**0.003**

Supplementary Table 10: IL-12p70 (Interleukin-12, Uniprot:P29459)

Sex and age-stratified median concentrations

AGE GROUP	Biomarker/cohort information		Median Concentration			Age Group (% change in concentration)		
	N measured	Detection Range (% in range)	Males (pg/ml)	Females (pg/ml)	Sex difference F relative to M (%)	Males change/10 years (%)	Females change/10 years (%)	Sex × age Interaction P
**All**	9873	67	0.15 (0.076–0.27)	0.15 (0.077–0.26)	-14[-27 : 0.93]	-2.8[-5.3 : -0.34]	0.58[-1.9 : 3.1]	0.055
**18–29**	1994	69.5	0.15 (0.077–0.27)	0.15 (0.075–0.25)	-38[-73 : 43]	-13[-32 : 11]	2.6[-19 : 30]	0.336
**30–39**	2010	68.3	0.15 (0.076–0.27)	0.15 (0.077–0.26)	14[-71 : 345]	0.040[-24 : 32]	-4.2[-27 : 26]	0.824
**40–49**	2011	67.8	0.16 (0.079–0.27)	0.15 (0.069–0.26)	709[45 : 4398]	29[-1.1 : 68]	-20[-39 : 5.1]	0.014
**50–59**	1986	65.6	0.15 (0.080–0.27)	0.15 (0.079–0.25)	-6.0[-89 : 695]	-11[-32 : 18]	-10[-32 : 18]	0.987
**60–69**	1872	63.8	0.15 (0.070–0.26)	0.16 (0.082–0.26)	21[-97 : 5097]	34[-11 : 101]	33[-14 : 105]	0.983

BMI and age-stratified median concentrations

AGE GROUP	Normal BMI		Overweight BMI					Obese BMI				
	Males Concentration (pg/ml)	Females Concentration (pg/ml)	Males Concentration (pg/ml)	Females Concentration (pg/ml)	Males %change	Females %change	Overweight × sex Interaction P	Males Concentration (pg/ml)	Females Concentration (pg/ml)	Males %change	Females %change	Obese × sex Interaction P
**All**	0.16 (0.079–0.27)	0.15 (0.075–0.25)	0.15 (0.072–0.26)	0.15 (0.079–0.26)	-10.[-16 : -2.9]	11[0.060 : 23]	**7.57E-04**	0.14 (0.074–0.27)	0.15 (0.073–0.26)	-9.4[-19 : 1.2]	9.7[-8.8 : 32]	0.066
**18–29**	0.16 (0.081–0.29)	0.14 (0.073–0.25)	0.14 (0.071–0.24)	0.15 (0.082–0.25)	-19[-32 : -4.4]	7.2[-15 : 36]	0.012	0.15 (0.081–0.27)	0.14 (0.062–0.27)	-0.010[-25 : 34]	-4.1[-38 : 48]	0.854
**30–39**	0.16 (0.079–0.28)	0.15 (0.077–0.26)	0.15 (0.063–0.24)	0.17 (0.080–0.28)	-7.4[-22 : 10]	14[-9.0 : 43]	0.033	0.15 (0.086–0.28)	0.15 (0.073–0.23)	0.96[-21 : 28]	-16[-45 : 27]	0.913
**40–49**	0.16 (0.078–0.26)	0.14 (0.069–0.26)	0.16 (0.082–0.28)	0.16 (0.074–0.29)	-6.0[-21 : 11]	30[3.8 : 62]	0.072	0.14 (0.069–0.26)	0.14 (0.065–0.27)	-22[-38 : -1.1]	23[-16 : 81]	0.174
**50–59**	0.15 (0.081–0.26)	0.15 (0.077–0.25)	0.16 (0.079–0.27)	0.15 (0.075–0.23)	-5.5[-20 : 12]	-5.2[-25 : 19]	0.828	0.13 (0.064–0.27)	0.17 (0.088–0.33)	-17[-35 : 6.2]	28[-17 : 96]	0.045
**60–69**	0.15 (0.076–0.25)	0.15 (0.078–0.25)	0.14 (0.064–0.24)	0.15 (0.082–0.25)	-7.6[-23 : 10]	7.2[-15 : 36]	0.478	0.17 (0.080–0.29)	0.16 (0.096–0.25)	9.3[-17 : 44]	20[-23 : 86]	0.831

Smoking and age-stratified median concentrations

AGE GROUP	Non-smokers		Smokers		% Change in Concentration smokers vs non-smokers		
	Males Concentration (pg/ml)	Females Concentration (pg/ml)	Males Concentration (pg/ml)	Females Concentration (pg/ml)	Males change (%)	Females change (%)	Smoking × sex Interaction P
**All**	0.15 (0.077–0.27)	0.15 (0.076–0.26)	0.15 (0.065–0.27)	0.15 (0.078–0.27)	-6.0[-15 : 4.2]	4.7[-5.3 : 16]	0.142
**18–29**	0.15 (0.078–0.27)	0.15 (0.073–0.26)	0.15 (0.066–0.26)	0.15 (0.075–0.24)	-11[-27 : 9.4]	-2.9[-21 : 19]	0.562
**30–39**	0.15 (0.074–0.27)	0.15 (0.076–0.26)	0.17 (0.076–0.27)	0.16 (0.087–0.29)	8.7[-14 : 38]	15[-8.6 : 46]	0.725
**40–49**	0.16 (0.081–0.27)	0.15 (0.067–0.27)	0.16 (0.072–0.28)	0.14 (0.081–0.24)	-13[-31 : 11]	16[-7.0 : 46]	0.083
**50–59**	0.15 (0.082–0.27)	0.15 (0.080–0.25)	0.13 (0.054–0.24)	0.14 (0.066–0.24)	-16[-33 : 6.5]	-13[-31 : 11]	0.834
**60–69**	0.15 (0.072–0.24)	0.15 (0.081–0.24)	0.14 (0.063–0.28)	0.16 (0.080–0.31)	2.3[-20 : 32]	20[-6.1 : 52]	0.38

Supplementary Table 11: IL-15 (Interleukin-15, Uniprot:P40933)

Sex and age-stratified median concentrations

AGE GROUP	Biomarker/cohort information		Median Concentration			Age Group (% change in concentration)		
	**N measured**	**Detection Range**	**Males**	**Females**	**Sex difference**	**Males**	**Females**	**Sex × age**
		(% in range)	(pg/ml)	(pg/ml)	F relative to M (%)	change/10 years (%)	change/10 years (%)	Interaction P
**All**	9872	100	2.6 (2.2–2.9)	2.5 (2.2–2.9)	3.3[0.30 : 6.5]	**3.3[2.8 : 3.8]**	**2.2[1.7 : 2.7]**	**0.001**
**18–29**	1994	100	2.4 (2.1–2.7)	2.5 (2.2–2.8)	15[-1.5 : 34]	**7.1[2.4 : 12]**	1.4[-2.9 : 6.0]	0.086
**30–39**	2010	100	2.5 (2.2–2.8)	2.5 (2.2–2.8)	14[-13 : 49]	5.2[-0.37 : 11]	1.1[-4.2 : 6.7]	0.313
**40–49**	2011	100	2.6 (2.2–2.9)	2.6 (2.2–2.9)	-6.8[-30 : 24]	1.9[-2.5 : 6.5]	3.1[-1.5 : 8.0]	0.717
**50–59**	1985	100	2.6 (2.3–3.0)	2.6 (2.3–3.0)	15[-18 : 63]	3.4[-1.1 : 8.1]	0.29[-4.1 : 4.8]	0.338
**60–69**	1872	99.9	2.7 (2.4–3.0)	2.6 (2.3–3.0)	-32[-68 : 42]	-3.2[-11 : 5.0]	2.4[-5.9 : 11]	0.347

BMI and age-stratified median concentrations

AGE GROUP	Normal BMI		Overweight BMI					Obese BMI				
	**Males**	**Females**	**Males**	**Females**	**Males**	**Females**	**Overweight × sex**	**Males**	**Females**	**Males**	**Females**	**Obese × sex**
	Concentration (pg/ml)	Concentration (pg/ml)	Concentration (pg/ml)	Concentration (pg/ml)	%change	%change	Interaction P	Concentration (pg/ml)	Concentration (pg/ml)	%change	%change	Interaction P
**All**	2.6 (2.2–2.9)	2.5 (2.2–2.9)	2.6 (2.3–2.9)	2.5 (2.2–2.9)	-0.13[-1.5 : 1.2]	-2.1[-3.9 : -0.25]	0.263	2.5 (2.2–2.9)	2.5 (2.2–2.8)	-0.77[-2.8 : 1.2]	**-5.0[-8.2 : -1.8]**	0.097
**18–29**	2.4 (2.2–2.8)	2.5 (2.2–2.8)	2.4 (2.1–2.8)	2.5 (2.1–2.8)	-0.65[-3.7 : 2.5]	-4.7[-8.8 : -0.43]	0.452	2.3 (2.1–2.7)	2.5 (2.1–2.7)	-3.4[-8.4 : 2.0]	-8.0[-15 : -0.37]	0.878
**30–39**	2.5 (2.1–2.8)	2.5 (2.2–2.8)	2.5 (2.2–2.8)	2.5 (2.1–2.7)	1.3[-2.1 : 4.8]	-4.5[-8.7 : -0.10]	0.032	2.4 (2.1–2.7)	2.4 (2.1–2.7)	-0.59[-5.2 : 4.2]	-4.6[-12 : 3.6]	0.38
**40–49**	2.6 (2.2–3.0)	2.5 (2.2–2.9)	2.5 (2.3–2.9)	2.6 (2.2–2.9)	-1.2[-3.9 : 1.6]	0.36[-3.3 : 4.2]	0.353	2.6 (2.2–2.9)	2.5 (2.2–2.8)	-0.81[-4.6 : 3.1]	-3.0[-9.1 : 3.4]	0.628
**50–59**	2.7 (2.3–3.0)	2.6 (2.3–3.0)	2.7 (2.3–3.0)	2.6 (2.3–3.0)	-0.80[-3.5 : 2.0]	-0.60[-4.2 : 3.2]	0.569	2.6 (2.3–3.0)	2.5 (2.2–2.9)	-0.52[-4.4 : 3.5]	-5.9[-12 : 0.88]	0.239
**60–69**	2.7 (2.4–3.1)	2.6 (2.3–3.0)	2.7 (2.4–3.0)	2.6 (2.3–3.0)	0.13[-3.3 : 3.7]	-0.65[-5.2 : 4.1]	0.784	2.6 (2.4–3.2)	2.6 (2.3–3.0)	1.3[-4.1 : 7.0]	-3.1[-11 : 5.8]	0.274

Smoking and age-stratified median concentrations

AGE GROUP	Non-smokers		Smokers		% Change in Concentration smokers vs non-smokers		
	**Males**	**Females**	**Males**	**Females**	**Males**	**Females**	**Smoking × sex**
	Concentration (pg/ml)	Concentration (pg/ml)	Concentration (pg/ml)	Concentration (pg/ml)	change (%)	change (%)	Interaction P
**All**	2.5 (2.2–2.9)	2.5 (2.2–2.8)	2.7 (2.4–3.1)	2.7 (2.4–3.1)	**7.5[5.5 : 9.5]**	**8.9[6.9 : 11]**	0.337
**18–29**	2.4 (2.1–2.7)	2.5 (2.1–2.8)	2.6 (2.2–2.8)	2.5 (2.2–2.9)	3.8[-0.040 : 7.8]	**4.7[0.86 : 8.6]**	0.755
**30–39**	2.4 (2.1–2.7)	2.4 (2.1–2.7)	2.7 (2.3–3.0)	2.6 (2.2–2.9)	**8.7[3.8 : 14]**	**8.2[3.3 : 13]**	0.873
**40–49**	2.5 (2.2–2.9)	2.5 (2.2–2.8)	2.8 (2.4–3.1)	2.7 (2.4–3.0)	**7.8[3.6 : 12]**	**7.5[3.6 : 12]**	0.942
**50–59**	2.6 (2.3–3.0)	2.6 (2.3–2.9)	2.8 (2.5–3.2)	2.9 (2.5–3.3)	**6.9[3.0 : 11]**	**13[8.9 : 17]**	0.038
**60–69**	2.7 (2.4–3.0)	2.6 (2.3–3.0)	2.9 (2.6–3.4)	3.0 (2.5–3.4)	**11[5.7 : 17]**	**11[5.8 : 16]**	0.972

Supplementary Table 12: SAA (Serum amyloid A, Uniprot:P0DJI8/9)

Sex and age-stratified median concentrations

AGE GROUP	Biomarker/cohort information		Median Concentration			Age Group (% change in concentration)		
	N measured	Detection Range (% in range)	Males (mg/l)	Females (mg/l)	Sex difference F relative to M (%)	Males change/10 years (%)	Females change/10 years (%)	Sex × age Interaction P
**All**	9503	99.8	4.0 (2.4–6.6)	5.7 (3.6–10.)	**65[47 : 85]**	**5.8[4.0 : 7.8]**	**3.3[1.5 : 5.2]**	0.059
**18–29**	1879	99.7	3.1 (1.8–5.7)	5.7 (3.3–10)	121[12 : 336]	1.5[-17 : 24]	-7.2[-24 : 13]	0.523
**30–39**	1920	99.7	3.6 (2.2–6.0)	5.1 (3.0–8.9)	42[-46 : 277]	-7.6[-24 : 12]	-8.5[-25 : 11]	0.944
**40–49**	1939	99.9	4.2 (2.5–6.7)	5.2 (3.2–9.2)	329[36 : 1252]	22[2.1 : 45]	-6.4[-22 : 13]	0.042
**50–59**	1928	99.9	4.3 (2.7–6.8)	6.2 (4.0–10)	26[-70 : 429]	-4.1[-20 : 15]	-1.4[-18 : 19]	0.831
**60–69**	1837	99.8	4.4 (2.8–7.1)	6.5 (4.3–11)	-11[-92 : 908]	-4.7[-27 : 24]	4.2[-21 : 38]	0.651

BMI and age-stratified median concentrations

AGE GROUP	Normal BMI		Overweight BMI					Obese BMI				
	Males	Females	Males	Females	Males	Females	Overweight × sex	Males	Females	Males	Females	Obese × sex
	Concentration (mg/l)	Concentration (mg/l)	Concentration (mg/l)	Concentration (mg/l)	%change	%change	Interaction P	Concentration (mg/l)	Concentration (mg/l)	%change	%change	Interaction P
**All**	3.1 (1.9–5.4)	4.8 (3.0–8.0)	4.4 (2.8–6.9)	6.6 (4.2–11)	**31[24 : 38]**	4.4[-2.8 : 12]	0.799	**5.7 (3.7–8.9)**	8.9 (5.6–14)	**71[58 : 86]**	1.0[-11 : 15]	0.44
**18–29**	2.8 (1.7–5.0)	5.1 (3.0–8.8)	4.1 (2.2–6.6)	6.9 (4.2–12)	**36[18 : 56]**	-1.2[-19 : 20]	0.718	4.6 (3.1–7.6)	9.9 (6.1–18)	**52[20 : 93]**	15[-19 : 64]	0.024
**30–39**	2.9 (1.9–4.9)	4.5 (2.5–7.4)	3.7 (2.5–5.9)	5.6 (3.3–9.4)	**25[11 : 42]**	1.3[-14 : 19]	0.619	5.6 (3.4–9.3)	7.3 (5.1–13)	**93[62 : 130]**	-7.9[-32 : 24]	0.617
**40–49**	3.1 (2.0–5.4)	4.4 (2.7–7.1)	4.6 (2.9–6.8)	5.5 (3.4–8.9)	**35[20 : 51]**	5.7[-8.9 : 23]	0.399	5.9 (3.9–8.7)	9.2 (5.6–13)	**67[43 : 94]**	22[-5.4 : 58]	0.277
**50–59**	3.6 (2.3–6.0)	5.2 (3.3–8.6)	4.5 (3.1–7.0)	7.3 (4.7–12)	**23[9.5 : 38]**	6.4[-8.9 : 24]	0.263	5.7 (3.9–9.6)	8.2 (5.6–15)	**53[29 : 81]**	-1.3[-26 : 32]	0.34
**60–69**	3.5 (2.3–5.7)	4.9 (3.6–8.3)	4.8 (3.1–7.3)	7.8 (5.1–12)	**30[15 : 46]**	9.2[-6.2 : 27]	0.12	5.9 (3.9–9.3)	10 (6.7–14)	**78[48 : 113]**	-13[-34 : 16]	0.946

Smoking and age-stratified median concentrations

AGE GROUP	Non-smokers		Smokers		% Change in Concentration smokers vs non-smokers		
	Males Concentration (mg/l)	Females Concentration (mg/l)	Males Concentration (mg/l)	Females Concentration (mg/l)	Males change (%)	Females change (%)	Smoking × sex Interaction P
**All**	4.0 (2.4–6.5)	5.8 (3.6–10)	4.1 (2.4–7.1)	5.3 (3.3–8.7)	3.1[-4.2 : 11]	**-10[-16 : -3.6]**	0.008
**18–29**	3.1 (1.8–5.8)	5.8 (3.3–11)	3.3 (1.7–5.6)	5.5 (3.0–8.9)	-6.2[-21 : 11]	**-17[-30 : -2.3]**	0.304
**30–39**	3.6 (2.2–5.9)	5.1 (3.0–8.9)	3.6 (2.2–5.9)	5.2 (3.1–8.8)	5.6[-11 : 25]	-0.31[-16 : 18]	0.64
**40–49**	4.2 (2.5–6.7)	5.3 (3.2–9.3)	4.3 (2.9–7.3)	4.8 (3.2–8.3)	6.3[-9.3 : 25]	-9.7[-22 : 5.2]	0.145
**50–59**	4.4 (2.7–6.6)	6.2 (4.0–11)	4.5 (2.9–7.4)	5.6 (3.4–9.3)	1.7[-13 : 19]	-9.0[-22 : 6.6]	0.326
**60–69**	4.4 (2.8–7.1)	6.8 (4.4–11)	4.8 (3.0–8.3)	5.0 (3.9–8.1)	12[-5.1 : 31]	**-16[-28 : -2.2]**	0.012

Supplementary Table 13: TNFα (Tumor Necrosis Factor-alpha, TNF, DIF, TNFSF2, TNLG1F, Uniprot:P01375)

Sex and age-stratified median concentrations

AGE GROUP	Biomarker/cohort information		Median Concentration			Age Group (% change in concentration)		
	N measured	Detection Range (% in range)	Males (pg/ml)	Females (pg/ml)	Sex difference F relative to M (%)	Males change/10 years (%)	Females change/10 years (%)	Sex × age Interaction P
**All**	9795	100	2.6 (2.2–3.1)	2.5 (2.1–2.9)	**-13[-16 : -9.6]**	0.60[-0.010 : 1.2]	**2.2[1.6 : 2.8]**	**2.42E-04**
**18–29**	1982	100	2.6 (2.2–3.0)	2.4 (2.0–2.8)	-12[-29 : 8.1]	-6.9[-12 : -1.1]	-5.2[-11 : 0.69]	0.662
**30–39**	1994	100	2.6 (2.3–3.1)	2.4 (2.0–2.9)	20[-13 : 65]	-1.1[-7.2 : 5.4]	-8.4[-14 : -2.3]	0.096
**40–49**	1997	100	2.6 (2.2–3.1)	2.3 (2.0–2.7)	-15[-42 : 25]	-0.10[-5.9 : 6.0]	1.0[-5.1 : 7.5]	0.797
**50–59**	1964	100	2.7 (2.2–3.2)	2.5 (2.1–3.0)	-10[-47 : 52]	5.6[-1.3 : 13]	6.6[-0.37 : 14]	0.838
**60–69**	1858	99.9	2.8 (2.3–3.3)	2.7 (2.2–3.2)	-12[-64 : 113]	4.7[-4.9 : 15]	6.6[-3.7 : 18]	0.793

BMI and age-stratified median concentrations

AGE GROUP	Normal BMI		Overweight BMI					Obese BMI				
	Males Concentration (pg/ml)	Females Concentration (pg/ml)	Males Concentration (pg/ml)	Females Concentration (pg/ml)	Males %change	Females %change	Overweight × sex Interaction P	Males Concentration (pg/ml)	Females Concentration (pg/ml)	Males %change	Females %change	Obese × sex Interaction P
**All**	2.6 (2.2–3.0)	2.3 (2.0–2.8)	2.7 (2.3–3.1)	2.5 (2.1–3.0)	**4.2[2.3 : 6.1]**	2.9[0.39 : 5.4]	0.029	2.9 (2.4–3.4)	2.7 (2.3–3.2)	**10[7.5 : 13]**	6.6[1.9 : 11]	**4.87E-04**
**18–29**	2.6 (2.2–2.9)	2.3 (2.0–2.7)	2.6 (2.3–3.1)	2.4 (2.1–2.8)	4.5[0.040 : 9.1]	-1.2[-7.0 : 4.9]	0.905	2.9 (2.5–3.4)	2.7 (2.2–3.1)	**15[6.7 : 24]**	1.3[-9.2 : 13]	0.904
**30–39**	2.6 (2.2–3.0)	2.3 (1.9–2.7)	2.6 (2.3–3.0)	2.5 (2.1–2.9)	3.7[-0.34 : 8.0]	2.8[-2.5 : 8.4]	0.202	2.9 (2.4–3.4)	2.8 (2.3–3.3)	**12[5.6 : 18]**	8.3[-1.6 : 19]	0.017
**40–49**	2.5 (2.1–2.9)	2.2 (1.9–2.6)	2.7 (2.2–3.1)	2.3 (2.0–2.8)	**7.1[3.1 : 11]**	3.7[-1.4 : 9.0]	0.538	2.9 (2.5–3.4)	2.6 (2.2–3.0)	**17[11 : 23]**	5.2[-3.5 : 15]	0.643
**50–59**	2.6 (2.2–3.2)	2.4 (2.1–2.8)	2.7 (2.3–3.2)	2.6 (2.2–3.1)	2.0[-2.2 : 6.4]	5.1[-0.68 : 11]	0.024	2.7 (2.4–3.2)	2.8 (2.4–3.4)	4.5[-1.6 : 11]	10[-0.97 : 22]	**9.81E-04**
**60–69**	2.7 (2.2–3.1)	2.6 (2.2–3.0)	2.8 (2.4–3.3)	2.7 (2.3–3.3)	**7.5[3.0 : 12]**	0.77[-4.7 : 6.5]	0.635	2.9 (2.5–3.4)	3.0 (2.5–3.6)	9.5[2.5 : 17]	5.0[-5.4 : 17]	0.105

Smoking and age-stratified median concentrations

AGE GROUP	Non-smokers		Smokers		% Change in Concentration smokers vs non-smokers		
	Males Concentration (pg/ml)	Females Concentration (pg/ml)	Males Concentration (pg/ml)	Females Concentration (pg/ml)	Males change (%)	Females change (%)	Smoking × sex Interaction P
**All**	2.6 (2.2–3.1)	2.5 (2.1–2.9)	2.7 (2.3–3.2)	2.5 (2.1–2.9)	1.3[-1.2 : 3.8]	1.4[-1.0 : 3.9]	0.951
**18–29**	2.6 (2.2–3.0)	2.4 (2.0–2.8)	2.6 (2.2–3.0)	2.3 (1.9–2.7)	-0.80[-5.8 : 4.4]	-2.8[-7.6 : 2.3]	0.581
**30–39**	2.6 (2.2–3.1)	2.4 (2.0–2.9)	2.6 (2.3–3.2)	2.4 (1.9–2.8)	0.86[-4.6 : 6.6]	-4.3[-9.3 : 1.0]	0.182
**40–49**	2.6 (2.2–3.1)	2.3 (1.9–2.7)	2.7 (2.3–3.2)	2.4 (2.0–2.7)	2.1[-3.2 : 7.7]	**6.7[1.5 : 12]**	0.23
**50–59**	2.7 (2.3–3.1)	2.5 (2.1–2.9)	2.7 (2.2–3.2)	2.6 (2.1–3.1)	-0.26[-5.8 : 5.6]	**6.7[0.69 : 13]**	0.104
**60–69**	2.8 (2.3–3.3)	2.7 (2.2–3.2)	2.8 (2.4–3.5)	2.7 (2.3–3.1)	4.7[-1.3 : 11]	1.5[-4.1 : 7.5]	0.462

Supplementary Table 14: TNFβ (Tumor Necrosis Factor-beta, Lymphotoxin alpha, LTA, TNFSF1, TNLG1E, Uniprot:P01374)

Sex and age-stratified median concentrations

AGE GROUP	Biomarker/cohort information		Median Concentration			Age Group (% change in concentration)		
	N measured	Detection Range (% in range)	Males (pg/ml)	Females (pg/ml)	Sex difference F relative to M (%)	Males change/10 years (%)	Females change/10 years (%)	Sex × age Interaction P
**All**	9872	98.3	0.24 (0.17–0.32)	0.23 (0.16–0.30)	**-17[-24 : -9.7]**	**-6.7[-8.0 : -5.4]**	**-3.8[-5.1 : -2.4]**	**0.002**
**18–29**	1994	99	0.26 (0.20–0.35)	0.25 (0.18–0.32)	-4.6[-37 : 45]	-7.9[-18 : 4.0]	-8.7[-19 : 3.0]	0.921
**30–39**	2010	99	0.25 (0.19–0.33)	0.23 (0.17–0.30)	25[-37 : 148]	-2.6[-15 : 12]	-11[-23 : 1.6]	0.338
**40–49**	2011	98	0.23 (0.17–0.31)	0.21 (0.15–0.28)	14[-56 : 195]	6.3[-8.2 : 23]	1.2[-13 : 18]	0.645
**50–59**	1985	97.7	0.23 (0.16–0.30)	0.22 (0.15–0.29)	-65[-90 : 23]	-8.0[-22 : 8.2]	9.6[-6.8 : 29]	0.136
**60–69**	1872	97.7	0.22 (0.15–0.29)	0.23 (0.16–0.31)	-43[-93 : 356]	-3.2[-23 : 21]	7.4[-15 : 36]	0.534

BMI and age-stratified median concentrations

AGE GROUP	Normal BMI		Overweight BMI					Obese BMI				
	Males Concentration (pg/ml)	Females Concentration (pg/ml)	Males Concentration (pg/ml)	Females Concentration (pg/ml)	Males %change	Females %change	Overweight × sex Interaction P	Males Concentration (pg/ml)	Females Concentration (pg/ml)	Males %change	Females %change	Obese × sex Interaction P
**All**	0.23 (0.17–0.31)	0.23 (0.16–0.30)	0.24 (0.17–0.32)	0.23 (0.17–0.31)	5.2[0.96 : 9.5]	4.5[-1.1 : 10]	0.912	0.25 (0.19–0.33)	0.23 (0.17–0.31)	**10[3.9 : 17]**	3.4[-6.5 : 14]	0.322
**18–29**	0.26 (0.19–0.34)	0.25 (0.18–0.32)	0.27 (0.20–0.35)	0.24 (0.19–0.32)	3.2[-5.3 : 12]	-4.8[-16 : 7.2]	0.658	0.28 (0.22–0.37)	0.25 (0.19–0.33)	11[-3.9 : 29]	-2.8[-22 : 21]	0.882
**30–39**	0.25 (0.19–0.33)	0.23 (0.16–0.29)	0.26 (0.19–0.34)	0.24 (0.18–0.32)	3.6[-5.0 : 13]	15[2.5 : 29]	0.211	0.27 (0.20–0.35)	0.23 (0.18–0.31)	12[-0.42 : 27]	16[-6.2 : 43]	0.691
**40–49**	0.22 (0.16–0.28)	0.21 (0.15–0.28)	0.25 (0.18–0.33)	0.21 (0.15–0.29)	**15[4.9 : 26]**	9.3[-3.4 : 24]	0.425	0.25 (0.18–0.31)	0.22 (0.16–0.29)	19[4.6 : 35]	6.2[-14 : 31]	0.18
**50–59**	0.21 (0.14–0.29)	0.21 (0.15–0.28)	0.23 (0.17–0.30)	0.22 (0.16–0.30)	7.0[-3.3 : 18]	3.0[-10 : 18]	0.666	0.25 (0.19–0.34)	0.24 (0.15–0.30)	16[0.63 : 34]	-5.5[-27 : 22]	0.066
**60–69**	0.21 (0.15–0.27)	0.22 (0.16–0.31)	0.22 (0.15–0.30)	0.23 (0.17–0.31)	1.6[-7.9 : 12]	-6.9[-18 : 6.0]	0.917	0.22 (0.15–0.28)	0.24 (0.18–0.33)	-0.090[-14 : 17]	-5.7[-26 : 20]	0.129

Smoking and age-stratified median concentrations

AGE GROUP	Non-smokers		Smokers		% Change in Concentration smokers vs non-smokers		
	Males Concentration (pg/ml)	Females Concentration (pg/ml)	Males Concentration (pg/ml)	Females Concentration (pg/ml)	Males change (%)	Females change (%)	Smoking × sex Interaction P
**All**	0.24 (0.17–0.32)	0.23 (0.16–0.31)	0.23 (0.16–0.30)	0.21 (0.15–0.28)	**-11[-16 : -6.0]**	**-7.2[-12 : -1.9]**	0.28
**18–29**	0.26 (0.20–0.35)	0.25 (0.18–0.33)	0.27 (0.20–0.36)	0.24 (0.18–0.30)	2.0[-7.9 : 13]	-7.7[-17 : 2.2]	0.175
**30–39**	0.26 (0.19–0.34)	0.24 (0.17–0.31)	0.24 (0.19–0.30)	0.21 (0.15–0.27)	-11[-21 : 0.32]	**-13[-23 : -2.5]**	0.765
**40–49**	0.24 (0.17–0.32)	0.21 (0.15–0.29)	0.21 (0.15–0.28)	0.21 (0.15–0.27)	**-16[-26 : -4.0]**	-1.1[-13 : 12]	0.081
**50–59**	0.23 (0.17–0.31)	0.22 (0.15–0.29)	0.21 (0.14–0.26)	0.21 (0.14–0.28)	**-22[-32 : -11]**	-4.6[-17 : 9.6]	0.043
**60–69**	0.22 (0.15–0.29)	0.23 (0.17–0.32)	0.20 (0.13–0.26)	0.20 (0.15–0.25)	-11[-23 : 2.1]	-8.5[-20 : 4.6]	0.761

Supplementary Table 15: IFNγ (Interferon gamma, IFNG, IFG, IFI, Uniprot:P01579)

Sex and age-stratified median concentrations

AGE GROUP	Biomarker/cohort information		Median Concentration			Age Group (% change in concentration)		
	**N measured**	**Detection Range**	**Males**	**Females**	**Sex difference**	**Males**	**Females**	**Sex × age**
		(% in range)	(pg/ml)	(pg/ml)	F relative to M (%)	change/10 years (%)	change/10 years (%)	Interaction P
**All**	9873	100	5.2 (3.7–7.9)	5.5 (3.9–8.4)	3.6[-6.0 : 14]	**5.3[3.7 : 6.9]**	**5.9[4.4 : 7.5]**	0.555
**18–29**	1994	99.9	4.8 (3.4–6.8)	5.2 (3.6–7.5)	-8.4[-46 : 55]	-4.0[-18 : 12]	2.3[-12 : 19]	0.556
**30–39**	2010	100	5.0 (3.6–7.5)	5.3 (3.8–7.9)	20[-47 : 171]	-0.59[-16 : 17]	-4.5[-19 : 13]	0.736
**40–49**	2011	100	5.0 (3.5–7.2)	5.2 (3.6–7.6)	32[-51 : 253]	8.5[-6.8 : 26]	3.4[-12 : 21]	0.666
**50–59**	1986	99.9	5.6 (4.0–8.6)	5.9 (4.2–9.0)	-36[-81 : 110]	11[-5.0 : 29]	21[4.1 : 42]	0.407
**60–69**	1872	99.9	6.0 (4.1–9.8)	6.6 (4.7–11)	-58[-96 : 301]	0.83[-21 : 29]	18[-9.2 : 52]	0.398

BMI and age-stratified median concentrations

AGE GROUP	Normal BMI		Overweight BMI					Obese BMI				
	**Males**	**Females**	**Males**	**Females**	**Males**	**Females**	**Overweight × sex**	**Males**	**Females**	**Males**	**Females**	**Obese × sex**
	Concentration (pg/ml)	Concentration (pg/ml)	Concentration (pg/ml)	Concentration (pg/ml)	%change	%change	Interaction P	Concentration (pg/ml)	Concentration (pg/ml)	%change	%change	Interaction P
**All**	5.0 (3.5–7.6)	5.4 (3.8–8.0)	5.4 (3.8–8.2)	5.7 (4.1–8.9)	4.1[-0.45 : 8.8]	5.8[-0.37 : 12]	0.232	5.4 (3.9–8.2)	5.7 (4.2–9.1)	5.1[-1.6 : 12]	7.8[-3.4 : 20]	0.077
**18–29**	4.7 (3.4–6.9)	5.1 (3.6–7.4)	4.8 (3.4–6.7)	5.3 (3.6–7.4)	-4.8[-14 : 6.0]	-3.1[-16 : 12]	0.346	5.4 (3.5–6.8)	5.6 (4.0–8.2)	-2.0[-18 : 18]	6.6[-19 : 40]	0.086
**30–39**	4.9 (3.5–7.4)	5.1 (3.6–7.5)	5.1 (3.6–7.6)	5.3 (3.8–8.4)	2.4[-7.7 : 14]	0.79[-12 : 16]	0.416	4.9 (3.7–7.0)	5.4 (4.3–8.7)	-0.64[-14 : 15]	-1.6[-23 : 26]	0.049
**40–49**	4.8 (3.4–7.0)	5.2 (3.5–7.2)	5.0 (3.5–7.3)	5.4 (3.8–7.9)	5.3[-4.3 : 16]	16[2.5 : 32]	0.066	5.3 (3.7–7.0)	5.4 (3.8–7.7)	1.3[-11 : 16]	0.35[-19 : 25]	0.433
**50–59**	5.2 (3.7–8.2)	5.6 (3.9–8.2)	5.7 (4.1–8.7)	6.1 (4.4–9.4)	11[0.59 : 22]	12[-1.1 : 28]	0.793	6.2 (4.1–9.4)	6.3 (4.7–11)	17[1.8 : 34]	38[8.9 : 76]	0.35
**60–69**	5.5 (3.7–9.4)	6.7 (4.5–10)	6.2 (4.4–10)	6.6 (4.8–11)	10[-1.0 : 23]	-3.2[-16 : 12]	0.215	6.3 (4.4–10)	6.3 (4.8–11)	21[2.4 : 43]	-5.4[-27 : 23]	0.146

Smoking and age-stratified median concentrations

AGE GROUP	Non-smokers		Smokers		% Change in Concentration smokers vs non-smokers		
	**Males**	**Females**	**Males**	**Females**	**Males**	**Females**	**Smoking × sex**
	Concentration (pg/ml)	Concentration (pg/ml)	Concentration (pg/ml)	Concentration (pg/ml)	change (%)	change (%)	Interaction P
**All**	5.3 (3.7–8.0)	5.5 (3.9–8.4)	4.8 (3.4–7.2)	5.4 (3.7–8.1)	**-9.6[-15 : -3.9]**	-1.0[-6.8 : 5.1]	0.036
**18–29**	4.8 (3.4–6.8)	5.2 (3.6–7.5)	4.7 (3.4–6.7)	5.2 (3.6–7.6)	-3.4[-15 : 9.8]	7.7[-5.1 : 22]	0.236
**30–39**	5.0 (3.6–7.5)	5.3 (3.8–7.8)	4.5 (3.3–6.9)	5.0 (3.6–7.6)	**-16[-28 : -3.6]**	0.70[-12 : 16]	0.067
**40–49**	5.0 (3.5–7.3)	5.2 (3.7–7.6)	4.5 (3.2–6.1)	5.3 (3.5–7.9)	**-14[-25 : -1.2]**	-3.1[-15 : 10]	0.223
**50–59**	5.7 (4.1–8.8)	5.8 (4.2–9.0)	4.8 (3.4–7.3)	6.7 (4.2–9.3)	**-20[-29 : -8.6]**	4.2[-8.6 : 19]	0.005
**60–69**	6.0 (4.1–9.6)	6.8 (4.7–11)	6.0 (4.1–11)	5.6 (4.2–8.4)	4.9[-9.8 : 22]	**-17[-28 : -4.4]**	0.025

Supplementary Table 16: IL-2 (Interleukin-2, TCGF, Uniprot:P60568)

Sex and age-stratified median concentrations

AGE GROUP	Biomarker/cohort information		Median Concentration			Age Group (% change in concentration)		
	**N measured**	**Detection Range** (% in range)	**Males** (pg/ml)	**Females** (pg/ml)	**Sex difference** F relative to M (%)	**Males** change/10 years (%)	**Females** change/10 years (%)	**Sex × age** Interaction P
**All**	9869	77.3	0.14 (0.075–0.23)	0.14 (0.070–0.23)	-16[-28 : -0.82]	-1.9[-4.3 : 0.58]	0.53[-1.9 : 3.1]	0.168
**18–29**	1993	78.9	0.14 (0.077–0.23)	0.13 (0.069–0.22)	-21[-67 : 92]	10[-15 : 43]	18[-8.3 : 52]	0.709
**30–39**	2009	76.7	0.15 (0.077–0.26)	0.14 (0.075–0.23)	66[-55 : 517]	4.6[-20 : 36]	-12[-33 : 14]	0.359
**40–49**	2011	75.4	0.14 (0.076–0.22)	0.13 (0.065–0.21)	-0.76[-82 : 437]	2.3[-21 : 33]	-0.68[-24 : 30]	0.876
**50–59**	1985	77.2	0.13 (0.065–0.23)	0.14 (0.075–0.23)	-62[-95 : 203]	-7.2[-29 : 22]	12[-14 : 47]	0.332
**60–69**	1871	78.5	0.14 (0.077–0.23)	0.14 (0.071–0.24)	-16[-98 : 3046]	-11[-40 : 32]	-9.1[-40 : 38]	0.943

BMI and age-stratified median concentrations

AGE GROUP	Normal BMI		Overweight BMI					Obese BMI				
	**Males** Concentration (pg/ml)	**Females** Concentration (pg/ml)	**Males** Concentration (pg/ml)	**Females** Concentration (pg/ml)	**Males** %change	**Females** %change	**Overweight × sex** Interaction P	**Males** Concentration (pg/ml)	**Females** Concentration (pg/ml)	**Males** %change	**Females** %change	**Obese × sex** Interaction P
**All**	0.14 (0.076–0.24)	0.14 (0.071–0.23)	0.14 (0.074–0.23)	0.14 (0.070–0.24)	-3.1[-10 : 4.4]	2.4[-7.3 : 13]	0.363	0.14 (0.073–0.22)	0.12 (0.069–0.20)	-12[-21 : -1.2]	-0.74[-17 : 19]	0.192
**18–29**	0.14 (0.079–0.23)	0.14 (0.070–0.23)	0.13 (0.070–0.23)	0.15 (0.074–0.22)	-16[-30 : 0.39]	-6.2[-27 : 21]	0.259	0.16 (0.10–0.23)	0.11 (0.064–0.22)	10[-19 : 51]	-10[-43 : 43]	0.597
**30–39**	0.15 (0.079–0.26)	0.14 (0.075–0.25)	0.15 (0.077–0.25)	0.14 (0.071–0.22)	-1.0[-16 : 17]	-15[-32 : 5.7]	0.319	0.14 (0.071–0.24)	0.13 (0.085–0.19)	-9.5[-28 : 14]	-16[-44 : 26]	0.96
**40–49**	0.14 (0.081–0.22)	0.12 (0.062–0.21)	0.14 (0.075–0.23)	0.14 (0.073–0.24)	-0.46[-16 : 17]	30[5.0 : 62]	0.037	0.13 (0.066–0.22)	0.11 (0.056–0.19)	-17[-34 : 4.5]	-0.31[-32 : 45]	0.358
**50–59**	0.13 (0.063–0.26)	0.14 (0.082–0.23)	0.13 (0.069–0.22)	0.13 (0.066–0.23)	-1.7[-17 : 16]	-10[-28 : 12]	0.693	0.13 (0.053–0.19)	0.13 (0.076–0.21)	-19[-36 : 2.8]	-13[-43 : 33]	0.311
**60–69**	0.14 (0.073–0.24)	0.14 (0.068–0.24)	0.14 (0.077–0.23)	0.14 (0.067–0.24)	-0.94[-17 : 18]	15[-8.3 : 44]	0.932	0.14 (0.076–0.21)	0.14 (0.077–0.22)	-12[-33 : 15]	56[2.1 : 139]	0.173

Smoking and age-stratified median concentrations

AGE GROUP	Non-smokers		Smokers		% Change in Concentration smokers vs non-smokers		
	**Males** Concentration (pg/ml)	**Females** Concentration (pg/ml)	**Males** Concentration (pg/ml)	**Females** Concentration (pg/ml)	**Males** change (%)	**Females** change (%)	**Smoking × sex** Interaction P
**All**	0.14 (0.076–0.24)	0.14 (0.072–0.23)	0.12 (0.066–0.21)	0.12 (0.062–0.20)	**-14[-23 : -5.1]**	**-18[-26 : -9.4]**	0.535
**18–29**	0.14 (0.078–0.23)	0.14 (0.072–0.23)	0.12 (0.076–0.22)	0.12 (0.059–0.19)	-15[-32 : 5.4]	-16[-32 : 3.9]	0.94
**30–39**	0.15 (0.077–0.26)	0.14 (0.077–0.24)	0.13 (0.079–0.19)	0.12 (0.071–0.18)	-18[-35 : 3.4]	**-21[-37 : -0.68]**	0.832
**40–49**	0.14 (0.080–0.22)	0.13 (0.065–0.21)	0.12 (0.053–0.20)	0.11 (0.064–0.18)	-20[-37 : 0.94]	**-20[-36 : -0.26]**	0.997
**50–59**	0.14 (0.066–0.23)	0.14 (0.075–0.23)	0.11 (0.054–0.18)	0.14 (0.074–0.24)	-18[-35 : 3.3]	-1.6[-22 : 24]	0.276
**60–69**	0.14 (0.077–0.23)	0.14 (0.074–0.24)	0.13 (0.067–0.24)	0.11 (0.049–0.21)	-4.4[-25 : 22]	**-28[-43 : -9.7]**	0.09

Supplementary Table 17: IL-4 (Interleukin-4, 4BSF, Uniprot:P05112)

Sex and age-stratified median concentrations

AGE GROUP	Biomarker/cohort information		Median Concentration			Age Group (% change in concentration)		
	N measured	Detection Range	Males	Females	Sex difference	Males	Females	Sex × age
		(% in range)	(pg/ml)	(pg/ml)	F relative to M (%)	change/10 years (%)	change/10 years (%)	Interaction P
**All**	9869	68.1	0.040 (0.020–0.068)	0.039 (0.019–0.065)	6.0[-9.1 : 24]	1.8[-0.58 : 4.3]	-0.49[-2.8 : 1.9]	0.176
**18–29**	1993	66.1	0.039 (0.020–0.063)	0.039 (0.021–0.067)	-35[-70 : 41]	-12[-30 : 10]	7.3[-14 : 34]	0.215
**30–39**	2010	69.1	0.040 (0.019–0.066)	0.037 (0.018–0.060)	-8.9[-74 : 220]	-1.3[-23 : 27]	-1.2[-23 : 27]	0.995
**40–49**	2009	69.4	0.041 (0.021–0.069)	0.039 (0.020–0.065)	47[-70 : 627]	-2.8[-24 : 24]	-12[-32 : 13]	0.561
**50–59**	1985	67.6	0.040 (0.021–0.068)	0.039 (0.020–0.067)	84[-75 : 1233]	3.5[-20 : 34]	-7.6[-28 : 19]	0.536
**60–69**	1872	68.4	0.042 (0.020–0.075)	0.039 (0.018–0.070)	-15[-98 : 3179]	-4.2[-36 : 42]	-2.9[-36 : 48]	0.964

BMI and age-stratified median concentrations

AGE GROUP	Normal BMI		Overweight BMI					Obese BMI				
	Males	Females	Males	Females	Males	Females	Overweight × sex	Males	Females	Males	Females	Obese × sex
	Concentration (pg/ml)	Concentration (pg/ml)	Concentration (pg/ml)	Concentration (pg/ml)	%change	%change	Interaction P	Concentration (pg/ml)	Concentration (pg/ml)	%change	%change	Interaction P
**All**	0.039 (0.020–0.066)	0.038 (0.019–0.064)	0.040 (0.020–0.070)	0.041 (0.021–0.067)	0.74[-6.1 : 8.1]	1.2[-7.9 : 11]	0.31	0.047 (0.024–0.073)	0.039 (0.019–0.066)	14[3.0 : 27]	-9.6[-24 : 7.5]	0.107
**18–29**	0.040 (0.021–0.065)	0.039 (0.020–0.068)	0.033 (0.018–0.059)	0.045 (0.022–0.069)	-11[-24 : 4.0]	3.1[-17 : 29]	0.204	0.042 (0.028–0.066)	0.040 (0.024–0.066)	14[-13 : 50]	6.5[-29 : 59]	0.703
**30–39**	0.039 (0.018–0.064)	0.036 (0.017–0.060)	0.041 (0.019–0.065)	0.040 (0.020–0.064)	7.9[-7.9 : 26]	8.5[-12 : 34]	0.916	0.047 (0.023–0.070)	0.036 (0.017–0.054)	23[-1.6 : 53]	-8.7[-38 : 34]	0.073
**40–49**	0.039 (0.020–0.065)	0.039 (0.021–0.061)	0.041 (0.022–0.072)	0.041 (0.018–0.064)	2.4[-12 : 20]	-14[-30 : 5.8]	0.592	0.046 (0.024–0.074)	0.039 (0.018–0.071)	7.2[-14 : 33]	-29[-50 : 1.8]	0.365
**50–59**	0.039 (0.020–0.062)	0.038 (0.019–0.068)	0.041 (0.022–0.075)	0.040 (0.021–0.064)	9.6[-6.5 : 29]	11[-10 : 38]	0.938	0.044 (0.020–0.069)	0.042 (0.021–0.068)	11[-12 : 39]	7.8[-28 : 61]	0.62
**60–69**	0.043 (0.020–0.072)	0.036 (0.016–0.063)	0.040 (0.019–0.075)	0.043 (0.021–0.074)	-11[-25 : 5.7]	1.1[-20 : 27]	0.008	0.054 (0.030–0.086)	0.039 (0.019–0.084)	17[-11 : 53]	-19[-47 : 24]	0.586

Smoking and age-stratified median concentrations

AGE GROUP	Non-smokers		Smokers		% Change in Concentration smokers vs non-smokers		
	Males	Females	Males	Females	Males	Females	Smoking × sex
	Concentration (pg/ml)	Concentration (pg/ml)	Concentration (pg/ml)	Concentration (pg/ml)	change (%)	change (%)	Interaction P
**All**	0.040 (0.020–0.068)	0.039 (0.019–0.066)	0.042 (0.022–0.071)	0.039 (0.018–0.065)	5.0[-4.7 : 16]	0.65[-8.4 : 11]	0.542
**18–29**	0.039 (0.020–0.064)	0.040 (0.021–0.068)	0.036 (0.021–0.064)	0.039 (0.017–0.064)	-2.2[-19 : 18]	-12[-27 : 6.2]	0.44
**30–39**	0.040 (0.019–0.066)	0.037 (0.018–0.061)	0.038 (0.020–0.058)	0.039 (0.019–0.057)	-5.1[-24 : 18]	-2.0[-21 : 22]	0.836
**40–49**	0.040 (0.020–0.067)	0.039 (0.021–0.063)	0.051 (0.031–0.085)	0.039 (0.018–0.070)	**33[6.9 : 66]**	11[-10 : 36]	0.228
**50–59**	0.040 (0.021–0.070)	0.040 (0.020–0.068)	0.039 (0.020–0.062)	0.039 (0.018–0.064)	-11[-29 : 9.9]	-2.5[-22 : 21]	0.542
**60–69**	0.041 (0.020–0.072)	0.039 (0.018–0.069)	0.053 (0.028–0.089)	0.039 (0.018–0.074)	**31[2.6 : 67]**	22[-3.5 : 54]	0.677

Supplementary Table 18: IL-5 (Interleukin-5, Uniprot:P05113)

Sex and age-stratified median concentrations

AGE GROUP	Biomarker/cohort information		Median Concentration			Age Group (% change in concentration)		
	N measured	Detection Range (% in range)	Males (pg/ml)	Females (pg/ml)	Sex difference F relative to M (%)	Males change/10 years (%)	Females change/10 years (%)	Sex × age Interaction P
**All**	9871	99.8	0.53 (0.38–0.77)	0.47 (0.33–0.65)	2.5[-5.6 : 11]	**8.0[6.6 : 9.3]**	**3.7[2.4 : 5.0]**	**1.01E-05**
**18–29**	1994	99.7	0.45 (0.31–0.66)	0.44 (0.31–0.60)	-28[-55 : 17]	1.8[-11 : 17]	14[-0.32 : 31]	0.237
**30–39**	2010	99.6	0.51 (0.37–0.74)	0.47 (0.33–0.66)	-14[-57 : 71]	13[-1.7 : 30]	13[-1.6 : 30]	0.996
**40–49**	2011	99.8	0.53 (0.38–0.76)	0.46 (0.33–0.64)	81[-20 : 308]	16[2.6 : 32]	-1.6[-14 : 12]	0.07
**50–59**	1985	99.8	0.57 (0.41–0.80)	0.47 (0.35–0.65)	10[-58 : 191]	16[2.6 : 32]	9.9[-3.0 : 24]	0.532
**60–69**	1871	99.9	0.62 (0.46–0.88)	0.50 (0.36–0.72)	30[-79 : 724]	17[-4.2 : 43]	8.7[-12 : 34]	0.616

BMI and age-stratified median concentrations

AGE GROUP	Normal BMI		Overweight BMI					Obese BMI				
	Males Concentration (pg/ml)	Females Concentration (pg/ml)	Males Concentration (pg/ml)	Females Concentration (pg/ml)	Males %change	Females %change	Overweight × sex Interaction P	Males Concentration (pg/ml)	Females Concentration (pg/ml)	Males %change	Females %change	Obese × sex Interaction P
**All**	0.53 (0.38–0.77)	0.46 (0.33–0.64)	0.54 (0.39–0.77)	0.48 (0.34–0.67)	0.10[-3.6 : 3.9]	-1.3[-6.2 : 3.8]	0.758	0.56 (0.39–0.78)	0.47 (0.35–0.67)	-0.38[-5.8 : 5.3]	-2.0[-11 : 7.6]	0.323
**18–29**	0.45 (0.31–0.67)	0.43 (0.30–0.61)	0.48 (0.31–0.65)	0.46 (0.33–0.59)	0.97[-8.6 : 12]	-3.8[-16 : 10]	0.954	0.44 (0.35–0.65)	0.46 (0.35–0.66)	-2.6[-18 : 15]	-7.4[-28 : 19]	0.549
**30–39**	0.53 (0.37–0.75)	0.46 (0.32–0.64)	0.50 (0.37–0.69)	0.47 (0.33–0.67)	-5.1[-13 : 3.5]	3.6[-7.7 : 16]	0.136	0.54 (0.41–0.81)	0.47 (0.33–0.74)	4.9[-7.0 : 18]	3.8[-16 : 28]	0.867
**40–49**	0.53 (0.40–0.76)	0.47 (0.34–0.65)	0.53 (0.37–0.78)	0.43 (0.32–0.62)	-3.3[-11 : 4.8]	-9.1[-18 : 0.93]	0.639	0.53 (0.38–0.71)	0.46 (0.36–0.61)	-5.2[-15 : 5.7]	-6.5[-22 : 12]	0.337
**50–59**	0.57 (0.42–0.79)	0.47 (0.35–0.64)	0.57 (0.41–0.80)	0.47 (0.35–0.67)	1.4[-6.1 : 9.6]	7.2[-3.4 : 19]	0.336	0.61 (0.42–0.83)	0.46 (0.35–0.64)	0.75[-9.9 : 13]	0.94[-17 : 23]	0.986
**60–69**	0.63 (0.44–0.94)	0.49 (0.35–0.70)	0.62 (0.47–0.87)	0.52 (0.35–0.73)	0.27[-8.1 : 9.4]	3.6[-7.7 : 16]	0.766	0.60 (0.44–0.80)	0.53 (0.38–0.75)	-8.2[-20 : 5.2]	6.9[-14 : 33]	0.209

Smoking and age-stratified median concentrations

AGE GROUP	Non-smokers		Smokers		% Change in Concentration smokers vs non-smokers		
	Males Concentration (pg/ml)	Females Concentration (pg/ml)	Males Concentration (pg/ml)	Females Concentration (pg/ml)	Males change (%)	Females change (%)	Smoking × sex Interaction P
**All**	0.53 (0.38–0.77)	0.47 (0.33–0.65)	0.57 (0.41–0.81)	0.48 (0.33–0.66)	**8.9[3.4 : 15]**	2.9[-2.2 : 8.2]	0.123
**18–29**	0.44 (0.30–0.64)	0.43 (0.31–0.60)	0.55 (0.39–0.81)	0.45 (0.31–0.62)	**31[16 : 47]**	2.8[-8.6 : 16]	0.004
**30–39**	0.50 (0.37–0.73)	0.46 (0.32–0.66)	0.56 (0.39–0.75)	0.49 (0.36–0.66)	3.2[-8.5 : 16]	8.3[-3.7 : 22]	0.573
**40–49**	0.53 (0.38–0.76)	0.45 (0.33–0.62)	0.54 (0.42–0.81)	0.50 (0.34–0.72)	11[-0.80 : 24]	3.6[-7.0 : 15]	0.377
**50–59**	0.57 (0.41–0.80)	0.47 (0.35–0.65)	0.60 (0.45–0.84)	0.49 (0.33–0.65)	5.4[-5.1 : 17]	3.2[-7.3 : 15]	0.778
**60–69**	0.63 (0.46–0.89)	0.51 (0.36–0.74)	0.61 (0.42–0.83)	0.47 (0.32–0.66)	-5.1[-16 : 7.4]	-6.2[-17 : 5.6]	0.89

Supplementary Table 19: IL-9 (Interleukin-9, HP40, Uniprot:P15248)

Sex and age-stratified median concentrations

AGE GROUP	Biomarker/cohort information		Median Concentration			Age Group (% change in concentration)		
	N measured	Detection Range	Males	Females	Sex difference	Males	Females	Sex × age
		(% in range)	(pg/ml)	(pg/ml)	F relative to M (%)	change/10 years (%)	change/10 years (%)	Interaction P
**All**	9732	80.4	0.40 (0.21–0.69)	0.31 (0.15–0.53)	**-37[-46 : -26]**	**10.[7.4 : 13]**	**14[11 : 16]**	0.059
**18–29**	1972	74.4	0.35 (0.18–0.59)	0.25 (0.12–0.43)	-37[-73 : 48]	7.4[-16 : 37]	13[-12 : 44]	0.783
**30–39**	1982	77.9	0.38 (0.20–0.63)	0.27 (0.13–0.48)	18[-67 : 321]	19[-8.1 : 54]	1.6[-21 : 31]	0.395
**40–49**	1979	79	0.40 (0.20–0.64)	0.29 (0.14–0.47)	-53[-91 : 141]	8.2[-16 : 39]	18[-8.7 : 54]	0.624
**50–59**	1960	83.8	0.44 (0.22–0.78)	0.35 (0.16–0.60)	38[-81 : 894]	26[-2.1 : 63]	14[-12 : 47]	0.566
**60–69**	1839	87.3	0.47 (0.27–0.82)	0.38 (0.20–0.72)	-44[-98 : 1702]	16[-20 : 69]	22[-18 : 81]	0.863

BMI and age-stratified median concentrations

AGE GROUP	Normal BMI		Overweight BMI					Obese BMI				
	Males	Females	Males	Females	Males	Females	Overweight × sex	Males	Females	Males	Females	Obese × sex
	Concentration (pg/ml)	Concentration (pg/ml)	Concentration (pg/ml)	Concentration (pg/ml)	%change	%change	Interaction P	Concentration (pg/ml)	Concentration (pg/ml)	%change	%change	Interaction P
**All**	0.38 (0.20–0.67)	0.30 (0.14–0.52)	0.43 (0.23–0.71)	0.32 (0.16–0.55)	2.3[-4.8 : 9.9]	7.5[-2.4 : 18]	0.362	0.39 (0.20–0.67)	0.30 (0.14–0.53)	-3.8[-14 : 6.9]	0.57[-16 : 20]	0.572
**18–29**	0.34 (0.18–0.55)	0.25 (0.11–0.42)	0.38 (0.18–0.63)	0.28 (0.13–0.45)	11[-6.6 : 33]	4.5[-18 : 33]	0.917	0.39 (0.17–0.71)	0.31 (0.099–0.48)	3.2[-23 : 39]	-5.0[-39 : 47]	0.654
**30–39**	0.38 (0.20–0.65)	0.26 (0.12–0.48)	0.39 (0.21–0.63)	0.27 (0.13–0.43)	-0.94[-16 : 17]	2.5[-17 : 27]	0.756	0.35 (0.19–0.55)	0.29 (0.14–0.53)	-2.1[-22 : 23]	23[-17 : 81]	0.136
**40–49**	0.39 (0.20–0.65)	0.27 (0.13–0.45)	0.41 (0.21–0.69)	0.30 (0.15–0.49)	3.9[-12 : 22]	24[0.41 : 53]	0.113	0.39 (0.19–0.61)	0.30 (0.15–0.50)	-4.7[-23 : 19]	20[-17 : 73]	0.082
**50–59**	0.39 (0.20–0.77)	0.35 (0.17–0.61)	0.48 (0.26–0.81)	0.35 (0.17–0.60)	12[-4.1 : 32]	13[-9.1 : 39]	0.24	0.40 (0.20–0.71)	0.32 (0.13–0.55)	-3.1[-23 : 22]	4.0[-30 : 55]	0.134
**60–69**	0.48 (0.25–0.88)	0.38 (0.21–0.73)	0.48 (0.28–0.81)	0.41 (0.21–0.73)	-7.5[-22 : 9.0]	-1.0[-20 : 23]	0.54	0.42 (0.29–0.77)	0.31 (0.16–0.56)	4.7[-19 : 36]	-27[-51 : 10]	0.053

Smoking and age-stratified median concentrations

AGE GROUP	Non-smokers		Smokers		% Change in Concentration smokers vs non-smokers		
	Males	Females	Males	Females	Males	Females	Smoking × sex
	Concentration (pg/ml)	Concentration (pg/ml)	Concentration (pg/ml)	Concentration (pg/ml)	change (%)	change (%)	Interaction P
**All**	0.41 (0.22–0.70)	0.31 (0.15–0.54)	0.37 (0.20–0.63)	0.28 (0.14–0.46)	**-11[-19 : -1.7]**	**-9.6[-18 : -0.47]**	0.828
**18–29**	0.35 (0.17–0.58)	0.26 (0.12–0.44)	0.37 (0.23–0.60)	0.26 (0.12–0.42)	3.7[-16 : 28]	3.8[-15 : 28]	0.992
**30–39**	0.39 (0.21–0.66)	0.29 (0.13–0.49)	0.33 (0.15–0.55)	0.23 (0.10–0.41)	**-32[-46 : -15]**	-18[-34 : 2.0]	0.243
**40–49**	0.40 (0.20–0.64)	0.28 (0.14–0.47)	0.42 (0.20–0.66)	0.31 (0.17–0.46)	-4.1[-24 : 20]	13[-8.7 : 40]	0.302
**50–59**	0.44 (0.23–0.80)	0.35 (0.17–0.60)	0.40 (0.19–0.67)	0.26 (0.14–0.58)	-15[-31 : 5.4]	-19[-35 : 1.4]	0.787
**60–69**	0.48 (0.28–0.83)	0.39 (0.20–0.74)	0.43 (0.24–0.74)	0.33 (0.19–0.55)	-9.7[-28 : 14]	**-27[-42 : -8.7]**	0.195

Supplementary Table 20: IL-10 (Interleukin-10, CSIF, GVHDS, Uniprot:P22301)

Sex and age-stratified median concentrations

AGE GROUP	Biomarker/cohort information		Median Concentration			Age Group (% change in concentration)		
	**N measured**	**Detection Range**	**Males**	**Females**	**Sex difference**	**Males**	**Females**	**Sex × age**
		(% in range)	(pg/ml)	(pg/ml)	F relative to M (%)	change/10 years (%)	change/10 years (%)	Interaction P
**All**	9873	98.5	0.26 (0.18–0.40)	0.25 (0.17–0.38)	-9.3[-18 : 0.76]	**-3.3[-4.9 : -1.8]**	-2.3[-3.9 : -0.72]	0.354
**18–29**	1994	99.1	0.28 (0.20–0.43)	0.27 (0.19–0.41)	-17[-50 : 38]	-11[-23 : 3.0]	-5.6[-18 : 9.2]	0.563
**30–39**	2010	98.4	0.27 (0.18–0.40)	0.25 (0.16–0.37)	-22[-67 : 85]	-14[-28 : 1.9]	-10[-25 : 6.6]	0.72
**40–49**	2011	97.6	0.26 (0.17–0.38)	0.25 (0.16–0.36)	-23[-75 : 139]	-0.66[-17 : 18]	3.5[-14 : 24]	0.748
**50–59**	1986	98.5	0.25 (0.16–0.38)	0.24 (0.16–0.36)	27[-70 : 432]	9.8[-8.7 : 32]	3.8[-14 : 25]	0.672
**60–69**	1872	99	0.27 (0.18–0.39)	0.26 (0.17–0.40)	-17[-92 : 744]	11[-14 : 43]	14[-12 : 49]	0.872

BMI and age-stratified median concentrations

AGE GROUP	Normal BMI		Overweight BMI					Obese BMI				
	**Males**	**Females**	**Males**	**Females**	**Males**	**Females**	**Overweight × sex**	**Males**	**Females**	**Males**	**Females**	**Obese × sex**
	Concentration (pg/ml)	Concentration (pg/ml)	Concentration (pg/ml)	Concentration (pg/ml)	%change	%change	Interaction P	Concentration (pg/ml)	Concentration (pg/ml)	%change	%change	Interaction P
**All**	0.27 (0.18–0.39)	0.26 (0.17–0.38)	0.26 (0.17–0.39)	0.25 (0.16–0.38)	-2.1[-6.7 : 2.7]	-3.5[-9.6 : 3.0]	0.234	0.27 (0.18–0.40)	0.25 (0.16–0.38)	0.89[-6.0 : 8.3]	4.6[-7.1 : 18]	0.526
**18–29**	0.28 (0.20–0.42)	0.27 (0.19–0.40)	0.29 (0.20–0.44)	0.27 (0.18–0.42)	0.71[-9.2 : 12]	-2.3[-15 : 13]	0.659	0.31 (0.23–0.41)	0.29 (0.21–0.45)	6.0[-11 : 27]	18[-9.4 : 53]	0.431
**30–39**	0.26 (0.18–0.39)	0.26 (0.17–0.38)	0.27 (0.19–0.42)	0.24 (0.15–0.35)	6.2[-4.8 : 18]	-12[-24 : 1.8]	0.04	0.27 (0.17–0.47)	0.26 (0.17–0.37)	9.6[-5.8 : 27]	-1.3[-24 : 28]	0.604
**40–49**	0.25 (0.16–0.39)	0.24 (0.17–0.35)	0.26 (0.17–0.36)	0.25 (0.15–0.37)	-2.3[-12 : 9.1]	3.9[-10 : 20]	0.923	0.26 (0.18–0.38)	0.25 (0.16–0.39)	3.0[-12 : 20]	14[-12 : 47]	0.496
**50–59**	0.26 (0.17–0.39)	0.24 (0.16–0.37)	0.24 (0.15–0.37)	0.24 (0.16–0.36)	-7.9[-18 : 3.4]	1.8[-13 : 19]	0.602	0.25 (0.17–0.37)	0.22 (0.13–0.36)	-6.3[-21 : 11]	2.4[-23 : 37]	0.725
**60–69**	0.28 (0.19–0.38)	0.26 (0.16–0.40)	0.27 (0.17–0.38)	0.26 (0.17–0.40)	-2.8[-13 : 8.5]	-13[-25 : 0.65]	0.341	0.27 (0.17–0.39)	0.26 (0.17–0.38)	-3.7[-19 : 14]	-15[-35 : 12]	0.731

Smoking and age-stratified median concentrations

AGE GROUP	Non-smokers		Smokers		% Change in Concentration smokers vs non-smokers		
	**Males**	**Females**	**Males**	**Females**	**Males**	**Females**	**Smoking × sex**
	Concentration (pg/ml)	Concentration (pg/ml)	Concentration (pg/ml)	Concentration (pg/ml)	change (%)	change (%)	Interaction P
**All**	0.26 (0.18–0.40)	0.26 (0.17–0.38)	0.26 (0.17–0.39)	0.24 (0.16–0.38)	-4.9[-11 : 1.6]	-5.8[-12 : 0.47]	0.84
**18–29**	0.29 (0.20–0.43)	0.27 (0.19–0.41)	0.27 (0.20–0.43)	0.28 (0.19–0.39)	0.020[-12 : 13]	-3.7[-15 : 8.9]	0.672
**30–39**	0.26 (0.19–0.40)	0.25 (0.17–0.38)	0.27 (0.18–0.39)	0.24 (0.14–0.36)	-3.1[-17 : 13]	**-15[-27 : -2.1]**	0.203
**40–49**	0.26 (0.17–0.39)	0.25 (0.17–0.36)	0.23 (0.15–0.37)	0.22 (0.14–0.35)	-7.6[-21 : 7.9]	**-14[-26 : -0.28]**	0.516
**50–59**	0.25 (0.16–0.38)	0.24 (0.16–0.36)	0.25 (0.15–0.34)	0.22 (0.16–0.34)	-4.5[-18 : 12]	-4.9[-19 : 11]	0.97
**60–69**	0.27 (0.18–0.38)	0.26 (0.17–0.39)	0.26 (0.14–0.38)	0.26 (0.16–0.42)	-8.7[-22 : 6.6]	10[-4.8 : 28]	0.081

Supplementary Table 21: IL-13 (Interleukin-13, P600, Uniprot:P35225)

Sex and age-stratified median concentrations

AGE GROUP	Biomarker/cohort information		Median Concentration			Age Group (% change in concentration)		
	N measured	Detection Range (% in range)	Males (pg/ml)	Females (pg/ml)	Sex difference F relative to M (%)	Males change/10 years (%)	Females change/10 years (%)	Sex × age Interaction P
**All**	9872	54.8	0.54 (0.30–0.85)	0.54 (0.29–0.84)	-1.7[-14 : 13]	1.3[-0.86 : 3.5]	1.4[-0.77 : 3.5]	0.96
**18–29**	1994	54.9	0.53 (0.28–0.82)	0.54 (0.28–0.80)	-16[-61 : 80]	6.1[-15 : 33]	12[-10 : 40]	0.728
**30–39**	2010	55.8	0.55 (0.30–0.89)	0.55 (0.31–0.88)	87[-38 : 469]	30[3.5 : 62]	9.1[-13 : 37]	0.288
**40–49**	2010	55.7	0.55 (0.30–0.87)	0.56 (0.31–0.87)	15[-73 : 391]	-1.4[-21 : 23]	-5.0[-25 : 20]	0.822
**50–59**	1986	54.3	0.54 (0.31–0.83)	0.53 (0.29–0.83)	180[-52 : 1520]	10[-12 : 39]	-8.9[-27 : 14]	0.238
**60–69**	1872	53.2	0.55 (0.30–0.85)	0.54 (0.27–0.84)	51[-92 : 2710]	1.2[-26 : 39]	-5.1[-32 : 33]	0.785

BMI and age-stratified median concentrations

AGE GROUP	Normal BMI		Overweight BMI					Obese BMI				
	Males Concentration (pg/ml)	Females Concentration (pg/ml)	Males Concentration (pg/ml)	Females Concentration (pg/ml)	Males %change	Females %change	Overweight × sex Interaction P	Males Concentration (pg/ml)	Females Concentration (pg/ml)	Males %change	Females %change	Obese × sex Interaction P
**All**	0.54 (0.29–0.84)	0.54 (0.29–0.83)	0.54 (0.30–0.86)	0.54 (0.31–0.85)	0.12[-6.0 : 6.6]	2.2[-6.2 : 11]	0.307	0.56 (0.31–0.88)	0.56 (0.30–0.85)	4.2[-5.1 : 14]	-0.37[-15 : 16]	0.75
**18–29**	0.54 (0.27–0.83)	0.53 (0.27–0.79)	0.50 (0.29–0.79)	0.52 (0.26–0.83)	-6.9[-21 : 9.2]	-11[-29 : 11]	0.571	0.59 (0.33–0.93)	0.53 (0.28–0.77)	12[-15 : 47]	-24[-49 : 14]	0.549
**30–39**	0.53 (0.29–0.86)	0.55 (0.30–0.88)	0.55 (0.31–0.87)	0.54 (0.28–0.87)	4.6[-9.1 : 20]	4.7[-13 : 26]	0.981	0.58 (0.35–0.96)	0.61 (0.37–0.88)	12[-8.1 : 36]	16[-17 : 64]	0.815
**40–49**	0.57 (0.31–0.87)	0.55 (0.32–0.80)	0.54 (0.31–0.86)	0.60 (0.31–0.93)	-0.39[-14 : 15]	13[-6.6 : 36]	0.611	0.53 (0.30–0.82)	0.57 (0.32–0.91)	-6.1[-23 : 14]	29[-6.6 : 79]	0.276
**50–59**	0.53 (0.31–0.82)	0.51 (0.28–0.83)	0.53 (0.30–0.84)	0.54 (0.34–0.81)	-4.9[-17 : 9.5]	7.0[-11 : 29]	0.111	0.57 (0.34–0.90)	0.58 (0.29–0.88)	-0.31[-19 : 22]	-0.65[-30 : 42]	0.491
**60–69**	0.55 (0.33–0.84)	0.54 (0.28–0.87)	0.54 (0.28–0.87)	0.52 (0.30–0.82)	0.45[-13 : 15]	-1.4[-18 : 18]	0.804	0.49 (0.30–0.79)	0.54 (0.23–0.75)	2.1[-18 : 27]	-17[-41 : 17]	0.483

Smoking and age-stratified median concentrations

AGE GROUP	Non-smokers		Smokers		% Change in Concentration smokers vs non-smokers		
	Males Concentration (pg/ml)	Females Concentration (pg/ml)	Males Concentration (pg/ml)	Females Concentration (pg/ml)	Males change (%)	Females change (%)	Smoking × sex Interaction P
**All**	0.54 (0.29–0.85)	0.54 (0.29–0.84)	0.54 (0.33–0.86)	0.53 (0.29–0.83)	5.4[-3.3 : 15]	-1.9[-9.8 : 6.8]	0.246
**18–29**	0.54 (0.26–0.82)	0.53 (0.27–0.80)	0.55 (0.33–0.85)	0.52 (0.30–0.78)	18[-2.0 : 43]	-1.8[-19 : 18]	0.166
**30–39**	0.55 (0.31–0.87)	0.56 (0.31–0.89)	0.54 (0.27–0.93)	0.54 (0.33–0.79)	-2.5[-20 : 19]	-8.4[-24 : 11]	0.652
**40–49**	0.55 (0.30–0.87)	0.56 (0.31–0.87)	0.56 (0.38–0.84)	0.58 (0.35–0.86)	6.0[-13 : 30]	6.9[-12 : 29]	0.951
**50–59**	0.54 (0.31–0.85)	0.54 (0.31–0.83)	0.48 (0.33–0.77)	0.53 (0.26–0.86)	-4.1[-21 : 16]	-0.74[-18 : 21]	0.802
**60–69**	0.54 (0.29–0.84)	0.54 (0.28–0.84)	0.62 (0.36–0.89)	0.49 (0.27–0.85)	13[-7.4 : 37]	-6.0[-22 : 13]	0.19

Supplementary Table 22: IL-16 (Interleukin-16, LCF, NPRprIL-16, Uniprot:Q14005)

Sex and age-stratified median concentrations

AGE GROUP	Biomarker/cohort information		Median Concentration			Age Group (% change in concentration)		
	**N measured**	**Detection Range** (% in range)	**Males** (pg/ml)	**Females** (pg/ml)	**Sex difference** F relative to M (%)	**Males** change/10 years (%)	**Females** change/10 years (%)	**Sex × age** Interaction P
**All**	9836	100	1015 (706–1537)	933 (633–1338)	**-18[-24 : -12]**	**-3.1[-4.2 : -1.9]**	-1.2[-2.4 : -0.070]	0.027
**18–29**	1991	99.9	1103 (745–1681)	955 (627–1413)	-17[-46 : 27]	5.7[-6.8 : 20]	7.2[-5.3 : 21]	0.871
**30–39**	1999	99.9	1043 (729–1537)	926 (634–1314)	-0.24[-46 : 84]	-5.3[-16 : 7.2]	-9.1[-20 : 2.8]	0.646
**40–49**	2003	100	980 (699–1515)	930 (626–1303)	39[-39 : 217]	-8.5[-19 : 3.8]	-17[-27 : -5.4]	0.296
**50–59**	1979	99.9	997 (713–1493)	937 (641–1331)	0.010[-62 : 162]	3.3[-8.7 : 17]	1.7[-10 : 15]	0.857
**60–69**	1864	99.9	968 (681–1448)	920 (635–1331)	-44[-90 : 226]	-2.4[-19 : 18]	5.6[-14 : 29]	0.58

BMI and age-stratified median concentrations

AGE GROUP	Normal BMI		Overweight BMI					Obese BMI				
	**Males** Concentration (pg/ml)	**Females** Concentration (pg/ml)	**Males** Concentration (pg/ml)	**Females** Concentration (pg/ml)	**Males** %change	**Females** %change	**Overweight × sex** Interaction P	**Males** Concentration (pg/ml)	**Females** Concentration (pg/ml)	**Males** %change	**Females** %change	**Obese × sex** Interaction P
**All**	1002 (703–1514)	945 (623–1332)	1022 (719–1543)	951 (655–1393)	1.6[-1.9 : 5.3]	1.8[-2.9 : 6.8]	0.602	1058 (740–1684)	934 (656–1308)	6.3[0.84 : 12]	-3.2[-11 : 5.7]	0.077
**18–29**	1089 (745–1678)	959 (621–1405)	1162 (771–1722)	967 (671–1577)	-0.51[-9.0 : 8.7]	2.4[-9.5 : 16]	0.21	1110 (814–1927)	952 (686–1214)	11[-4.4 : 30]	-13[-30 : 8.9]	0.239
**30–39**	1042 (720–1541)	926 (618–1307)	1013 (733–1447)	954 (675–1324)	0.32[-7.2 : 8.4]	4.6[-5.6 : 16]	0.532	1162 (766–1870)	942 (665–1344)	11[-0.66 : 23]	0.88[-16 : 22]	0.16
**40–49**	969 (702–1460)	946 (644–1293)	1030 (730–1599)	958 (633–1383)	2.6[-5.3 : 11]	-0.070[-10 : 11]	0.576	952 (681–1505)	893 (591–1268)	-1.2[-12 : 10]	-2.3[-19 : 17]	0.715
**50–59**	965 (701–1465)	969 (650–1363)	1014 (724–1493)	911 (647–1363)	2.5[-5.1 : 11]	-4.7[-14 : 5.8]	0.329	1081 (765–1612)	874 (655–1153)	13[0.71 : 26]	-14[-29 : 4.7]	0.005
**60–69**	936 (664–1335)	896 (603–1289)	970 (682–1504)	948 (669–1381)	7.1[-1.4 : 16]	4.1[-6.7 : 16]	0.967	1056 (741–1546)	1120 (708–1598)	8.3[-4.9 : 23]	11[-10 : 36]	0.302

Smoking and age-stratified median concentrations

AGE GROUP	Non-smokers		Smokers		% Change in Concentration smokers vs non-smokers		
	**Males** Concentration (pg/ml)	**Females** Concentration (pg/ml)	**Males** Concentration (pg/ml)	**Females** Concentration (pg/ml)	**Males** change (%)	**Females** change (%)	**Smoking × sex** Interaction P
**All**	1010 (709–1524)	930 (632–1330)	1080 (739–1668)	1001 (705–1449)	**8.4[3.2 : 14]**	**11[5.9 : 17]**	0.48
**18–29**	1107 (750–1690)	955 (627–1439)	1147 (766–1806)	978 (695–1371)	6.7[-4.0 : 19]	7.4[-3.3 : 19]	0.935
**30–39**	1036 (722–1504)	926 (630–1321)	1061 (738–1585)	976 (728–1337)	10.[-1.2 : 22]	9.6[-1.3 : 22]	0.961
**40–49**	979 (698–1501)	919 (619–1269)	1132 (767–1732)	1063 (725–1507)	**15[2.4 : 29]**	**18[5.5 : 31]**	0.764
**50–59**	983 (721–1468)	921 (639–1305)	1097 (706–1629)	1030 (713–1561)	6.6[-4.0 : 18]	**19[6.9 : 32]**	0.15
**60–69**	971 (683–1442)	925 (641–1330)	948 (700–1591)	975 (622–1379)	5.4[-6.3 : 18]	4.2[-6.9 : 17]	0.89

Supplementary Table 23: IL-17A (Interleukin-17A, CTLA8, Uniprot:Q16552)

Sex and age-stratified median concentrations

AGE GROUP	Biomarker/cohort information		Median Concentration			Age Group (% change in concentration)		
	**N measured**	**Detection Range** (% in range)	**Males** (pg/ml)	**Females** (pg/ml)	**Sex difference** F relative to M (%)	**Males** change/10 years (%)	**Females** change/10 years (%)	**Sex × age** Interaction P
**All**	9872	99	2.2 (1.5–3.0)	2.4 (1.7–3.3)	10[1.4 : 20]	**-5.4[-6.7 : -4.2]**	**-4.7[-5.9 : -3.4]**	0.392
**18–29**	1994	99.5	2.3 (1.7–3.2)	2.6 (1.9–3.7)	-23[-47 : 14]	**-17[-26 : -7.3]**	-2.8[-13 : 8.6]	0.044
**30–39**	2010	99.3	2.3 (1.6–3.1)	2.5 (1.8–3.6)	17[-42 : 138]	-12[-24 : 1.2]	-13[-25 : 0.080]	0.913
**40–49**	2011	98.9	2.1 (1.4–2.9)	2.3 (1.6–3.1)	-12[-62 : 106]	-15[-26 : -3.5]	-11[-22 : 2.1]	0.593
**50–59**	1985	98.8	2.1 (1.4–2.8)	2.3 (1.6–3.2)	-42[-81 : 80]	-10[-22 : 3.9]	2.1[-12 : 18]	0.223
**60–69**	1872	98.7	2.1 (1.4–3.0)	2.3 (1.6–3.3)	193[-65 : 2380]	20[-4.6 : 52]	4.0[-19 : 33]	0.398

BMI and age-stratified median concentrations

AGE GROUP	Normal BMI		Overweight BMI					Obese BMI				
	**Males** Concentration (pg/ml)	**Females** Concentration (pg/ml)	**Males** Concentration (pg/ml)	**Females** Concentration (pg/ml)	**Males** %change	**Females** %change	**Overweight × sex** Interaction P	**Males** Concentration (pg/ml)	**Females** Concentration (pg/ml)	**Males** %change	**Females** %change	**Obese × sex** Interaction P
**All**	2.1 (1.5–2.9)	2.3 (1.6–3.2)	2.2 (1.5–3.1)	2.4 (1.8–3.4)	**6.1[2.0 : 10]**	4.8[-0.52 : 10]	0.144	2.4 (1.6–3.3)	2.7 (1.9–3.7)	**12[6.0 : 19]**	2.6[-6.8 : 13]	0.321
**18–29**	2.3 (1.7–3.1)	2.6 (1.9–3.6)	2.4 (1.8–3.4)	2.7 (1.9–3.6)	10[1.7 : 19]	-4.0[-14 : 7.1]	0.254	2.5 (1.9–3.4)	3.1 (2.0–4.1)	12[-2.3 : 28]	-6.8[-24 : 14]	0.808
**30–39**	2.3 (1.6–3.0)	2.4 (1.7–3.3)	2.2 (1.7–3.1)	2.5 (1.8–3.6)	3.0[-5.8 : 13]	-1.3[-12 : 11]	0.927	2.6 (1.7–3.4)	2.8 (2.2–4.1)	16[2.8 : 32]	6.0[-15 : 32]	0.7
**40–49**	1.9 (1.3–2.6)	2.1 (1.5–2.9)	2.1 (1.4–2.9)	2.4 (1.6–3.2)	4.9[-3.4 : 14]	12[0.56 : 25]	0.08	2.4 (1.7–3.6)	2.5 (1.9–3.2)	**26[13 : 42]**	11[-7.9 : 35]	0.759
**50–59**	1.9 (1.3–2.7)	2.2 (1.5–3.1)	2.1 (1.5–2.9)	2.4 (1.7–3.2)	7.5[-1.8 : 18]	3.3[-8.6 : 17]	0.484	2.3 (1.7–3.2)	2.5 (1.7–3.5)	15[1.2 : 31]	-11[-29 : 12]	0.537
**60–69**	1.9 (1.4–2.7)	2.3 (1.5–3.1)	2.2 (1.5–3.2)	2.4 (1.8–3.5)	6.6[-3.5 : 18]	8.9[-4.7 : 24]	0.184	2.0 (1.3–2.7)	2.8 (1.9–3.8)	-9.5[-23 : 5.9]	5.4[-18 : 35]	**0.003**

Smoking and age-stratified median concentrations

AGE GROUP	Non-smokers		Smokers		% Change in Concentration smokers vs non-smokers		
	**Males** Concentration (pg/ml)	**Females** Concentration (pg/ml)	**Males** Concentration (pg/ml)	**Females** Concentration (pg/ml)	**Males** change (%)	**Females** change (%)	**Smoking × sex** Interaction P
**All**	2.2 (1.6–3.1)	2.5 (1.8–3.4)	1.9 (1.3–2.7)	2.0 (1.4–3.0)	**-12[-16 : -6.8]**	**-18[-22 : -14]**	0.052
**18–29**	2.3 (1.7–3.2)	2.7 (2.0–3.7)	2.3 (1.6–3.0)	2.3 (1.7–3.5)	-3.7[-12 : 5.9]	**-15[-22 : -6.4]**	0.073
**30–39**	2.3 (1.7–3.2)	2.5 (1.9–3.6)	2.1 (1.5–2.5)	2.2 (1.4–2.9)	**-19[-28 : -8.2]**	**-26[-34 : -16]**	0.322
**40–49**	2.2 (1.5–2.9)	2.3 (1.7–3.1)	1.6 (1.1–2.3)	1.9 (1.4–2.8)	**-17[-27 : -7.2]**	**-18[-26 : -7.8]**	0.996
**50–59**	2.1 (1.5–2.9)	2.3 (1.6–3.2)	1.7 (1.2–2.5)	1.9 (1.3–2.8)	**-19[-28 : -8.5]**	**-17[-27 : -6.1]**	0.79
**60–69**	2.1 (1.4–3.0)	2.4 (1.7–3.3)	1.9 (1.3–3.1)	1.8 (1.3–2.9)	2.9[-11 : 19]	**-15[-25 : -1.9]**	0.066

Supplementary Table 24: IL-17A/F (IL17F, Interleukin-17A and F heterodimer, Uniprot:Q96PD4)

Sex and age-stratified median concentrations

AGE GROUP	Biomarker/cohort information		Median Concentration			Age Group (% change in concentration)		
	**N measured**	**Detection Range** (% in range)	**Males** (pg/ml)	**Females** (pg/ml)	**Sex difference** F relative to M (%)	**Males** change/10 years (%)	**Females** change/10 years (%)	**Sex × age** Interaction P
**All**	9732	82.7	1.3 (0.72–2.0)	1.3 (0.76–2.0)	**0.65[-12 : 15]**	2.2[0.10 : 4.3]	**2.6[0.54 : 4.7]**	0.78
**18–29**	1972	81.7	1.2 (0.68–1.8)	1.3 (0.74–2.0)	4.8[-46 : 103]	5.9[-13 : 28]	6.3[-12 : 28]	0.981
**30–39**	1982	82.5	1.3 (0.72–1.9)	1.3 (0.71–1.9)	80[-41 : 443]	0.28[-20 : 25]	-16[-33 : 4.7]	0.265
**40–49**	1979	82.5	1.3 (0.69–1.9)	1.3 (0.76–2.0)	-18[-79 : 216]	7.6[-13 : 33]	14[-8.0 : 42]	0.696
**50–59**	1960	82.6	1.3 (0.77–2.0)	1.3 (0.76–2.0)	-45[-90 : 219]	-7.1[-26 : 17]	3.6[-17 : 30]	0.506
**60–69**	1839	84.4	1.3 (0.76–2.1)	1.4 (0.82–2.2)	-78[-99 : 372]	-10[-36 : 25]	14[-19 : 62]	0.325

BMI and age-stratified median concentrations

AGE GROUP	Normal BMI		Overweight BMI					Obese BMI				
	**Males** Concentration (pg/ml)	**Females** Concentration (pg/ml)	**Males** Concentration (pg/ml)	**Females** Concentration (pg/ml)	**Males** %change	**Females** %change	**Overweight × sex** Interaction P	**Males** Concentration (pg/ml)	**Females** Concentration (pg/ml)	**Males** %change	**Females** %change	**Obese × sex** Interaction P
**All**	1.2 (0.68–1.9)	1.3 (0.75–1.9)	1.3 (0.76–2.0)	1.3 (0.77–2.1)	6.3[0.050 : 13]	3.6[-4.5 : 12]	0.411	1.3 (0.77–1.9)	1.3 (0.74–2.0)	3.9[-5.0 : 14]	0.75[-13 : 17]	0.36
**18–29**	1.2 (0.65–1.8)	1.3 (0.77–1.9)	1.3 (0.77–2.0)	1.3 (0.69–2.0)	17[2.1 : 34]	3.0[-15 : 24]	0.163	1.2 (0.70–1.6)	1.4 (0.67–2.1)	-2.9[-23 : 22]	5.8[-25 : 49]	0.738
**30–39**	1.2 (0.69–1.9)	1.3 (0.68–1.8)	1.3 (0.74–1.9)	1.3 (0.73–2.0)	5.0[-8.8 : 21]	12[-7.0 : 35]	0.625	1.3 (0.76–1.9)	1.4 (0.82–2.0)	-1.1[-19 : 20]	17[-16 : 64]	0.32
**40–49**	1.2 (0.67–1.9)	1.3 (0.76–1.9)	1.3 (0.68–2.1)	1.3 (0.81–2.0)	10[-3.3 : 26]	3.9[-13 : 24]	0.554	1.3 (0.78–1.9)	1.3 (0.73–2.0)	15[-3.7 : 38]	-4.6[-29 : 29]	0.146
**50–59**	1.3 (0.67–2.0)	1.3 (0.71–1.9)	1.3 (0.78–2.0)	1.4 (0.78–2.2)	4.7[-9.1 : 21]	8.6[-10 : 31]	0.67	1.3 (0.88–2.1)	1.2 (0.73–2.0)	-0.39[-19 : 22]	11[-23 : 58]	0.577
**60–69**	1.2 (0.71–2.0)	1.4 (0.79–2.2)	1.3 (0.80–2.2)	1.3 (0.81–2.1)	-2.2[-15 : 13]	-3.7[-20 : 17]	0.759	1.4 (0.79–1.9)	1.4 (0.81–1.8)	1.4[-19 : 27]	-20[-44 : 15]	0.411

Smoking and age-stratified median concentrations

AGE GROUP	Non-smokers		Smokers		% Change in Concentration smokers vs non-smokers		
	**Males** Concentration (pg/ml)	**Females** Concentration (pg/ml)	**Males** Concentration (pg/ml)	**Females** Concentration (pg/ml)	**Males** change (%)	**Females** change (%)	**Smoking × sex** Interaction P
**All**	1.3 (0.73–2.0)	1.3 (0.76–2.0)	1.2 (0.65–2.0)	1.3 (0.73–2.0)	-7.1[-15 : 0.93]	**-0.82[-8.6 : 7.6]**	0.267
**18–29**	1.2 (0.70–1.8)	1.3 (0.75–2.0)	1.1 (0.64–1.9)	1.3 (0.70–1.9)	-5.3[-20 : 11]	-7.8[-21 : 8.2]	0.814
**30–39**	1.3 (0.74–1.9)	1.3 (0.74–1.9)	1.3 (0.62–2.0)	1.2 (0.54–1.7)	-17[-32 : 0.73]	-16[-30 : 1.8]	0.911
**40–49**	1.3 (0.71–2.0)	1.3 (0.75–1.9)	1.2 (0.66–2.0)	1.4 (0.90–2.2)	-2.0[-19 : 18]	15[-3.6 : 37]	0.223
**50–59**	1.3 (0.78–2.0)	1.3 (0.74–2.0)	1.2 (0.58–1.8)	1.3 (0.67–2.0)	-14[-29 : 4.6]	-7.0[-23 : 13]	0.595
**60–69**	1.3 (0.75–2.0)	1.4 (0.79–2.1)	1.4 (0.78–2.2)	1.6 (0.92–2.2)	4.9[-14 : 29]	15[-5.4 : 40]	0.518

Supplementary Table 25: IL-17B (Interleukin-17B, Uniprot:Q9UHF5)

Sex and age-stratified median concentrations

AGE GROUP	Biomarker/cohort information		Median Concentration			Age Group (% change in concentration)		
	**N measured**	**Detection Range** (% in range)	**Males** (pg/ml)	**Females** (pg/ml)	**Sex difference** F relative to M (%)	**Males** change/10 years (%)	**Females** change/10 years (%)	**Sex × age** Interaction P
**All**	9732	93.3	1.8 (1.1–2.6)	1.9 (1.2–2.8)	-21[-30 : -12]	0.73[-1.1 : 2.6]	**7.3[5.4 : 9.2]**	**9.06E-07**
**18–29**	1966	92.3	1.8 (1.2–2.7)	1.8 (1.1–2.5)	-8.7[-48 : 61]	-1.6[-17 : 16]	-0.40[-15 : 17]	0.919
**30–39**	1987	92.2	1.6 (1.1–2.5)	1.7 (1.0–2.6)	-13[-69 : 144]	6.2[-14 : 31]	9.1[-11 : 34]	0.855
**40–49**	1984	92.4	1.7 (1.1–2.5)	1.8 (1.1–2.7)	-36[-82 : 125]	-7.7[-24 : 12]	2.5[-16 : 25]	0.458
**50–59**	1959	94.3	1.8 (1.1–2.6)	2.0 (1.3–3.0)	-24[-82 : 230]	2.4[-15 : 24]	9.7[-9.3 : 33]	0.614
**60–69**	1836	95.5	1.9 (1.2–2.7)	2.1 (1.4–3.1)	4.0[-92 : 1247]	2.0[-23 : 35]	4.1[-22 : 40]	0.92

BMI and age-stratified median concentrations

AGE GROUP	Normal BMI		Overweight BMI					Obese BMI				
	**Males** Concentration (pg/ml)	**Females** Concentration (pg/ml)	**Males** Concentration (pg/ml)	**Females** Concentration (pg/ml)	**Males** %change	**Females** %change	**Overweight × sex** Interaction P	**Males** Concentration (pg/ml)	**Females** Concentration (pg/ml)	**Males** %change	**Females** %change	**Obese × sex** Interaction P
**All**	1.8 (1.1–2.6)	1.9 (1.2–2.8)	1.8 (1.2–2.6)	1.9 (1.2–2.9)	-2.3[-7.4 : 3.1]	5.8[-1.5 : 14]	0.918	1.7 (1.0–2.5)	1.8 (1.2–2.7)	-9.0[-16 : -1.5]	8.8[-4.7 : 24]	0.661
**18–29**	1.8 (1.2–2.7)	1.8 (1.1–2.5)	1.8 (1.2–2.6)	1.8 (1.1–2.7)	3.8[-7.7 : 17]	17[-0.85 : 37]	0.952	1.8 (1.1–2.4)	1.8 (1.2–2.2)	-9.3[-26 : 11]	19[-11 : 60]	0.956
**30–39**	1.6 (1.1–2.6)	1.8 (1.1–2.6)	1.7 (1.1–2.6)	1.8 (1.0–2.7)	-4.1[-16 : 9.4]	0.70[-15 : 20]	0.992	1.7 (1.1–2.5)	1.6 (0.88–2.5)	-3.7[-20 : 16]	-8.8[-34 : 25]	0.157
**40–49**	1.6 (1.0–2.4)	1.8 (1.2–2.7)	1.7 (1.2–2.6)	1.6 (1.1–2.7)	6.6[-5.6 : 21]	1.0[-14 : 19]	0.353	1.7 (0.96–2.5)	1.7 (1.1–2.4)	-4.6[-19 : 13]	4.6[-21 : 38]	0.889
**50–59**	1.8 (1.2–2.6)	2.0 (1.3–3.1)	1.8 (1.2–2.7)	2.0 (1.4–2.9)	-4.3[-15 : 7.7]	15[-2.0 : 35]	0.984	1.8 (0.96–2.4)	2.1 (1.3–2.8)	-12[-26 : 3.7]	35[0.54 : 83]	0.943
**60–69**	1.9 (1.2–2.7)	2.2 (1.5–3.2)	1.9 (1.3–2.6)	2.1 (1.3–3.3)	-0.75[-12 : 12]	-3.4[-18 : 14]	0.501	1.9 (1.2–2.7)	2.0 (1.6–2.9)	-2.0[-19 : 19]	-2.4[-28 : 32]	0.559

Smoking and age-stratified median concentrations

AGE GROUP	Non-smokers		Smokers		% Change in Concentration smokers vs non-smokers		
	**Males** Concentration (pg/ml)	**Females** Concentration (pg/ml)	**Males** Concentration (pg/ml)	**Females** Concentration (pg/ml)	**Males** change (%)	**Females** change (%)	**Smoking × sex** Interaction P
**All**	1.8 (1.2–2.6)	1.9 (1.2–2.8)	1.8 (1.0–2.7)	1.7 (1.0–2.4)	-3.9[-11 : 3.4]	**-18[-24 : -12]**	**0.002**
**18–29**	1.8 (1.2–2.6)	1.8 (1.1–2.6)	1.8 (1.1–2.7)	1.7 (1.1–2.3)	-2.3[-15 : 12]	-6.2[-18 : 7.8]	0.689
**30–39**	1.7 (1.1–2.5)	1.8 (1.1–2.6)	1.5 (0.99–2.8)	1.6 (0.86–2.4)	-5.6[-21 : 13]	**-21[-34 : -5.6]**	0.169
**40–49**	1.7 (1.1–2.5)	1.8 (1.2–2.7)	1.7 (1.1–2.6)	1.7 (1.0–2.4)	6.1[-11 : 26]	**-16[-29 : -1.2]**	0.053
**50–59**	1.8 (1.2–2.6)	2.1 (1.3–3.1)	1.8 (1.0–2.5)	1.8 (1.2–2.5)	-5.7[-20 : 11]	**-17[-30 : -2.6]**	0.259
**60–69**	1.9 (1.2–2.7)	2.2 (1.5–3.2)	1.9 (1.1–2.5)	1.7 (1.1–2.8)	-16[-29 : 0.28]	**-29[-40 : -16]**	0.15

Supplementary Table 26: IL-17C (Interleukin-17C, Uniprot:Q9P0M4)

Sex and age-stratified median concentrations

AGE GROUP	Biomarker/cohort information		Median Concentration			Age Group (% change in concentration)		
	N measured	Detection Range	Males	Females	Sex difference	Males	Females	Sex × age
		(% in range)	(pg/ml)	(pg/ml)	F relative to M (%)	change/10 years (%)	change/10 years (%)	Interaction P
**All**	9808	86.5	1.9 (1.1–2.9)	1.8 (1.0–2.7)	-12[-22 : 0.23]	2.3[0.26 : 4.3]	**3.0[1.0 : 5.1]**	0.597
**18–29**	1984	84.9	1.8 (1.0–2.8)	1.7 (0.99–2.6)	-6.1[-53 : 86]	14[-6.9 : 39]	15[-5.6 : 40]	0.942
**30–39**	2000	87.4	1.9 (1.1–2.8)	1.7 (0.99–2.7)	35[-50 : 265]	2.1[-17 : 25]	-9.7[-26 : 10]	0.394
**40–49**	1995	85.7	1.9 (1.1–3.0)	1.7 (0.97–2.6)	-67[-91 : 26]	-7.5[-25 : 14]	14[-7.7 : 42]	0.162
**50–59**	1977	87.4	2.0 (1.2–3.0)	1.8 (1.1–2.8)	-58[-92 : 125]	-9.7[-27 : 12]	4.7[-16 : 30]	0.349
**60–69**	1852	87.3	2.0 (1.1–3.2)	1.9 (1.2–2.9)	228[-83 : 6115]	-6.6[-32 : 29]	-24[-45 : 7.1]	0.398

BMI and age-stratified median concentrations

AGE GROUP	Normal BMI		Overweight BMI					Obese BMI				
	Males	Females	Males	Females	Males	Females	Overweight × sex	Males	Females	Males	Females	Obese × sex
	Concentration (pg/ml)	Concentration (pg/ml)	Concentration (pg/ml)	Concentration (pg/ml)	%change	%change	Interaction P	Concentration (pg/ml)	Concentration (pg/ml)	%change	%change	Interaction P
**All**	1.8 (1.0–2.8)	1.7 (1.0–2.6)	2.0 (1.2–3.0)	1.8 (1.1–2.7)	**9.2[2.9 : 16]**	2.9[-5.0 : 11]	0.566	2.1 (1.3–3.0)	1.9 (1.1–2.9)	13[3.9 : 24]	1.7[-12 : 18]	0.689
**18–29**	1.8 (0.99–2.7)	1.7 (0.94–2.5)	1.8 (1.1–2.8)	1.8 (1.1–2.6)	11[-3.5 : 28]	-3.9[-21 : 17]	0.551	2.3 (1.3–3.2)	1.9 (1.1–2.8)	31[2.9 : 66]	-4.5[-33 : 36]	0.444
**30–39**	1.8 (1.0–2.6)	1.6 (0.92–2.6)	1.9 (1.2–3.0)	1.7 (1.0–2.6)	12[-1.3 : 27]	-3.9[-19 : 14]	0.327	2.2 (1.3–2.7)	1.9 (1.1–3.1)	25[4.3 : 49]	1.7[-25 : 38]	0.739
**40–49**	1.9 (1.2–3.0)	1.7 (0.94–2.5)	2.0 (1.1–3.0)	1.7 (0.94–2.6)	3.0[-9.7 : 17]	-0.010[-16 : 19]	0.983	1.9 (1.3–2.6)	1.8 (1.1–2.7)	-0.76[-17 : 19]	2.3[-24 : 38]	0.385
**50–59**	1.8 (0.91–2.8)	1.7 (1.0–2.7)	2.0 (1.2–2.9)	1.8 (1.2–2.9)	14[-0.83 : 30]	20[0.070 : 44]	0.538	2.2 (1.5–3.5)	1.9 (0.95–2.9)	31[8.0 : 59]	10[-22 : 55]	0.243
**60–69**	2.0 (1.1–3.1)	1.9 (1.2–2.8)	2.0 (1.2–3.2)	1.8 (1.3–2.9)	0.62[-12 : 16]	2.4[-15 : 23]	0.955	2.0 (1.0–3.3)	1.9 (1.1–3.0)	-13[-30 : 8.7]	-0.40[-30 : 41]	0.611

Smoking and age-stratified median concentrations

AGE GROUP	Non-smokers		Smokers		% Change in Concentration smokers vs non-smokers		
	Males	Females	Males	Females	Males	Females	Smoking × sex
	Concentration (pg/ml)	Concentration (pg/ml)	Concentration (pg/ml)	Concentration (pg/ml)	change (%)	change (%)	Interaction P
**All**	1.9 (1.1–2.9)	1.7 (1.0–2.7)	1.9 (1.1–3.1)	1.8 (0.99–2.6)	6.9[-1.4 : 16]	-1.6[-9.1 : 6.4]	0.148
**18–29**	1.8 (1.0–2.8)	1.7 (0.99–2.5)	1.8 (0.97–2.8)	1.8 (1.0–2.6)	-1.1[-16 : 17]	6.8[-9.6 : 26]	0.522
**30–39**	1.9 (1.2–2.7)	1.7 (1.0–2.7)	1.8 (1.0–2.9)	1.4 (0.89–2.5)	1.8[-15 : 21]	-11[-25 : 5.9]	0.292
**40–49**	1.9 (1.1–2.9)	1.7 (0.98–2.6)	2.1 (1.2–3.6)	1.8 (0.93–2.5)	11[-7.5 : 34]	-7.2[-22 : 11]	0.16
**50–59**	2.0 (1.1–2.9)	1.8 (1.1–2.8)	2.3 (1.3–3.4)	1.8 (1.2–2.5)	**26[5.0 : 52]**	2.6[-15 : 24]	0.121
**60–69**	2.0 (1.2–3.1)	1.8 (1.2–2.9)	2.0 (0.97–3.3)	2.0 (1.1–2.8)	-1.0[-19 : 21]	-6.0[-22 : 14]	0.708

Supplementary Table 27: IL-17D (Interleukin-17D, Uniprot:Q8TAD2)

Sex and age-stratified median concentrations

AGE GROUP	Biomarker/cohort information		Median Concentration			Age Group (% change in concentration)		
	**N measured**	**Detection Range** (% in range)	**Males** (pg/ml)	**Females** (pg/ml)	**Sex difference** F relative to M (%)	**Males** change/10 years (%)	**Females** change/10 years (%)	**Sex × age** Interaction P
**All**	9810	94.3	8.5 (5.5–12)	8.7 (5.5–13)	**-21[-29 : -12]**	**19[17 : 21]**	**26[24 : 28]**	**3.35E-06**
**18–29**	1984	89.6	6.2 (3.8–8.7)	6.2 (3.6–9.0)	14[-40 : 117]	7.4[-11 : 29]	1.4[-16 : 22]	0.66
**30–39**	2000	92	7.7 (4.7–11)	7.0 (4.4–9.6)	88[-28 : 389]	**39[15 : 69]**	12[-7.7 : 36]	0.118
**40–49**	1995	94.7	8.6 (5.7–12)	8.5 (5.7–12)	57[-55 : 455]	**42[17 : 73]**	27[4.2 : 56]	0.44
**50–59**	1978	96.8	9.8 (6.9–14)	10 (7.5–14)	-48[-86 : 91]	13[-4.3 : 34]	**29[8.7 : 52]**	0.285
**60–69**	1853	98.3	11 (7.7–15)	13 (9.6–18)	-37[-94 : 511]	9.7[-14 : 40]	22[-6.0 : 58]	0.564

BMI and age-stratified median concentrations

AGE GROUP	Normal BMI		Overweight BMI					Obese BMI				
	**Males** Concentration (pg/ml)	**Females** Concentration (pg/ml)	**Males** Concentration (pg/ml)	**Females** Concentration (pg/ml)	**Males** %change	**Females** %change	**Overweight × sex** Interaction P	**Males** Concentration (pg/ml)	**Females** Concentration (pg/ml)	**Males** %change	**Females** %change	**Obese × sex** Interaction P
**All**	8.1 (5.1–12)	8.6 (5.4–13)	8.7 (5.8–13)	8.9 (5.7–13)	2.1[-3.1 : 7.5]	-0.78[-7.5 : 6.4]	0.322	9.0 (5.9–13)	8.3 (5.3–12)	2.9[-4.6 : 11]	-3.8[-15 : 9.3]	0.091
**18–29**	6.2 (3.5–8.7)	6.1 (3.3–9.0)	6.1 (4.4–8.5)	6.4 (4.2–9.2)	10[-3.4 : 26]	14[-5.0 : 37]	0.634	7.2 (4.3–11)	6.3 (3.9–8.8)	13[-9.3 : 42]	11[-20 : 55]	0.949
**30–39**	7.6 (4.7–10)	7.2 (4.5–9.7)	8.0 (4.6–11)	6.8 (4.4–9.3)	3.4[-8.4 : 17]	-9.3[-23 : 6.5]	0.216	7.9 (5.4–11)	7.3 (4.1–10)	5.3[-11 : 25]	-4.4[-29 : 28]	0.684
**40–49**	8.4 (5.4–12)	8.4 (5.6–11)	8.6 (5.8–13)	8.4 (5.5–12)	11[-1.7 : 26]	3.1[-12 : 21]	0.564	8.9 (6.2–13)	8.6 (5.4–11)	14[-3.9 : 35]	-4.4[-28 : 27]	0.313
**50–59**	9.8 (6.9–14)	11 (8.0–15)	9.9 (7.0–14)	10 (7.6–14)	1.1[-8.9 : 12]	-5.2[-18 : 9.1]	0.213	9.7 (6.5–13)	8.5 (5.2–13)	-12[-24 : 2.0]	-18[-37 : 6.3]	0.193
**60–69**	12 (8.0–16)	15 (10–19)	11 (7.3–15)	13 (8.8–17)	-1.9[-12 : 9.2]	-0.42[-14 : 15]	0.406	11 (8.3–15)	13 (9.2–17)	11[-6.5 : 31]	9.7[-16 : 44]	0.102

Smoking and age-stratified median concentrations

AGE GROUP	Non-smokers		Smokers		% Change in Concentration smokers vs non-smokers		
	**Males** Concentration (pg/ml)	**Females** Concentration (pg/ml)	**Males** Concentration (pg/ml)	**Females** Concentration (pg/ml)	**Males** change (%)	**Females** change (%)	**Smoking × sex** Interaction P
**All**	8.5 (5.5–12)	8.7 (5.5–13)	8.8 (5.4–13)	8.5 (5.4–13)	**8.9[1.5 : 17]**	2.3[-4.5 : 9.7]	0.214
**18–29**	6.3 (4.0–8.6)	6.1 (3.4–9.3)	5.7 (3.5–9.0)	6.2 (3.7–8.6)	-6.5[-20 : 9.4]	-2.8[-17 : 14]	0.727
**30–39**	7.7 (4.8–11)	7.1 (4.4–9.6)	8.1 (4.3–11)	6.8 (4.4–9.6)	14[-3.7 : 35]	0.87[-14 : 19]	0.309
**40–49**	8.6 (5.6–12)	8.3 (5.6–12)	9.1 (7.1–12)	8.7 (5.3–11)	**20[1.1 : 43]**	1.4[-14 : 20]	0.161
**50–59**	9.8 (6.8–14)	11 (7.5–14)	11 (8.0–15)	10. (7.7–14)	12[-3.1 : 29]	-2.1[-15 : 13]	0.202
**60–69**	11 (7.7–15)	13 (9.5–18)	12 (8.5–15)	15 (9.9–19)	8.6[-6.8 : 26]	14[-1.3 : 32]	0.641

Supplementary Table 28: CCL2 (monocyte chemoattractant protein 1, MCP1, GDCF-2, HC11, Uniprot:P13500)

Sex and age-stratified median concentrations

AGE GROUP	Biomarker/cohort information		Median Concentration			Age Group (% change in concentration)		
	N measured	Detection Range	Males	Females	Sex difference	Males	Females	Sex × age
		(% in range)	(pg/ml)	(pg/ml)	F relative to M (%)	change/10 years (%)	change/10 years (%)	Interaction P
**All**	9445	100	81 (68–100)	74 (59–93)	**-22[-26 : -18]**	**5.7[4.8 : 6.5]**	**9.3[8.4 : 10]**	**3.33E-09**
**18–29**	1866	100	73 (62–87)	65 (54–80)	-15[-33 : 7.5]	7.1[0.14 : 15]	9.5[2.3 : 17]	0.644
**30–39**	1903	99.9	78 (65–96)	65 (54–81)	61[0.88 : 158]	9.8[-0.070 : 21]	-8.9[-17 : 0.070]	0.006
**40–49**	1920	99.9	81 (68–101)	69 (57–85)	-7.7[-45 : 55]	7.4[-0.86 : 16]	5.4[-3.0 : 15]	0.749
**50–59**	1920	99.9	85 (71–104)	82 (67–100)	-29[-62 : 33]	8.0[-0.48 : 17]	**15[5.6 : 24]**	0.315
**60–69**	1836	100	90 (74–110)	89 (74–112)	1.1[-72 : 264]	5.0[-8.7 : 21]	4.4[-9.8 : 21]	0.962

BMI and age-stratified median concentrations

AGE GROUP	Normal BMI		Overweight BMI					Obese BMI				
	Males	Females	Males	Females	Males	Females	Overweight × sex	Males	Females	Males	Females	Obese × sex
	Concentration (pg/ml)	Concentration (pg/ml)	Concentration (pg/ml)	Concentration (pg/ml)	%change	%change	Interaction P	Concentration (pg/ml)	Concentration (pg/ml)	%change	%change	Interaction P
**All**	78 (65–94)	72 (58–91)	83 (69–103)	76 (61–94)	3.1[0.69 : 5.6]	0.52[-2.6 : 3.8]	0.588	87 (72–105)	77 (62–95)	**8.1[4.4 : 12]**	-3.1[-8.7 : 2.7]	0.004
**18–29**	72 (61–84)	64 (52–78)	74 (62–90)	68 (56–84)	1.0[-3.7 : 6.0]	3.5[-3.2 : 11]	0.17	82 (66–101)	72 (58–88)	**17[7.9 : 27]**	5.4[-6.6 : 19]	0.417
**30–39**	76 (64–92)	64 (52–79)	77 (65–98)	66 (56–79)	6.4[0.30 : 13]	1.2[-6.4 : 9.5]	0.336	83 (71–103)	71 (58–87)	**15[6.0 : 25]**	3.3[-10 : 19]	0.124
**40–49**	78 (66–94)	69 (56–84)	82 (70–104)	69 (57–85)	5.2[-0.020 : 11]	0.48[-6.0 : 7.5]	0.357	86 (72–107)	70 (59–90)	7.7[0.46 : 15]	-0.77[-12 : 11]	0.259
**50–59**	82 (70–102)	81 (67–99)	87 (74–105)	84 (68–102)	4.9[-0.32 : 10]	1.7[-5.0 : 8.9]	0.515	88 (74–105)	86 (71–103)	5.7[-1.7 : 14]	2.1[-10 : 16]	0.716
**60–69**	88 (74–108)	91 (74–114)	91 (74–111)	89 (73–114)	2.1[-3.9 : 8.5]	-5.1[-12 : 2.8]	0.444	90 (77–112)	88 (76–103)	3.6[-5.8 : 14]	-19[-30 : -5.9]	0.02

Smoking and age-stratified median concentrations

AGE GROUP	Non-smokers		Smokers		% Change in Concentration smokers vs non-smokers		
	Males	Females	Males	Females	Males	Females	Smoking × sex
	Concentration (pg/ml)	Concentration (pg/ml)	Concentration (pg/ml)	Concentration (pg/ml)	change (%)	change (%)	Interaction P
**All**	80 (67–99)	73 (59–92)	86 (71–104)	78 (62–99)	**8.4[4.9 : 12]**	**6.6[3.3 : 10]**	0.476
**18–29**	72 (61–85)	64 (53–79)	78 (65–93)	70 (57–82)	**7.6[1.6 : 14]**	**7.1[1.2 : 13]**	0.918
**30–39**	77 (65–96)	65 (54–80)	81 (68–99)	65 (55–84)	7.2[-1.3 : 17]	-3.5[-11 : 4.6]	0.073
**40–49**	80 (68–100)	69 (56–83)	88 (73–112)	75 (59–93)	**9.3[1.7 : 17]**	**7.9[0.76 : 16]**	0.795
**50–59**	85 (71–103)	81 (67–100)	92 (75–115)	89 (75–104)	**9.3[2.0 : 17]**	**7.9[0.65 : 16]**	0.804
**60–69**	89 (74–110)	89 (74–111)	96 (80–120)	98 (78–129)	**9.5[0.32 : 19]**	**12[2.9 : 21]**	0.742

Supplementary Table 29: CCL3 (macrophage inflammatory protein 1α, SCYA3, MIP1α, Uniprot:P10147)

Sex and age-stratified median concentrations

AGE GROUP	Biomarker/cohort information: N measured	Biomarker/cohort information: Detection Range (% in range)	Median Concentration: Males (pg/ml)	Median Concentration: Females (pg/ml)	Median Concentration: Sex difference F relative to M (%)	Age Group (% change in concentration): Males change/10 years (%)	Age Group: Females change/10 years (%)	Age Group: Sex × age Interaction P
**All**	9445	99	13 (11–16)	13 (11–16)	-3.3[-8.8 : 2.5]	**4.0[3.0 : 4.9]**	**4.4[3.5 : 5.3]**	0.513
**18–29**	1866	98.5	13 (10–15)	12 (10–15)	22[-11 : 69]	10[0.44 : 21]	0.78[-8.2 : 11]	0.179
**30–39**	1903	98.5	13 (10–15)	12 (10.–15)	67[6.1 : 164]	-0.020[-8.8 : 9.6]	**-14[-22 : -6.0]**	0.02
**40–49**	1920	99.2	13 (11–16)	13 (10–15)	-12[-53 : 66]	0.53[-8.8 : 11]	2.5[-7.4 : 13]	0.789
**50–59**	1920	99.3	14 (11–16)	13 (11–16)	-15[-59 : 80]	7.3[-2.5 : 18]	10[0.20 : 21]	0.689
**60–69**	1836	99.4	15 (12–17)	14 (12–17)	213[-4.9 : 934]	16[2.2 : 33]	-2.7[-15 : 12]	0.062

BMI and age-stratified median concentrations

AGE GROUP	Normal BMI: Males Concentration (pg/ml)	Normal BMI: Females Concentration (pg/ml)	Overweight BMI: Males Concentration (pg/ml)	Overweight BMI: Females Concentration (pg/ml)	Overweight BMI: Males %change	Overweight BMI: Females %change	Overweight × sex Interaction P	Obese BMI: Males Concentration (pg/ml)	Obese BMI: Females Concentration (pg/ml)	Obese BMI: Males %change	Obese BMI: Females %change	Obese × sex Interaction P
**All**	13 (10–15)	12 (10–15)	14 (11–16)	13 (11–16)	**4.9[2.2 : 7.7]**	2.2[-1.4 : 5.9]	0.945	15 (12–17)	14 (12–17)	**17[12 : 21]**	**10[3.5 : 18]**	0.649
**18–29**	12 (10.–14)	12 (9.7–14)	13 (10–16)	12 (10–15)	7.0[0.14 : 14]	-2.4[-11 : 7.1]	0.908	14 (12–17)	14 (12–16)	**24[11 : 39]**	-4.0[-19 : 13]	0.44
**30–39**	12 (9.8–15)	12 (9.6–14)	13 (10–15)	12 (9.9–15)	6.8[0.89 : 13]	3.6[-4.0 : 12]	0.336	14 (12–17)	14 (12–17)	**23[13 : 33]**	**24[7.8 : 42]**	0.743
**40–49**	13 (10–15)	12 (9.8–15)	13 (11–16)	13 (10–15)	6.1[-0.20 : 13]	1.5[-6.5 : 10]	0.513	14 (12–17)	14 (12–16)	**21[11 : 31]**	20[4.0 : 38]	0.629
**50–59**	13 (11–16)	13 (11–16)	14 (11–16)	14 (11–16)	1.2[-4.7 : 7.5]	0.15[-7.6 : 8.5]	0.437	14 (13–17)	15 (12–18)	11[1.5 : 21]	6.2[-8.8 : 24]	0.316
**60–69**	14 (12–17)	14 (12–16)	15 (12–17)	15 (13–17)	4.1[-1.6 : 10]	5.3[-2.2 : 13]	0.288	16 (14–18)	15 (13–19)	12[2.7 : 23]	6.1[-7.8 : 22]	0.784

Smoking and age-stratified median concentrations

AGE GROUP	Non-smokers: Males Concentration (pg/ml)	Non-smokers: Females Concentration (pg/ml)	Smokers: Males Concentration (pg/ml)	Smokers: Females Concentration (pg/ml)	% Change in Concentration smokers vs non-smokers: Males change (%)	% Change: Females change (%)	Smoking × sex Interaction P
**All**	13 (11–16)	13 (11–16)	13 (11–16)	13 (11–16)	2.1[-1.5 : 5.9]	**4.0[0.36 : 7.8]**	0.484
**18–29**	13 (10–15)	12 (10.–15)	13 (11–16)	13 (10–15)	1.5[-6.2 : 9.8]	3.5[-4.3 : 12]	0.735
**30–39**	13 (10–15)	12 (10–15)	12 (10–14)	12 (9.6–15)	1.9[-6.0 : 10]	-6.2[-13 : 1.5]	0.152
**40–49**	13 (11–16)	13 (10–15)	14 (11–16)	13 (10–15)	2.2[-6.4 : 11]	**9.4[0.71 : 19]**	0.263
**50–59**	14 (11–17)	13 (11–16)	14 (12–16)	14 (11–17)	-0.66[-8.4 : 7.7]	6.0[-2.4 : 15]	0.27
**60–69**	15 (12–17)	14 (12–17)	15 (13–19)	15 (12–17)	3.4[-4.6 : 12]	5.5[-2.3 : 14]	0.725

Supplementary Table 30: CCL4 (macrophage inflammatory protein 1β, MIP1β, SCYA4, LAG1, Uniprot:P13236)

Sex and age-stratified median concentrations

AGE GROUP	Biomarker/cohort information		Median Concentration			Age Group (% change in concentration)		
	**N measured**	**Detection Range** (% in range)	**Males** (pg/ml)	**Females** (pg/ml)	**Sex difference** F relative to M (%)	**Males** change/10 years (%)	**Females** change/10 years (%)	**Sex × age** Interaction P
**All**	9445	99.9	76 (58–100)	71 (53–95)	**-13[-18 : -7.1]**	**5.0[4.0 : 6.0]**	**6.8[5.8 : 7.8]**	0.015
**18–29**	1866	100	70 (54–90)	63 (47–85)	16[-15 : 59]	11[1.5 : 22]	1.0[-7.8 : 11]	0.142
**30–39**	1903	99.9	72 (56–94)	65 (49–87)	20[-30 : 106]	11[-0.87 : 23]	2.3[-8.3 : 14]	0.323
**40–49**	1920	99.9	76 (58–100)	69 (53–93)	-20[-59 : 56]	5.6[-4.7 : 17]	8.7[-2.3 : 21]	0.696
**50–59**	1920	99.9	79 (62–105)	76 (58–102)	2.8[-55 : 134]	9.3[-1.7 : 22]	8.2[-2.7 : 20]	0.891
**60–69**	1836	99.9	85 (66–110)	82 (63–108)	237[-13 : 1199]	23[5.9 : 42]	0.93[-14 : 18]	0.073

BMI and age-stratified median concentrations

AGE GROUP	Normal BMI		Overweight BMI					Obese BMI				
	**Males** Concentration (pg/ml)	**Females** Concentration (pg/ml)	**Males** Concentration (pg/ml)	**Females** Concentration (pg/ml)	**Males** %change	**Females** %change	**Overweight × sex** Interaction P	**Males** Concentration (pg/ml)	**Females** Concentration (pg/ml)	**Males** %change	**Females** %change	**Obese × sex** Interaction P
**All**	73 (56–94)	68 (51–91)	77 (60–101)	74 (55–100)	3.4[0.45 : 6.4]	2.4[-1.5 : 6.4]	0.112	88 (68–118)	77 (59–102)	**19[14 : 24]**	-1.8[-8.5 : 5.4]	**0.003**
**18–29**	67 (52–85)	60 (45–81)	73 (55–92)	70 (50–92)	5.2[-1.4 : 12]	4.4[-4.6 : 14]	0.21	88 (70–117)	75 (51–90)	**28[15 : 43]**	-1.1[-16 : 16]	0.17
**30–39**	69 (53–85)	60 (46–80)	71 (56–94)	67 (52–91)	6.7[-0.33 : 14]	14[3.8 : 24]	0.114	89 (67–113)	77 (59–100)	**33[21 : 46]**	21[2.6 : 42]	0.437
**40–49**	74 (56–93)	66 (52–93)	75 (57–102)	70 (53–92)	3.8[-2.7 : 11]	-2.9[-11 : 5.8]	0.477	89 (67–118)	72 (56–93)	**23[12 : 34]**	-5.3[-18 : 9.9]	**0.004**
**50–59**	77 (57–107)	74 (58–99)	79 (62–103)	78 (58–105)	2.8[-3.7 : 9.9]	-0.97[-9.4 : 8.2]	0.828	87 (67–118)	77 (60–108)	**15[4.2 : 26]**	-7.4[-22 : 9.6]	0.148
**60–69**	83 (65–110)	79 (62–103)	84 (66–110)	84 (64–115)	2.3[-4.0 : 9.1]	0.94[-7.2 : 9.8]	0.357	89 (71–114)	90 (68–113)	4.2[-5.8 : 15]	-8.2[-22 : 7.7]	0.956

Smoking and age-stratified median concentrations

AGE GROUP	Non-smokers		Smokers		% Change in Concentration smokers vs non-smokers		
	**Males** Concentration (pg/ml)	**Females** Concentration (pg/ml)	**Males** Concentration (pg/ml)	**Females** Concentration (pg/ml)	**Males** change (%)	**Females** change (%)	**Smoking × sex** Interaction P
**All**	76 (58–100)	71 (53–95)	76 (60–103)	71 (52–94)	1.9[-2.1 : 6.0]	-2.2[-5.9 : 1.6]	0.147
**18–29**	71 (54–91)	64 (48–86)	66 (55–90)	58 (44–83)	-2.4[-9.6 : 5.4]	**-7.9[-15 : -0.54]**	0.294
**30–39**	72 (55–93)	64 (49–88)	74 (62–97)	66 (48–81)	4.4[-5.1 : 15]	-5.8[-14 : 3.3]	0.13
**40–49**	76 (57–100)	68 (53–93)	79 (66–105)	66 (51–90)	6.8[-2.6 : 17]	-3.7[-12 : 5.1]	0.111
**50–59**	78 (62–105)	75 (58–102)	81 (62–106)	78 (61–106)	2.8[-6.0 : 12]	3.8[-5.3 : 14]	0.886
**60–69**	84 (66–110)	82 (63–108)	83 (66–110)	84 (65–105)	-2.4[-11 : 7.0]	3.0[-5.6 : 12]	0.407

Supplementary Table 31: CCL11 (Eotaxin, Uniprot:P51671)

Sex and age-stratified median concentrations

AGE GROUP	Biomarker/cohort information		Median Concentration			Age Group (% change in concentration)		
	**N measured**	**Detection Range**	**Males**	**Females**	**Sex difference**	**Males**	**Females**	**Sex × age**
		(% in range)	(pg/ml)	(pg/ml)	F relative to M (%)	change/10 years (%)	change/10 years (%)	Interaction P
**All**	9445	100	225 (182–292)	203 (154–267)	**-33[-37 : -30]**	**9.5[8.7 : 10]**	**17[16 : 17]**	**9.24E-29**
**18–29**	1866	100	192 (159–231)	151 (124–198)	-28[-44 : -7.6]	**15[7.3 : 24]**	**21[13 : 30]**	0.339
**30–39**	1903	99.9	213 (173–267)	170 (138–215)	-14[-43 : 31]	11[1.8 : 21]	7.9[-0.83 : 18]	0.671
**40–49**	1920	99.9	225 (184–287)	199 (159–254)	-19[-52 : 37]	**13[4.6 : 23]**	**15[5.4 : 25]**	0.854
**50–59**	1920	99.9	249 (202–321)	236 (192–300)	-22[-59 : 49]	10[1.2 : 20]	**14[4.9 : 24]**	0.547
**60–69**	1836	100	260 (213–338)	261 (215–356)	-49[-84 : 60]	-3.7[-15 : 9.0]	6.8[-6.2 : 22]	0.258

BMI and age-stratified median concentrations

AGE GROUP	Normal BMI		Overweight BMI					Obese BMI				
	**Males**	**Females**	**Males**	**Females**	**Males**	**Females**	**Overweight × sex**	**Males**	**Females**	**Males**	**Females**	**Obese × sex**
	Concentration (pg/ml)	Concentration (pg/ml)	Concentration (pg/ml)	Concentration (pg/ml)	%change	%change	Interaction P	Concentration (pg/ml)	Concentration (pg/ml)	%change	%change	Interaction P
**All**	220 (179–286)	203 (153–269)	231 (186–298)	207 (160–277)	-2.8[-5.0 : -0.48]	-0.18[-3.2 : 3.0]	0.905	226 (182–295)	190 (147–243)	-2.4[-5.7 : 0.95]	-4.0[-9.4 : 1.6]	**0.002**
**18–29**	193 (158–226)	150 (124–197)	192 (162–235)	154 (126–199)	-1.7[-6.6 : 3.4]	2.8[-4.3 : 10]	0.561	188 (160–234)	145 (108–195)	-0.70[-8.8 : 8.2]	-5.2[-17 : 7.8]	0.093
**30–39**	214 (175–265)	172 (143–222)	212 (172–269)	166 (132–204)	0.68[-4.5 : 6.2]	-2.8[-9.4 : 4.3]	0.074	215 (174–272)	165 (138–203)	-0.46[-7.6 : 7.2]	4.1[-8.4 : 18]	0.35
**40–49**	225 (185–288)	205 (162–256)	225 (183–286)	195 (161–252)	-0.37[-5.3 : 4.8]	-1.5[-7.9 : 5.4]	0.678	225 (184–295)	187 (139–234)	3.1[-3.8 : 11]	-9.2[-19 : 2.0]	0.005
**50–59**	252 (206–330)	239 (192–312)	250 (202–322)	241 (194–296)	-1.9[-6.9 : 3.4]	-0.69[-7.4 : 6.5]	0.586	240 (198–305)	216 (185–268)	-5.9[-13 : 1.5]	-0.77[-13 : 13]	0.666
**60–69**	268 (216–346)	268 (221–371)	259 (208–340)	261 (210–335)	-3.3[-8.4 : 2.0]	-1.1[-7.9 : 6.2]	0.81	255 (216–316)	235 (186–303)	-5.5[-13 : 2.9]	-5.9[-18 : 7.6]	0.177

Smoking and age-stratified median concentrations

AGE GROUP	Non-smokers		Smokers		% Change in Concentration smokers vs non-smokers		
	**Males**	**Females**	**Males**	**Females**	**Males**	**Females**	**Smoking × sex**
	Concentration (pg/ml)	Concentration (pg/ml)	Concentration (pg/ml)	Concentration (pg/ml)	change (%)	change (%)	Interaction P
**All**	222 (181–286)	200 (152–259)	252 (191–345)	222 (167–308)	**15[12 : 19]**	**15[12 : 19]**	0.94
**18–29**	193 (158–229)	150 (123–194)	192 (164–234)	169 (127–212)	1.7[-4.3 : 8.0]	**8.8[2.5 : 16]**	0.12
**30–39**	212 (173–267)	168 (137–213)	225 (180–282)	177 (142–223)	6.9[-0.82 : 15]	3.6[-3.6 : 11]	0.556
**40–49**	219 (183–282)	194 (155–245)	273 (218–339)	228 (173–305)	**18[9.6 : 27]**	**20[12 : 28]**	0.736
**50–59**	243 (200–306)	232 (189–294)	320 (238–410)	278 (225–393)	**28[19 : 37]**	**24[15 : 33]**	0.527
**60–69**	256 (208–330)	252 (211–338)	335 (259–460)	316 (260–440)	**25[16 : 35]**	**24[15 : 33]**	0.853

Supplementary Table 32: CCL13 (monocyte chemoattractant protein 4, MCP4, Uniprot:Q99616)

Sex and age-stratified median concentrations

AGE GROUP	Biomarker/cohort information		Median Concentration			Age Group (% change in concentration)		
	**N measured**	**Detection Range** (% in range)	**Males** (pg/ml)	**Females** (pg/ml)	**Sex difference** F relative to M (%)	**Males** change/10 years (%)	**Females** change/10 years (%)	**Sex × age** Interaction P
**All**	9445	100	155 (112–208)	156 (111–212)	**-12[-17 : -6.2]**	**10[9.2 : 11]**	**14[13 : 15]**	**2.32E-05**
**18–29**	1866	100	125 (90–167)	120 (85–163)	-13[-39 : 24]	11[0.41 : 23]	16[4.4 : 28]	0.584
**30–39**	1903	99.9	140 (104–184)	137 (99–183)	33[-21 : 126]	**19[6.7 : 32]**	7.9[-2.9 : 20]	0.217
**40–49**	1920	100	155 (115–207)	157 (115–205)	-36[-67 : 25]	6.6[-3.8 : 18]	**18[5.6 : 31]**	0.195
**50–59**	1920	99.9	175 (128–228)	181 (134–241)	-23[-66 : 74]	8.3[-2.5 : 20]	15[3.3 : 27]	0.445
**60–69**	1836	100	186 (140–240)	195 (145–263)	35[-66 : 435]	8.7[-6.4 : 26]	4.4[-11 : 22]	0.72

BMI and age-stratified median concentrations

AGE GROUP	Normal BMI		Overweight BMI					Obese BMI				
	**Males** Concentration (pg/ml)	**Females** Concentration (pg/ml)	**Males** Concentration (pg/ml)	**Females** Concentration (pg/ml)	**Males** %change	**Females** %change	**Overweight × sex** Interaction P	**Males** Concentration (pg/ml)	**Females** Concentration (pg/ml)	**Males** %change	**Females** %change	**Obese × sex** Interaction P
**All**	146 (106–196)	148 (105–202)	160 (116–215)	165 (122–228)	1.0[-1.9 : 4.0]	3.4[-0.55 : 7.6]	**9.03E-04**	174 (125–229)	174 (128–230)	**12[7.5 : 17]**	0.30[-6.7 : 7.8]	0.864
**18–29**	124 (87–161)	113 (80–157)	124 (93–173)	130 (87–178)	1.9[-5.3 : 9.6]	7.8[-2.7 : 19]	0.041	146 (102–191)	149 (100–181)	13[0.16 : 28]	5.1[-13 : 26]	0.525
**30–39**	136 (102–176)	126 (96–168)	140 (106–186)	142 (101–192)	7.0[0.18 : 14]	8.1[-0.97 : 18]	0.295	168 (112–229)	157 (111–212)	**24[13 : 36]**	7.7[-8.1 : 26]	0.676
**40–49**	151 (113–201)	155 (113–204)	160 (115–209)	157 (114–208)	1.9[-4.5 : 8.8]	-8.3[-16 : -0.11]	0.69	166 (130–222)	164 (121–207)	13[3.2 : 23]	-17[-29 : -4.1]	0.122
**50–59**	171 (123–221)	174 (122–230)	173 (128–227)	190 (142–244)	1.2[-5.2 : 8.0]	7.1[-1.9 : 17]	0.141	190 (148–258)	194 (156–260)	14[3.5 : 25]	11[-6.1 : 31]	0.943
**60–69**	186 (143–243)	186 (140–247)	185 (137–245)	202 (151–275)	-2.9[-9.1 : 3.6]	5.0[-3.7 : 14]	0.025	192 (144–248)	221 (161–280)	0.61[-9.2 : 11]	6.9[-9.1 : 26]	0.084

Smoking and age-stratified median concentrations

AGE GROUP	Non-smokers		Smokers		% Change in Concentration smokers vs non-smokers		
	**Males** Concentration (pg/ml)	**Females** Concentration (pg/ml)	**Males** Concentration (pg/ml)	**Females** Concentration (pg/ml)	**Males** change (%)	**Females** change (%)	**Smoking × sex** Interaction P
**All**	156 (113–209)	156 (111–211)	156 (113–212)	159 (114–222)	3.0[-1.0 : 7.3]	**4.2[0.15 : 8.3]**	0.706
**18–29**	124 (91–168)	118 (83–159)	128 (90–166)	130 (89–186)	-0.30[-8.6 : 8.7]	**12[2.5 : 22]**	0.068
**30–39**	142 (105–185)	137 (100–183)	135 (103–179)	140 (95–203)	-0.33[-9.2 : 9.4]	-2.0[-10 : 7.2]	0.794
**40–49**	155 (115–206)	158 (114–205)	171 (122–228)	154 (116–209)	7.3[-2.2 : 18]	-0.47[-8.8 : 8.7]	0.248
**50–59**	174 (128–227)	180 (134–241)	179 (129–234)	190 (146–250)	4.6[-4.2 : 14]	8.5[-0.79 : 19]	0.571
**60–69**	185 (141–239)	196 (145–264)	201 (153–270)	194 (149–269)	7.5[-2.1 : 18]	2.0[-6.6 : 11]	0.422

Supplementary Table 33: CCL17 (thymus- and activation-regulated chemokine, TARC, ABCD-2, SCYA17, Uniprot:Q92583)

Sex and age-stratified median concentrations

AGE GROUP	Biomarker/cohort information		Median Concentration			Age Group (% change in concentration)		
	N measured	Detection Range	Males	Females	Sex difference	Males	Females	Sex × age
		(% in range)	(pg/ml)	(pg/ml)	F relative to M (%)	change/10 years (%)	change/10 years (%)	Interaction P
**All**	9445	100	318 (212–477)	301 (199–442)	**-17[-24 : -8.8]**	**5.9[4.4 : 7.4]**	**8.5[7.0 : 10]**	0.014
**18–29**	1866	100	301 (194–447)	265 (176–387)	-9.3[-44 : 47]	-7.6[-20 : 6.2]	-8.8[-21 : 4.9]	0.894
**30–39**	1903	99.9	298 (194–432)	266 (179–395)	4.8[-52 : 129]	4.6[-11 : 22]	0.70[-14 : 18]	0.741
**40–49**	1920	100	310 (210–451)	285 (187–423)	-39[-76 : 54]	-0.070[-13 : 15]	9.2[-5.9 : 27]	0.4
**50–59**	1920	99.9	347 (238–519)	327 (227–479)	57[-50 : 393]	7.1[-7.5 : 24]	-2.0[-15 : 13]	0.399
**60–69**	1836	99.9	357 (239–532)	370 (242–545)	56[-80 : 1146]	7.4[-14 : 35]	-0.26[-21 : 27]	0.658

BMI and age-stratified median concentrations

AGE GROUP	Normal BMI		Overweight BMI					Obese BMI				
	Males	Females	Males	Females	Males	Females	Overweight × sex	Males	Females	Males	Females	Obese × sex
	Concentration (pg/ml)	Concentration (pg/ml)	Concentration (pg/ml)	Concentration (pg/ml)	%change	%change	Interaction P	Concentration (pg/ml)	Concentration (pg/ml)	%change	%change	Interaction P
**All**	309 (209–466)	292 (191–432)	322 (215–476)	308 (211–465)	-1.1[-5.1 : 3.1]	2.6[-3.0 : 8.5]	0.058	337 (221–510)	313 (203–455)	2.5[-3.6 : 9.1]	-1.8[-11 : 8.9]	0.808
**18–29**	298 (189–441)	257 (173–375)	297 (197–420)	289 (193–402)	3.6[-6.1 : 14]	6.0[-7.6 : 22]	0.501	353 (205–513)	302 (178–441)	7.8[-8.6 : 27]	4.3[-19 : 34]	0.794
**30–39**	287 (200–401)	259 (170–388)	307 (194–447)	272 (186–388)	7.6[-2.5 : 19]	7.8[-5.3 : 23]	0.838	308 (194–446)	311 (187–441)	8.0[-5.9 : 24]	6.7[-16 : 35]	0.822
**40–49**	307 (212–444)	274 (183–404)	314 (209–461)	297 (198–450)	-1.4[-10. : 8.0]	-1.2[-12 : 11]	0.202	328 (217–510)	302 (193–438)	6.2[-6.2 : 20]	-15[-31 : 4.2]	0.866
**50–59**	344 (229–530)	319 (224–495)	344 (241–517)	313 (226–469)	-1.7[-10 : 7.7]	-1.8[-13 : 11]	0.786	376 (244–555)	329 (243–457)	7.6[-5.7 : 23]	-3.5[-24 : 22]	0.508
**60–69**	362 (233–541)	369 (234–561)	360 (247–520)	372 (248–529)	-5.1[-14 : 4.7]	8.3[-4.8 : 23]	0.219	340 (237–567)	353 (248–542)	-9.8[-23 : 5.3]	14[-11 : 45]	0.234

Smoking and age-stratified median concentrations

AGE GROUP	Non-smokers		Smokers		% Change in Concentration smokers vs non-smokers		
	Males	Females	Males	Females	Males	Females	Smoking × sex
	Concentration (pg/ml)	Concentration (pg/ml)	Concentration (pg/ml)	Concentration (pg/ml)	change (%)	change (%)	Interaction P
**All**	311 (209–461)	291 (192–425)	381 (248–595)	384 (249–587)	**28[21 : 36]**	**32[25 : 40]**	0.407
**18–29**	294 (191–430)	260 (173–374)	337 (214–532)	311 (199–486)	**24[10. : 39]**	**20[6.9 : 35]**	0.73
**30–39**	298 (193–428)	260 (170–386)	300 (223–453)	316 (217–459)	8.2[-5.8 : 24]	**21[6.3 : 39]**	0.236
**40–49**	301 (204–436)	275 (183–404)	448 (273–638)	394 (258–619)	**39[22 : 58]**	**44[28 : 63]**	0.643
**50–59**	336 (231–506)	310 (218–459)	436 (306–651)	428 (291–601)	**33[18 : 51]**	**33[17 : 51]**	0.983
**60–69**	350 (235–509)	351 (235–513)	511 (298–819)	477 (309–731)	**43[24 : 65]**	**42[24 : 62]**	0.921

Supplementary Table 34: CCL22 (macrophage derived chemokine, MDC, ABCD-1, Uniprot:O00626)

Sex and age-stratified median concentrations

AGE GROUP	Biomarker/cohort information		Median Concentration			Age Group (% change in concentration)		
	N measured	Detection Range	Males	Females	Sex difference	Males	Females	Sex × age
		(% in range)	(pg/ml)	(pg/ml)	F relative to M (%)	change/10 years (%)	change/10 years (%)	Interaction P
**All**	9445	99.9	900 (756–1077)	992 (832–1210)	**11[6.0 : 15]**	-0.66[-1.3 : 0.0]	-0.29[-0.94 : 0.35]	0.436
**18–29**	1866	100	931 (778–1088)	1043 (877–1270)	28[3.8 : 58]	-6.7[-12 : -0.80]	**-11[-16 : -5.2]**	0.292
**30–39**	1903	99.9	886 (738–1058)	945 (789–1146)	46[0.38 : 111]	-4.2[-11 : 3.2]	**-12[-18 : -5.0]**	0.126
**40–49**	1920	99.9	873 (728–1044)	947 (799–1140)	0.070[-35 : 54]	2.8[-3.8 : 9.8]	4.8[-2.2 : 12]	0.686
**50–59**	1920	99.9	906 (752–1096)	1006 (850–1228)	17[-31 : 99]	4.1[-2.8 : 11]	3.9[-3.0 : 11]	0.976
**60–69**	1836	99.9	913 (775–1092)	1031 (868–1234)	-17[-67 : 113]	-0.20[-9.9 : 11]	4.8[-5.9 : 17]	0.519

BMI and age-stratified median concentrations

AGE GROUP	Normal BMI		Overweight BMI					Obese BMI				
	Males	Females	Males	Females	Males	Females	Overweight × sex	Males	Females	Males	Females	Obese × sex
	Concentration (pg/ml)	Concentration (pg/ml)	Concentration (pg/ml)	Concentration (pg/ml)	%change	%change	Interaction P	Concentration (pg/ml)	Concentration (pg/ml)	%change	%change	Interaction P
**All**	869 (727–1019)	946 (802–1134)	907 (760–1087)	1032 (872–1252)	**4.9[2.9 : 6.9]**	2.1[-0.52 : 4.8]	0.007	991 (829–1229)	1141 (941–1409)	**17[14 : 21]**	1.2[-3.5 : 6.2]	0.543
**18–29**	904 (763–1035)	1012 (851–1213)	943 (808–1124)	1071 (901–1328)	**8.8[4.2 : 14]**	0.63[-5.3 : 6.9]	0.958	1144 (947–1435)	1251 (974–1535)	**29[20 : 39]**	1.2[-9.2 : 13]	0.181
**30–39**	849 (710–997)	892 (756–1050)	889 (748–1062)	998 (817–1163)	**7.3[2.4 : 12]**	4.3[-2.0 : 11]	0.239	1005 (863–1255)	1144 (918–1384)	**24[16 : 32]**	2.8[-8.2 : 15]	0.932
**40–49**	820 (705–956)	896 (766–1049)	894 (749–1073)	1020 (846–1206)	**7.5[3.1 : 12]**	2.3[-3.2 : 8.1]	0.289	953 (805–1164)	1044 (892–1346)	**20[13 : 27]**	-0.59[-9.7 : 9.4]	0.642
**50–59**	871 (720–1040)	949 (816–1120)	920 (764–1091)	1075 (899–1291)	4.5[0.11 : 9.1]	5.1[-0.80 : 11]	0.016	976 (804–1200)	1176 (948–1522)	**14[7.5 : 22]**	5.4[-5.5 : 18]	0.066
**60–69**	898 (773–1080)	997 (848–1209)	895 (761–1089)	1026 (872–1246)	-0.56[-4.9 : 4.0]	-0.96[-6.6 : 5.0]	0.127	959 (826–1182)	1145 (964–1360)	8.3[0.93 : 16]	1.5[-9.1 : 13]	0.228

Smoking and age-stratified median concentrations

AGE GROUP	Non-smokers		Smokers		% Change in Concentration smokers vs non-smokers		
	Males	Females	Males	Females	Males	Females	Smoking × sex
	Concentration (pg/ml)	Concentration (pg/ml)	Concentration (pg/ml)	Concentration (pg/ml)	change (%)	change (%)	Interaction P
**All**	883 (741–1046)	972 (818–1172)	1037 (857–1310)	1157 (940–1425)	**21[18 : 25]**	**18[15 : 21]**	0.186
**18–29**	919 (769–1071)	1025 (865–1253)	984 (842–1231)	1142 (930–1389)	**12[6.4 : 18]**	**7.7[2.4 : 13]**	0.295
**30–39**	879 (736–1047)	941 (776–1115)	972 (776–1194)	1112 (857–1322)	**12[4.8 : 20]**	**11[3.8 : 18]**	0.787
**40–49**	859 (721–1003)	924 (782–1099)	1077 (838–1346)	1154 (956–1487)	**27[20 : 35]**	**29[22 : 37]**	0.698
**50–59**	882 (733–1069)	981 (828–1186)	1037 (880–1314)	1190 (943–1448)	**20[14 : 27]**	**20[13 : 27]**	0.91
**60–69**	883 (755–1033)	1003 (848–1205)	1212 (958–1436)	1217 (1023–1445)	**39[30 : 48]**	**22[15 : 30]**	**0.005**

Supplementary Table 35: CCL26 (Eotaxin-3, MIP-4α, IMAC, Uniprot:Q9Y258)

Sex and age-stratified median concentrations

AGE GROUP	Biomarker/cohort information		Median Concentration			Age Group (% change in concentration)		
	**N measured**	**Detection Range**	**Males**	**Females**	**Sex difference**	**Males**	**Females**	**Sex × age**
		(% in range)	(pg/ml)	(pg/ml)	F relative to M (%)	change/10 years (%)	change/10 years (%)	Interaction P
**All**	9445	99.9	22 (16–30)	18 (14–24)	**-15[-22 : -7.4]**	0.33[-1.0 : 1.7]	-0.49[-1.8 : 0.86]	0.395
**18–29**	1866	99.9	21 (16–29)	17 (13–23)	-36[-58 : -1.9]	0.78[-11 : 14]	12[-1.0 : 27]	0.231
**30–39**	1903	99.9	22 (17–29)	18 (14–24)	88[-7.9 : 282]	11[-3.4 : 29]	-12[-24 : 1.5]	0.022
**40–49**	1920	100	22 (17–30)	18 (14–25)	-16[-66 : 109]	-6.6[-19 : 7.4]	-7.2[-20 : 7.4]	0.95
**50–59**	1920	99.9	21 (16–30)	18 (13–25)	-20[-75 : 151]	-3.2[-16 : 12]	-2.5[-16 : 13]	0.951
**60–69**	1836	99.7	22 (16–31)	18 (13–24)	-53[-95 : 315]	-12[-31 : 12]	-4.5[-26 : 22]	0.645

BMI and age-stratified median concentrations

AGE GROUP	Normal BMI		Overweight BMI					Obese BMI				
	**Males**	**Females**	**Males**	**Females**	**Males**	**Females**	**Overweight × sex**	**Males**	**Females**	**Males**	**Females**	**Obese × sex**
	Concentration (pg/ml)	Concentration (pg/ml)	Concentration (pg/ml)	Concentration (pg/ml)	%change	%change	Interaction P	Concentration (pg/ml)	Concentration (pg/ml)	%change	%change	Interaction P
**All**	21 (16–29)	17 (13–24)	22 (17–30)	18 (14–24)	3.7[-0.43 : 7.9]	1.4[-3.9 : 7.1]	0.921	23 (18–32)	20 (14–26)	**13[6.8 : 20]**	2.0[-7.6 : 13]	0.282
**18–29**	20 (16–27)	17 (13–23)	22 (16–29)	18 (14–23)	7.7[-1.4 : 18]	-2.4[-14 : 10]	0.516	25 (17–31)	18 (14–24)	12[-3.5 : 30]	-14[-31 : 7.0]	0.177
**30–39**	21 (17–28)	18 (13–24)	22 (17–28)	18 (14–23)	2.1[-6.6 : 12]	-5.3[-16 : 6.5]	0.444	24 (19–32)	20 (16–27)	19[4.7 : 34]	0.030[-19 : 24]	0.22
**40–49**	21 (16–30)	18 (14–25)	22 (17–31)	18 (14–25)	2.5[-6.2 : 12]	0.58[-11 : 13]	0.87	23 (17–31)	20 (16–27)	8.0[-4.3 : 22]	5.7[-14 : 29]	0.977
**50–59**	21 (15–28)	17 (13–25)	22 (16–31)	19 (14–26)	1.2[-7.7 : 11]	8.8[-3.9 : 23]	0.453	24 (18–31)	19 (14–25)	10[-3.3 : 26]	12[-11 : 42]	0.721
**60–69**	22 (15–30)	17 (12–23)	22 (16–30)	19 (13–25)	4.1[-6.1 : 15]	9.9[-4.1 : 26]	0.258	22 (15–33)	18 (14–25)	15[-2.5 : 35]	13[-12 : 47]	0.617

Smoking and age-stratified median concentrations

AGE GROUP	Non-smokers		Smokers		% Change in Concentration smokers vs non-smokers		
	**Males**	**Females**	**Males**	**Females**	**Males**	**Females**	**Smoking × sex**
	Concentration (pg/ml)	Concentration (pg/ml)	Concentration (pg/ml)	Concentration (pg/ml)	change (%)	change (%)	Interaction P
**All**	21 (16–29)	18 (13–24)	24 (18–34)	19 (14–26)	**14[7.6 : 20]**	4.8[-0.73 : 11]	0.039
**18–29**	21 (16–28)	17 (13–23)	23 (17–33)	18 (13–24)	3.7[-6.6 : 15]	-3.6[-13 : 7.0]	0.335
**30–39**	22 (17–29)	18 (14–24)	23 (18–31)	18 (15–27)	7.8[-5.0 : 22]	2.4[-9.4 : 16]	0.567
**40–49**	21 (16–30)	18 (14–25)	24 (19–32)	20 (15–27)	**15[1.8 : 31]**	**17[3.7 : 32]**	0.888
**50–59**	21 (16–29)	18 (13–25)	24 (18–37)	18 (14–25)	**17[2.9 : 32]**	3.1[-9.2 : 17]	0.175
**60–69**	21 (16–29)	18 (13–24)	24 (17–40)	19 (14–24)	**31[13 : 52]**	9.3[-4.9 : 26]	0.084

Supplementary Table 36: CXCL10 (Interferon gamma-induced protein 10, IP10, IFI10, SCYB10, Uniprot:P02778)

Sex and age-stratified median concentrations

AGE GROUP	Biomarker/cohort information		Median Concentration			Age Group (% change in concentration)		
	N measured	Detection Range	Males	Females	Sex difference	Males	Females	Sex × age
		(% in range)	(pg/ml)	(pg/ml)	F relative to M (%)	change/10 years (%)	change/10 years (%)	Interaction P
**All**	9445	99.9	339 (266–442)	339 (264–444)	**-9.4[-15 : -3.2]**	**6.4[5.3 : 7.5]**	**8.7[7.6 : 9.8]**	**0.003**
**18–29**	1866	100	303 (242–381)	298 (225–385)	-1.7[-29 : 35]	4.8[-4.4 : 15]	3.6[-5.6 : 14]	0.853
**30–39**	1903	99.9	330 (257–424)	316 (247–409)	40[-21 : 147]	4.7[-6.6 : 17]	-5.6[-16 : 5.8]	0.207
**40–49**	1920	99.9	329 (261–419)	323 (256–411)	-3.7[-52 : 93]	4.3[-6.4 : 16]	4.8[-6.3 : 17]	0.948
**50–59**	1920	99.9	355 (283–453)	359 (289–463)	-14[-62 : 94]	7.0[-3.6 : 19]	11[-0.24 : 23]	0.649
**60–69**	1836	99.8	398 (321–529)	413 (325–535)	-52[-90 : 124]	9.4[-7.5 : 29]	24[3.6 : 47]	0.325

BMI and age-stratified median concentrations

AGE GROUP	Normal BMI		Overweight BMI					Obese BMI				
	Males	Females	Males	Females	Males	Females	Overweight × sex	Males	Females	Males	Females	Obese × sex
	Concentration (pg/ml)	Concentration (pg/ml)	Concentration (pg/ml)	Concentration (pg/ml)	%change	%change	Interaction P	Concentration (pg/ml)	Concentration (pg/ml)	%change	%change	Interaction P
**All**	323 (254–414)	320 (251–413)	348 (275–456)	353 (277–471)	**5.0[1.9 : 8.2]**	4.8[0.63 : 9.1]	0.072	378 (307–505)	394 (302–507)	**16[11 : 21]**	4.7[-2.8 : 13]	0.948
**18–29**	299 (243–371)	292 (219–366)	306 (246–401)	310 (238–395)	4.4[-2.1 : 12]	0.94[-7.9 : 11]	0.773	321 (256–415)	353 (246–460)	5.4[-5.6 : 18]	9.9[-6.8 : 30]	0.042
**30–39**	313 (248–407)	305 (235–392)	323 (255–418)	316 (248–406)	5.4[-1.8 : 13]	5.2[-4.3 : 16]	0.663	356 (285–448)	356 (290–445)	**21[9.1 : 33]**	8.6[-8.6 : 29]	0.575
**40–49**	312 (250–405)	308 (246–388)	326 (262–415)	332 (264–434)	6.0[-1.0 : 14]	0.22[-8.4 : 9.7]	0.616	370 (298–456)	359 (291–478)	**20[8.9 : 31]**	-6.6[-20 : 9.1]	0.497
**50–59**	334 (262–421)	338 (275–413)	367 (289–450)	386 (300–493)	3.7[-2.9 : 11]	5.1[-3.7 : 15]	0.076	405 (322–524)	432 (326–535)	**17[6.7 : 29]**	3.7[-12 : 22]	0.537
**60–69**	366 (292–488)	392 (309–512)	406 (330–545)	418 (330–573)	**12[3.8 : 20]**	5.6[-4.0 : 16]	0.718	468 (365–606)	476 (374–608)	**23[9.5 : 38]**	3.4[-14 : 24]	0.268

Smoking and age-stratified median concentrations

AGE GROUP	Non-smokers		Smokers		% Change in Concentration smokers vs non-smokers		
	Males	Females	Males	Females	Males	Females	Smoking × sex
	Concentration (pg/ml)	Concentration (pg/ml)	Concentration (pg/ml)	Concentration (pg/ml)	change (%)	change (%)	Interaction P
**All**	343 (272–448)	342 (268–451)	304 (235–394)	305 (239–402)	**-12[-16 : -8.4]**	**-9.2[-13 : -5.4]**	0.266
**18–29**	307 (249–390)	302 (230–383)	287 (220–361)	272 (215–397)	**-9.8[-17 : -2.4]**	-2.3[-9.6 : 5.6]	0.156
**30–39**	332 (262–424)	322 (254–413)	285 (212–390)	279 (221–354)	**-14[-23 : -5.3]**	**-16[-23 : -7.0]**	0.842
**40–49**	333 (265–427)	330 (263–419)	290 (240–388)	294 (235–359)	**-11[-19 : -2.3]**	**-9.7[-18 : -0.97]**	0.789
**50–59**	365 (289–466)	364 (290–474)	316 (240–387)	336 (277–425)	**-17[-24 : -9.8]**	-7.8[-16 : 0.81]	0.087
**60–69**	404 (325–536)	424 (332–546)	359 (295–454)	344 (282–458)	-7.9[-17 : 2.3]	**-11[-19 : -1.3]**	0.684

Supplementary Table 37: IL-8 (Interleukin-8, CXCL8, GCP-1, LECT, LUCT, LYNAP, MDNCF, Uniprot:P10145)

Sex and age-stratified median concentrations

AGE GROUP	Biomarker/cohort information		Median Concentration			Age Group (% change in concentration)		
	**N measured**	**Detection Range** (% in range)	**Males** (pg/ml)	**Females** (pg/ml)	**Sex difference** F relative to M (%)	**Males** change/10 years (%)	**Females** change/10 years (%)	**Sex × age** Interaction P
**All**	9873	100	9.3 (6.9–13)	8.9 (6.6–13)	**-14[-22 : -5.6]**	**6.2[4.7 : 7.8]**	**8.4[6.8 : 10]**	0.052
**18–29**	1994	100	8.4 (6.2–11)	8.2 (6.2–11)	-1.7[-41 : 63]	4.3[-10. : 21]	1.7[-12 : 18]	0.811
**30–39**	2010	100	8.6 (6.6–12)	8.2 (6.1–11)	84[-13 : 287]	18[1.7 : 37]	-3.6[-17 : 12]	0.06
**40–49**	2011	100	9.5 (7.0–13)	8.6 (6.3–12)	-27[-73 : 98]	-1.7[-16 : 15]	3.3[-12 : 21]	0.659
**50–59**	1986	99.9	10 (7.4–14)	9.5 (7.1–14)	-25[-78 : 157]	9.5[-6.6 : 28]	14[-2.5 : 34]	0.707
**60–69**	1872	100	11 (7.7–16)	10 (7.8–16)	-63[-96 : 243]	-4.0[-25 : 22]	13[-13 : 46]	0.37

BMI and age-stratified median concentrations

AGE GROUP	Normal BMI		Overweight BMI					Obese BMI				
	**Males** Concentration (pg/ml)	**Females** Concentration (pg/ml)	**Males** Concentration (pg/ml)	**Females** Concentration (pg/ml)	**Males** %change	**Females** %change	**Overweight × sex** Interaction P	**Males** Concentration (pg/ml)	**Females** Concentration (pg/ml)	**Males** %change	**Females** %change	**Obese × sex** Interaction P
**All**	8.8 (6.7–12)	8.8 (6.5–12)	9.7 (7.2–14)	9.1 (6.7–13)	4.6[0.11 : 9.3]	0.24[-5.5 : 6.3]	0.275	10 (7.5–15)	9.3 (6.7–13)	**14[6.4 : 21]**	3.3[-7.2 : 15]	0.094
**18–29**	8.2 (6.2–11)	8.1 (6.2–11)	8.9 (6.3–12)	8.2 (6.1–12)	2.6[-7.6 : 14]	-1.9[-15 : 13]	0.6	10 (7.5–17)	8.4 (6.6–12)	**33[11 : 59]**	1.1[-22 : 32]	0.031
**30–39**	8.2 (6.5–11)	8.0 (5.9–11)	9.0 (6.5–12)	8.3 (6.4–11)	10[0.18 : 21]	7.7[-4.9 : 22]	0.567	10 (6.8–14)	9.0 (6.4–12)	**21[6.5 : 38]**	24[-1.1 : 56]	0.781
**40–49**	9.1 (6.9–13)	8.3 (6.2–11)	9.8 (7.4–14)	9.1 (6.6–13)	4.0[-5.7 : 15]	3.9[-8.6 : 18]	0.838	10 (7.2–14)	8.4 (6.2–13)	9.1[-4.6 : 25]	-5.0[-24 : 19]	0.286
**50–59**	9.7 (7.1–14)	9.7 (7.3–14)	10 (7.4–14)	9.3 (7.1–13)	3.9[-5.9 : 15]	-13[-24 : -0.41]	0.041	11 (7.8–15)	9.6 (6.9–14)	11[-3.6 : 28]	-11[-31 : 14]	0.127
**60–69**	10 (7.6–15)	10 (7.7–15)	11 (7.8–17)	10 (7.7–15)	5.7[-4.9 : 17]	3.5[-10 : 19]	0.872	11 (7.8–16)	11 (9.0–16)	4.7[-11 : 23]	9.3[-16 : 42]	0.291

Smoking and age-stratified median concentrations

AGE GROUP	Non-smokers		Smokers		% Change in Concentration smokers vs non-smokers		
	**Males** Concentration (pg/ml)	**Females** Concentration (pg/ml)	**Males** Concentration (pg/ml)	**Females** Concentration (pg/ml)	**Males** change (%)	**Females** change (%)	**Smoking × sex** Interaction P
**All**	9.2 (6.8–13)	8.9 (6.6–12)	10 (7.6–16)	9.4 (6.9–14)	**20[13 : 28]**	**9.9[3.7 : 17]**	0.037
**18–29**	8.4 (6.2–11)	8.1 (6.2–11)	8.7 (6.6–13)	8.4 (6.4–12)	**20[5.8 : 36]**	**13[0.28 : 28]**	0.532
**30–39**	8.6 (6.5–12)	8.2 (6.0–11)	8.9 (7.0–13)	8.5 (6.4–12)	**18[3.6 : 35]**	-0.69[-13 : 13]	0.063
**40–49**	9.4 (7.0–13)	8.5 (6.2–12)	11 (8.3–16)	9.3 (6.7–13)	**21[5.6 : 39]**	8.5[-4.7 : 24]	0.25
**50–59**	9.8 (7.3–14)	9.4 (7.1–14)	11 (8.2–17)	11 (7.6–15)	**22[6.8 : 40]**	**15[0.67 : 32]**	0.56
**60–69**	10 (7.7–16)	10 (7.8–15)	13 (8.5–20)	13 (8.4–17)	**19[2.6 : 38]**	14[-1.6 : 31]	0.645

Supplementary Table 38: bFGF (Basic Fibroblast growth factor, FGF2, HBGF-2, Uniprot:P09038)

Sex and age-stratified median concentrations

AGE GROUP	Biomarker/cohort information		Median Concentration			Age Group (% change in concentration)		
	N measured	Detection Range	Males	Females	Sex difference	Males	Females	Sex × age
		(% in range)	(pg/ml)	(pg/ml)	F relative to M (%)	change/10 years (%)	change/10 years (%)	Interaction P
**All**	9866	100	77 (48–118)	76 (47–114)	-2.0[-12 : 9.2]	**5.3[3.5 : 7.1]**	**5.4[3.6 : 7.1]**	0.964
**18–29**	1991	100	69 (43–105)	72 (43–107)	2.9[-42 : 82]	3.0[-13 : 22]	2.3[-13 : 20]	0.955
**30–39**	2009	100	74 (46–109)	71 (44–108)	88[-22 : 357]	14[-4.5 : 36]	-5.9[-21 : 12]	0.133
**40–49**	2010	100	77 (48–118)	73 (45–106)	-12[-72 : 171]	4.6[-12 : 25]	6.9[-11 : 28]	0.867
**50–59**	1983	99.9	87 (53–126)	78 (50–119)	-46[-87 : 118]	3.2[-14 : 24]	14[-4.6 : 37]	0.428
**60–69**	1873	100	84 (53–134)	84 (54–131)	-49[-95 : 474]	-14[-34 : 12]	-3.9[-27 : 27]	0.568

BMI and age-stratified median concentrations

AGE GROUP	Normal BMI		Overweight BMI					Obese BMI				
	Males	Females	Males	Females	Males	Females	Overweight × sex	Males	Females	Males	Females	Obese × sex
	Concentration (pg/ml)	Concentration (pg/ml)	Concentration (pg/ml)	Concentration (pg/ml)	%change	%change	Interaction P	Concentration (pg/ml)	Concentration (pg/ml)	%change	%change	Interaction P
**All**	75 (45–113)	74 (46–109)	79 (49–121)	77 (47–120)	2.4[-2.6 : 7.6]	0.71[-5.8 : 7.7]	0.86	82 (55–131)	82 (51–126)	**12[4.5 : 21]**	3.7[-8.2 : 17]	0.62
**18–29**	68 (43–101)	72 (42–105)	69 (42–111)	71 (45–104)	-1.4[-12 : 11]	-3.7[-18 : 13]	0.932	81 (61–113)	79 (47–129)	17[-3.9 : 44]	-0.13[-26 : 34]	0.362
**30–39**	74 (43–107)	69 (44–102)	72 (46–105)	68 (41–111)	0.45[-10 : 12]	-2.0[-15 : 14]	0.749	79 (50–130)	87 (49–116)	14[-2.1 : 34]	15[-12 : 51]	0.997
**40–49**	74 (42–110)	70 (44–99)	81 (49–119)	74 (44–113)	8.1[-3.2 : 21]	2.5[-11 : 19]	0.552	85 (59–139)	81 (51–114)	**32[13 : 53]**	-3.8[-25 : 24]	0.543
**50–59**	81 (48–121)	79 (53–116)	89 (57–127)	83 (48–127)	12[0.090 : 25]	0.96[-13 : 18]	0.176	90 (52–127)	74 (48–123)	4.9[-11 : 23]	-8.9[-31 : 21]	0.182
**60–69**	89 (55–131)	77 (51–122)	81 (51–136)	91 (55–135)	-8.0[-18 : 3.0]	7.1[-8.0 : 25]	0.039	78 (57–136)	103 (69–152)	-4.5[-20 : 14]	22[-7.9 : 63]	0.02

Smoking and age-stratified median concentrations

AGE GROUP	Non-smokers		Smokers		% Change in Concentration smokers vs non-smokers		
	Males	Females	Males	Females	Males	Females	Smoking × sex
	Concentration (pg/ml)	Concentration (pg/ml)	Concentration (pg/ml)	Concentration (pg/ml)	change (%)	change (%)	Interaction P
**All**	77 (48–118)	75 (47–112)	77 (48–120)	79 (47–124)	2.8[-3.9 : 10]	5.9[-0.91 : 13]	0.547
**18–29**	69 (44–105)	72 (43–104)	67 (43–96)	75 (46–121)	2.2[-11 : 18]	7.5[-6.4 : 23]	0.616
**30–39**	75 (48–110)	70 (45–108)	68 (40–100)	69 (43–113)	-4.3[-18 : 12]	-8.7[-22 : 6.2]	0.667
**40–49**	77 (46–118)	73 (46–106)	79 (59–119)	77 (45–109)	6.2[-9.2 : 24]	3.8[-10 : 20]	0.837
**50–59**	87 (52–124)	77 (50–116)	88 (56–137)	92 (54–135)	4.8[-10 : 22]	**20[2.7 : 40]**	0.223
**60–69**	83 (53–134)	83 (54–125)	85 (60–142)	86 (59–144)	7.6[-8.5 : 27]	8.2[-7.4 : 26]	0.964

Supplementary Table 39: Flt1 (Vascular Endothelial Growth Factor Receptor 1, Uniprot:P17948)

Sex and age-stratified median concentrations

AGE GROUP	Biomarker/cohort information		Median Concentration			Age Group (% change in concentration)		
	N measured	Detection Range (% in range)	Males (pg/ml)	Females (pg/ml)	Sex difference F relative to M (%)	Males change/10 years (%)	Females change/10 years (%)	Sex × age Interaction P
**All**	9866	100	95 (82–111)	89 (74–104)	**-19[-22 : -16]**	0.060[-0.55 : 0.66]	**3.0[2.4 : 3.7]**	**9.10E-12**
**18–29**	1991	100	96 (82–112)	82 (68–96)	**-28[-40 : -12]**	-5.9[-11 : -0.36]	0.25[-5.3 : 6.1]	0.118
**30–39**	2009	100	94 (80–109)	87 (74–103)	-30[-51 : -1.8]	2.5[-4.4 : 9.9]	**11[3.5 : 19]**	0.115
**40–49**	2010	100	94 (81–111)	90 (76–106)	-0.86[-33 : 48]	2.1[-4.0 : 8.6]	0.97[-5.3 : 7.6]	0.802
**50–59**	1983	99.9	96 (83–112)	92 (77–104)	-30[-58 : 17]	-3.2[-9.4 : 3.4]	2.1[-4.4 : 9.1]	0.26
**60–69**	1873	100	96 (82–113)	93 (79–107)	-16[-64 : 95]	-2.9[-11 : 6.5]	-0.76[-10. : 9.4]	0.752

BMI and age-stratified median concentrations

AGE GROUP	Normal BMI		Overweight BMI					Obese BMI				
	Males	Females	Males	Females	Males	Females	Overweight × sex	Males	Females	Males	Females	Obese × sex
	Concentration (pg/ml)	Concentration (pg/ml)	Concentration (pg/ml)	Concentration (pg/ml)	%change	%change	Interaction P	Concentration (pg/ml)	Concentration (pg/ml)	%change	%change	Interaction P
**All**	96 (82–112)	88 (73–103)	94 (81–111)	90 (76–103)	-2.4[-4.1 : -0.61]	0.23[-2.2 : 2.7]	0.002	96 (82–113)	91 (76–109)	-0.77[-3.4 : 1.9]	0.34[-4.0 : 4.9]	0.011
**18–29**	98 (82–113)	81 (68–94)	94 (81–108)	83 (69–99)	-2.3[-6.2 : 1.8]	2.8[-2.8 : 8.8]	0.127	94 (80–111)	86 (69–98)	-3.5[-9.9 : 3.5]	7.9[-2.6 : 20]	0.037
**30–39**	95 (80–111)	88 (74–103)	92 (79–107)	86 (73–99)	-2.6[-6.8 : 1.7]	-5.5[-11 : 0.070]	0.769	95 (81–110)	89 (75–104)	0.75[-5.2 : 7.1]	-6.2[-16 : 4.3]	0.688
**40–49**	94 (81–109)	89 (75–107)	94 (81–111)	90 (76–102)	-0.68[-4.5 : 3.3]	-1.2[-6.2 : 4.0]	0.542	96 (83–116)	93 (78–110)	4.6[-0.86 : 10]	-0.66[-9.1 : 8.6]	0.89
**50–59**	96 (83–110)	91 (76–102)	95 (82–112)	93 (77–107)	-0.33[-4.3 : 3.8]	0.50[-4.9 : 6.2]	0.272	99 (84–114)	92 (79–107)	-2.1[-7.7 : 3.8]	-2.9[-12 : 7.6]	0.26
**60–69**	96 (85–112)	93 (77–107)	95 (81–113)	93 (81–106)	-1.1[-4.9 : 3.0]	1.1[-4.1 : 6.6]	0.106	94 (81–111)	95 (84–117)	-0.87[-6.9 : 5.6]	-0.62[-10 : 9.8]	0.164

Smoking and age-stratified median concentrations

AGE GROUP	Non-smokers		Smokers		% Change in Concentration smokers vs non-smokers		
	Males Concentration (pg/ml)	Females Concentration (pg/ml)	Males Concentration (pg/ml)	Females Concentration (pg/ml)	Males change (%)	Females change (%)	Smoking × sex Interaction P
**All**	95 (82–112)	89 (74–104)	94 (79–110)	88 (73–100)	-1.7[-4.1 : 0.69]	-1.5[-3.9 : 0.85]	0.906
**18–29**	97 (83–112)	82 (68–96)	92 (76–109)	79 (68–93)	**-5.3[-9.8 : -0.56]**	-3.7[-8.2 : 1.1]	0.627
**30–39**	94 (80–109)	88 (74–103)	94 (76–109)	85 (74–98)	-3.7[-9.3 : 2.3]	-2.2[-7.8 : 3.8]	0.721
**40–49**	94 (81–111)	91 (76–106)	94 (81–112)	86 (74–102)	1.6[-3.9 : 7.3]	-1.5[-6.5 : 3.8]	0.426
**50–59**	96 (83–112)	91 (77–104)	94 (79–109)	93 (75–107)	-4.0[-9.2 : 1.5]	0.99[-4.5 : 6.9]	0.21
**60–69**	95 (82–112)	93 (79–108)	97 (85–113)	91 (79–104)	4.5[-1.2 : 11]	-0.90[-6.1 : 4.6]	0.183

Supplementary Table 40: GM-CSF (Granulocyte-macrophage colony-stimulating factor, CSF2, Uniprot:P04141)

Sex and age-stratified median concentrations

AGE GROUP	Biomarker/cohort information		Median Concentration			Age Group (% change in concentration)		
	N measured	Detection Range (% in range)	Males (pg/ml)	Females (pg/ml)	Sex difference F relative to M (%)	Males change/10 years (%)	Females change/10 years (%)	Sex × age Interaction P
**All**	9872	88.2	0.16 (0.095–0.24)	0.16 (0.10–0.24)	6.0[-6.2 : 20]	-1.9[-3.7 : 0.030]	-2.2[-4.0 : -0.30]	0.819
**18–29**	1994	88.5	0.17 (0.10–0.24)	0.17 (0.11–0.26)	5.2[-44 : 97]	-11[-26 : 7.2]	-9.8[-25 : 8.1]	0.934
**30–39**	2010	88.4	0.16 (0.091–0.23)	0.16 (0.10–0.24)	17[-57 : 218]	-0.10[-18 : 22]	-3.3[-21 : 18]	0.819
**40–49**	2011	87.6	0.15 (0.095–0.23)	0.16 (0.10–0.24)	-27[-80 : 160]	-0.50[-18 : 21]	7.1[-13 : 31]	0.61
**50–59**	1985	89	0.16 (0.094–0.23)	0.16 (0.10–0.24)	-62[-92 : 80]	-4.3[-22 : 17]	15[-6.0 : 41]	0.207
**60–69**	1872	87.4	0.16 (0.094–0.24)	0.16 (0.10–0.24)	-85[-99 : 186]	-26[-46 : 1.8]	0.61[-28 : 41]	0.194

BMI and age-stratified median concentrations

AGE GROUP	Normal BMI		Overweight BMI					Obese BMI				
	Males Concentration (pg/ml)	Females Concentration (pg/ml)	Males Concentration (pg/ml)	Females Concentration (pg/ml)	Males %change	Females %change	Overweight × sex Interaction P	Males Concentration (pg/ml)	Females Concentration (pg/ml)	Males %change	Females %change	Obese × sex Interaction P
**All**	0.16 (0.094–0.23)	0.16 (0.10–0.24)	0.16 (0.096–0.24)	0.16 (0.10–0.24)	3.2[-2.4 : 9.2]	-1.9[-9.1 : 5.8]	0.497	0.16 (0.10–0.24)	0.17 (0.11–0.25)	8.1[-0.49 : 18]	-0.31[-13 : 15]	0.648
**18–29**	0.17 (0.10–0.24)	0.17 (0.10–0.27)	0.17 (0.10–0.25)	0.17 (0.11–0.25)	1.2[-11 : 15]	-17[-30 : -0.010]	0.252	0.16 (0.098–0.26)	0.17 (0.12–0.28)	6.8[-15 : 33]	-8.3[-34 : 27]	0.81
**30–39**	0.16 (0.092–0.24)	0.16 (0.097–0.24)	0.15 (0.081–0.21)	0.16 (0.11–0.25)	-4.3[-16 : 8.6]	-9.2[-23 : 7.3]	0.956	0.19 (0.12–0.27)	0.16 (0.10–0.24)	27[6.6 : 52]	-17[-39 : 12]	0.014
**40–49**	0.16 (0.099–0.22)	0.16 (0.091–0.23)	0.16 (0.091–0.25)	0.16 (0.10–0.24)	0.28[-11 : 14]	-4.2[-19 : 13]	0.649	0.15 (0.089–0.23)	0.17 (0.10–0.26)	-3.3[-19 : 15]	-11[-33 : 19]	0.244
**50–59**	0.15 (0.085–0.23)	0.16 (0.096–0.23)	0.16 (0.099–0.22)	0.17 (0.10–0.24)	4.5[-7.8 : 18]	18[-0.62 : 39]	0.838	0.16 (0.097–0.24)	0.16 (0.11–0.24)	5.0[-12 : 26]	38[0.83 : 90]	0.733
**60–69**	0.15 (0.089–0.21)	0.16 (0.10–0.24)	0.16 (0.10–0.25)	0.16 (0.10–0.23)	16[1.3 : 33]	0.95[-16 : 21]	0.178	0.16 (0.092–0.26)	0.18 (0.12–0.25)	5.2[-15 : 31]	13[-20 : 59]	0.582

Smoking and age-stratified median concentrations

AGE GROUP	Non-smokers		Smokers		% Change in Concentration smokers vs non-smokers		
	Males Concentration (pg/ml)	Females Concentration (pg/ml)	Males Concentration (pg/ml)	Females Concentration (pg/ml)	Males change (%)	Females change (%)	Smoking × sex Interaction P
**All**	0.16 (0.097–0.24)	0.16 (0.10–0.24)	0.15 (0.088–0.23)	0.16 (0.097–0.24)	-4.7[-12 : 2.8]	-4.7[-12 : 2.8]	0.99
**18–29**	0.17 (0.10–0.24)	0.17 (0.11–0.27)	0.17 (0.093–0.25)	0.16 (0.099–0.23)	-4.6[-18 : 11]	-11[-23 : 4.0]	0.551
**30–39**	0.16 (0.096–0.24)	0.16 (0.10–0.24)	0.15 (0.079–0.21)	0.16 (0.095–0.24)	-6.3[-21 : 12]	-7.7[-22 : 9.5]	0.901
**40–49**	0.16 (0.096–0.24)	0.16 (0.10–0.23)	0.15 (0.086–0.21)	0.17 (0.093–0.24)	-11[-26 : 5.7]	-3.0[-18 : 15]	0.469
**50–59**	0.16 (0.095–0.23)	0.16 (0.099–0.23)	0.14 (0.088–0.22)	0.17 (0.10–0.25)	-2.7[-18 : 15]	9.7[-7.7 : 30]	0.33
**60–69**	0.16 (0.095–0.23)	0.16 (0.10–0.24)	0.16 (0.095–0.27)	0.14 (0.097–0.21)	3.4[-15 : 26]	-13[-28 : 5.3]	0.221

Supplementary Table 41: IL-7 (Interleukin-7, Uniprot:P13232)

Sex and age-stratified median concentrations

AGE GROUP	Biomarker/cohort information		Median Concentration			Age Group (% change in concentration)		
	N measured	Detection Range	Males	Females	Sex difference	Males	Females	Sex × age
		(% in range)	(pg/ml)	(pg/ml)	F relative to M (%)	change/10 years (%)	change/10 years (%)	Interaction P
**All**	9872	100	14 (10–18)	14 (11–18)	**16[9.0 : 24]**	1.1[0.10 : 2.1]	**-1.6[-2.6 : -0.66]**	**9.39E-05**
**18–29**	1994	99.9	13 (9.8–17)	14 (11–19)	9.7[-23 : 57]	-3.7[-13 : 6.8]	-3.5[-13 : 6.9]	0.976
**30–39**	2010	100	13 (10–17)	14 (11–18)	83[9.6 : 206]	4.4[-5.9 : 16]	-11[-20 : -1.1]	0.036
**40–49**	2011	100	14 (11–18)	14 (10–18)	-8.7[-52 : 75]	-6.5[-15 : 3.4]	-4.5[-14 : 6.0]	0.774
**50–59**	1985	100	14 (10–19)	14 (11–18)	-24[-65 : 65]	-4.7[-14 : 5.3]	-0.11[-9.6 : 10]	0.512
**60–69**	1872	99.9	14 (11–18)	14 (11–18)	-62[-91 : 53]	-4.7[-18 : 11]	11[-5.4 : 30]	0.174

BMI and age-stratified median concentrations

AGE GROUP	Normal BMI		Overweight BMI					Obese BMI				
	Males	Females	Males	Females	Males	Females	Overweight × sex	Males	Females	Males	Females	Obese × sex
	Concentration (pg/ml)	Concentration (pg/ml)	Concentration (pg/ml)	Concentration (pg/ml)	%change	%change	Interaction P	Concentration (pg/ml)	Concentration (pg/ml)	%change	%change	Interaction P
**All**	13 (10.–17)	14 (10–18)	14 (10–18)	14 (11–19)	4.0[1.0 : 7.1]	2.8[-1.2 : 6.8]	0.388	15 (11–20)	16 (12–20)	**13[8.7 : 18]**	4.7[-2.5 : 12]	0.836
**18–29**	13 (9.6–17)	14 (11–19)	14 (10–18)	14 (11–20)	4.7[-2.7 : 13]	4.1[-6.1 : 15]	0.812	15 (11–19)	16 (12–21)	16[2.0 : 31]	14[-5.1 : 38]	0.744
**30–39**	13 (9.9–16)	14 (10–17)	13 (9.9–18)	15 (11–19)	2.4[-4.0 : 9.3]	5.1[-3.6 : 14]	0.346	14 (11–19)	16 (12–21)	**14[4.5 : 25]**	10[-5.9 : 29]	0.798
**40–49**	13 (10–17)	14 (10–17)	15 (11–19)	14 (10–19)	**11[4.1 : 18]**	-2.4[-10 : 6.2]	0.468	15 (12–21)	15 (11–19)	**18[7.8 : 29]**	-8.5[-21 : 5.9]	0.748
**50–59**	13 (10–19)	13 (10–17)	14 (10–18)	14 (11–19)	0.11[-5.9 : 6.5]	6.2[-2.3 : 16]	0.083	16 (12–21)	15 (13–20)	**14[4.7 : 25]**	10[-5.6 : 29]	0.905
**60–69**	14 (11–18)	14 (11–18)	14 (11–19)	15 (12–19)	-0.81[-7.1 : 5.9]	8.0[-1.1 : 18]	0.061	15 (11–19)	16 (12–20)	2.3[-7.8 : 13]	7.1[-9.2 : 26]	0.446

Smoking and age-stratified median concentrations

AGE GROUP	Non-smokers		Smokers		% Change in Concentration smokers vs non-smokers		
	Males	Females	Males	Females	Males	Females	Smoking × sex
	Concentration (pg/ml)	Concentration (pg/ml)	Concentration (pg/ml)	Concentration (pg/ml)	change (%)	change (%)	Interaction P
**All**	14 (10–18)	14 (11–19)	14 (10–18)	14 (10–18)	-0.37[-4.2 : 3.7]	-1.3[-5.1 : 2.5]	0.724
**18–29**	13 (9.8–17)	15 (11–19)	13 (10–17)	14 (11–20)	2.3[-6.3 : 12]	0.28[-8.1 : 9.4]	0.753
**30–39**	14 (10–17)	14 (11–18)	13 (9.3–16)	14 (9.7–19)	-2.9[-11 : 6.2]	-3.2[-11 : 5.8]	0.972
**40–49**	14 (11–18)	14 (10–18)	14 (11–18)	13 (10.–17)	3.2[-5.6 : 13]	-4.9[-13 : 3.6]	0.193
**50–59**	14 (10–19)	14 (10–19)	13 (10–18)	13 (11–17)	-4.3[-12 : 4.1]	-0.41[-8.6 : 8.5]	0.51
**60–69**	14 (11–18)	14 (11–18)	14 (11–19)	14 (11–18)	1.9[-7.2 : 12]	0.18[-8.4 : 9.6]	0.8

Supplementary Table 42: PlGF (Placental growth factor, PGF, Uniprot:P49763)

Sex and age-stratified median concentrations

AGE GROUP	Biomarker/cohort information		Median Concentration			Age Group (% change in concentration)		
	N measured	Detection Range (% in range)	Males (pg/ml)	Females (pg/ml)	Sex difference F relative to M (%)	Males change/10 years (%)	Females change/10 years (%)	Sex × age Interaction P
**All**	9866	100	8.2 (7.1–9.5)	7.8 (6.8–9.0)	**-10[-13 : -7.3]**	**5.4[4.9 : 6.0]**	**6.7[6.2 : 7.3]**	**4.42E-04**
**18–29**	1991	99.9	7.5 (6.6–8.6)	7.2 (6.3–8.1)	-8.0[-22 : 8.5]	-2.0[-6.6 : 2.8]	-0.66[-5.2 : 4.2]	0.684
**30–39**	2009	99.9	7.8 (6.8–8.9)	7.2 (6.4–8.1)	15[-13 : 51]	5.4[-0.23 : 11]	-0.96[-6.3 : 4.6]	0.117
**40–49**	2010	100	8.2 (7.2–9.4)	7.5 (6.6–8.6)	**-37[-54 : -14]**	2.4[-2.4 : 7.4]	**11[5.7 : 17]**	0.021
**50–59**	1983	99.9	8.6 (7.5–9.8)	8.3 (7.2–9.5)	**-51[-69 : -24]**	3.8[-2.0 : 9.8]	**18[11 : 25]**	**0.002**
**60–69**	1873	100	9.3 (8.1–11)	9.1 (8.0–10)	-0.60[-49 : 93]	**15[7.3 : 24]**	**15[6.7 : 24]**	0.977

BMI and age-stratified median concentrations

AGE GROUP	Normal BMI		Overweight BMI					Obese BMI				
	Males Concentration (pg/ml)	Females Concentration (pg/ml)	Males Concentration (pg/ml)	Females Concentration (pg/ml)	Males %change	Females %change	Overweight × sex Interaction P	Males Concentration (pg/ml)	Females Concentration (pg/ml)	Males %change	Females %change	Obese × sex Interaction P
**All**	8.0 (6.9–9.2)	7.6 (6.6–8.7)	8.4 (7.3–9.7)	8.0 (6.9–9.4)	**2.3[0.81 : 3.8]**	1.6[-0.39 : 3.6]	0.168	9.0 (7.8–10)	8.1 (7.1–9.3)	**8.6[6.2 : 11]**	0.73[-2.8 : 4.4]	0.152
**18–29**	7.4 (6.6–8.5)	7.2 (6.3–8.1)	7.7 (6.5–8.7)	7.2 (6.2–8.1)	4.4[0.94 : 7.9]	-2.6[-7.0 : 2.1]	0.126	7.9 (6.7–8.9)	7.7 (6.7–8.6)	4.8[-1.0 : 11]	0.37[-7.8 : 9.3]	0.48
**30–39**	7.7 (6.7–8.7)	7.0 (6.2–8.0)	7.8 (6.8–8.8)	7.3 (6.5–8.1)	2.0[-1.5 : 5.6]	3.8[-0.89 : 8.6]	0.268	8.2 (7.3–9.6)	7.8 (6.8–8.6)	**9.8[4.6 : 15]**	5.4[-3.0 : 15]	0.783
**40–49**	8.0 (7.0–9.2)	7.3 (6.5–8.4)	8.1 (7.2–9.3)	7.6 (6.8–8.8)	1.8[-1.3 : 4.9]	1.6[-2.4 : 5.8]	0.407	9.0 (8.0–10.)	8.0 (7.0–9.0)	**11[6.5 : 16]**	3.6[-3.4 : 11]	0.524
**50–59**	8.2 (7.2–9.4)	8.1 (7.1–9.4)	8.6 (7.6–9.9)	8.4 (7.5–9.7)	5.2[1.5 : 9.0]	0.80[-3.9 : 5.7]	0.626	9.1 (8.0–10)	8.3 (7.4–9.3)	**9.0[3.5 : 15]**	-9.7[-17 : -1.2]	0.108
**60–69**	9.2 (8.0–11)	8.9 (7.8–10)	9.3 (8.0–11)	9.3 (8.3–11)	2.8[-0.40 : 6.0]	0.91[-3.2 : 5.2]	0.4	10 (8.9–12)	9.8 (8.3–11)	**12[7.0 : 18]**	0.70[-6.9 : 8.9]	0.499

Smoking and age-stratified median concentrations

AGE GROUP	Non-smokers		Smokers		% Change in Concentration smokers vs non-smokers		
	Males Concentration (pg/ml)	Females Concentration (pg/ml)	Males Concentration (pg/ml)	Females Concentration (pg/ml)	Males change (%)	Females change (%)	Smoking × sex Interaction P
**All**	8.2 (7.1–9.5)	7.8 (6.7–8.9)	8.4 (7.3–9.9)	8.0 (6.8–9.3)	**4.7[2.6 : 6.8]**	**3.5[1.5 : 5.6]**	0.426
**18–29**	7.5 (6.6–8.6)	7.2 (6.3–8.1)	7.7 (6.5–8.9)	7.5 (6.4–8.4)	-0.62[-4.6 : 3.5]	3.2[-0.81 : 7.4]	0.19
**30–39**	7.8 (6.8–8.9)	7.1 (6.3–8.0)	8.0 (6.9–9.0)	7.5 (6.6–8.7)	2.6[-2.2 : 7.6]	3.5[-1.2 : 8.5]	0.794
**40–49**	8.1 (7.2–9.3)	7.4 (6.6–8.5)	8.5 (7.7–10)	7.9 (6.6–9.0)	**9.7[5.0 : 15]**	3.7[-0.44 : 8.0]	0.065
**50–59**	8.5 (7.4–9.8)	8.2 (7.2–9.5)	8.8 (8.0–10)	8.6 (7.5–9.9)	4.1[-0.80 : 9.3]	3.9[-1.1 : 9.1]	0.955
**60–69**	9.3 (8.0–11)	9.1 (8.0–10)	9.7 (8.5–11)	9.3 (7.8–11)	**9.3[4.5 : 14]**	2.7[-1.6 : 7.2]	0.047

Supplementary Table 43: sICAM1 (soluble intracellular adhesion molecule 1, CD54, BB2, Uniprot:Q99930/P05362)

Sex and age-stratified median concentrations

AGE GROUP	Biomarker/cohort information		Median Concentration			Age Group (% change in concentration)		
	**N measured**	**Detection Range**	**Males**	**Females**	**Sex difference**	**Males**	**Females**	**Sex × age**
		(% in range)	(ng/ml)	(ng/ml)	F relative to M (%)	change/10 years (%)	change/10 years (%)	Interaction P
**All**	9503	100	537 (469–623)	535 (463–627)	**-12[-16 : -8.8]**	-0.24[-0.85 : 0.38]	**2.7[2.0 : 3.3]**	**5.88E-11**
**18–29**	1879	100	536 (466–612)	524 (458–606)	3.7[-12 : 23]	-5.3[-9.8 : -0.60]	**-7.6[-12 : -3.0]**	0.488
**30–39**	1920	100	536 (475–624)	512 (445–603)	-0.12[-23 : 30]	-3.7[-8.7 : 1.6]	-4.9[-9.8 : 0.31]	0.746
**40–49**	1939	100	533 (465–616)	508 (440–589)	-10[-48 : 55]	-0.76[-8.8 : 7.9]	0.30[-8.1 : 9.5]	0.864
**50–59**	1928	100	540 (471–628)	549 (477–651)	**-50[-67 : -22]**	-0.87[-6.3 : 4.9]	**13[6.9 : 20]**	**0.001**
**60–69**	1837	99.9	543 (468–633)	576 (509–678)	2.7[-60 : 166]	1.9[-8.2 : 13]	2.7[-7.9 : 15]	0.915

BMI and age-stratified median concentrations

AGE GROUP	Normal BMI		Overweight BMI					Obese BMI				
	**Males**	**Females**	**Males**	**Females**	**Males**	**Females**	**Overweight × sex**	**Males**	**Females**	**Males**	**Females**	**Obese × sex**
	Concentration (ng/ml)	Concentration (ng/ml)	Concentration (ng/ml)	Concentration (ng/ml)	%change	%change	Interaction P	Concentration (ng/ml)	Concentration (ng/ml)	%change	%change	Interaction P
**All**	522 (459–600)	513 (448–594)	541 (472–625)	545 (476–636)	**3.8[2.0 : 5.8]**	0.96[-1.5 : 3.4]	0.408	597 (519–698)	592 (514–708)	**15[12 : 18]**	4.2[-0.32 : 9.0]	0.903
**18–29**	524 (464–593)	509 (451–578)	544 (471–627)	543 (468–613)	4.5[0.91 : 8.2]	-0.78[-5.4 : 4.0]	0.689	609 (539–687)	606 (529–708)	**17[10 : 24]**	3.8[-4.9 : 13]	0.565
**30–39**	525 (464–606)	488 (429–565)	534 (472–604)	512 (451–605)	0.10[-3.2 : 3.5]	2.9[-1.5 : 7.5]	0.045	612 (536–712)	588 (505–710)	**18[13 : 24]**	**15[6.5 : 25]**	0.285
**40–49**	505 (451–583)	484 (423–555)	534 (470–614)	514 (455–594)	6.0[0.51 : 12]	-2.1[-8.8 : 5.1]	0.451	596 (519–702)	548 (485–683)	**20[11 : 29]**	0.11[-11 : 13]	0.278
**50–59**	526 (459–605)	528 (457–613)	542 (474–631)	584 (503–676)	5.0[1.4 : 8.8]	1.6[-3.1 : 6.5]	0.279	589 (519–683)	600 (510–727)	**12[6.5 : 18]**	-0.88[-9.4 : 8.4]	0.632
**60–69**	525 (456–616)	565 (496–661)	556 (479–638)	569 (513–667)	**7.8[3.1 : 13]**	-0.33[-6.1 : 5.8]	0.147	569 (500–690)	621 (555–735)	**12[4.1 : 20]**	4.8[-6.2 : 17]	0.845

Smoking and age-stratified median concentrations

AGE GROUP	Non-smokers		Smokers		% Change in Concentration smokers vs non-smokers		
	**Males**	**Females**	**Males**	**Females**	**Males**	**Females**	**Smoking × sex**
	Concentration (ng/ml)	Concentration (ng/ml)	Concentration (ng/ml)	Concentration (ng/ml)	change (%)	change (%)	Interaction P
**All**	534 (466–614)	527 (457–611)	580 (501–692)	595 (510–710)	**9.7[7.0 : 12]**	**12[9.6 : 15]**	0.189
**18–29**	533 (464–611)	516 (453–590)	554 (489–635)	550 (493–623)	**5.2[0.91 : 9.6]**	**4.3[0.23 : 8.6]**	0.79
**30–39**	536 (473–619)	504 (442–592)	549 (495–642)	561 (482–645)	3.0[-1.6 : 7.9]	**11[5.9 : 16]**	0.027
**40–49**	524 (461–605)	498 (435–569)	590 (506–704)	585 (494–731)	**15[6.5 : 24]**	**11[3.3 : 19]**	0.533
**50–59**	533 (466–615)	541 (472–633)	611 (523–710)	655 (540–778)	**14[8.8 : 20]**	**17[11 : 23]**	0.484
**60–69**	538 (464–619)	563 (500–649)	633 (520–734)	677 (593–794)	**11[4.3 : 19]**	**20[13 : 28]**	0.089

Supplementary Table 44: sVCAM1 (soluble Vascular cell adhesion protein 1, CD106, INCAM-100, Uniprot:P19320)

Sex and age-stratified median concentrations

AGE GROUP	Biomarker/cohort information		Median Concentration			Age Group (% change in concentration)		
	N measured	Detection Range (% in range)	Males (ng/ml)	Females (ng/ml)	Sex difference F relative to M (%)	Males change/10 years (%)	Females change/10 years (%)	Sex × age Interaction P
**All**	9503	100	635 (564–737)	612 (534–700)	**-17[-20 : -15]**	**-1.8[-2.4 : -1.3]**	**1.2[0.71 : 1.8]**	**8.18E-17**
**18–29**	1879	100	660 (584–762)	598 (513–687)	-15[-28 : -0.11]	-2.8[-7.3 : 2.0]	-1.0[-5.6 : 3.8]	0.594
**30–39**	1920	100	651 (568–752)	614 (530–715)	-14[-33 : 11]	-0.34[-5.3 : 4.8]	1.9[-3.1 : 7.2]	0.535
**40–49**	1939	99.9	626 (554–713)	600 (523–676)	-5.6[-37 : 42]	-2.5[-8.4 : 3.8]	-2.9[-9.1 : 3.6]	0.926
**50–59**	1928	100	623 (543–723)	616 (542–700)	-31[-53 : 1.7]	-0.99[-5.8 : 4.1]	5.8[0.58 : 11]	0.068
**60–69**	1837	99.9	632 (563–726)	627 (559–721)	1.4[-55 : 127]	5.2[-3.6 : 15]	5.0[-4.2 : 15]	0.978

BMI and age-stratified median concentrations

AGE GROUP	Normal BMI		Overweight BMI					Obese BMI				
	Males Concentration (ng/ml)	Females Concentration (ng/ml)	Males Concentration (ng/ml)	Females Concentration (ng/ml)	Males %change	Females %change	Overweight × sex Interaction P	Males Concentration (ng/ml)	Females Concentration (ng/ml)	Males %change	Females %change	Obese × sex Interaction P
**All**	637 (565–741)	605 (528–692)	630 (561–729)	611 (536–700)	-0.79[-2.3 : 0.75]	1.4[-0.66 : 3.5]	0.053	651 (569–745)	632 (553–720)	1.0[-1.2 : 3.3]	4.2[0.34 : 8.3]	0.051
**18–29**	662 (583–764)	589 (510–684)	649 (590–767)	599 (514–685)	-0.26[-3.6 : 3.2]	3.3[-1.4 : 8.2]	0.473	669 (595–745)	627 (561–691)	-1.2[-6.7 : 4.6]	10[1.3 : 20]	0.068
**30–39**	657 (576–756)	606 (521–707)	630 (559–728)	604 (529–705)	-4.0[-7.0 : -0.86]	1.3[-2.9 : 5.6]	0.037	674 (564–766)	649 (554–724)	0.96[-3.4 : 5.5]	7.7[-0.24 : 16]	0.077
**40–49**	625 (546–705)	605 (524–677)	627 (561–715)	587 (519–671)	1.8[-2.2 : 5.9]	-3.0[-8.0 : 2.2]	0.187	641 (565–718)	600 (522–665)	3.4[-2.0 : 9.1]	-4.7[-13 : 4.3]	0.118
**50–59**	614 (536–720)	607 (542–682)	622 (544–722)	622 (538–706)	0.49[-2.6 : 3.7]	0.24[-3.9 : 4.6]	0.519	633 (564–717)	647 (566–725)	1.5[-3.0 : 6.2]	0.83[-6.9 : 9.1]	0.309
**60–69**	629 (560–713)	622 (547–706)	631 (569–723)	630 (567–719)	2.8[-1.1 : 6.8]	0.85[-4.1 : 6.0]	0.812	646 (572–761)	677 (593–780)	4.7[-1.4 : 11]	6.0[-3.5 : 16]	0.318

Smoking and age-stratified median concentrations

AGE GROUP	Non-smokers		Smokers		% Change in Concentration smokers vs non-smokers		
	Males Concentration (ng/ml)	Females Concentration (ng/ml)	Males Concentration (ng/ml)	Females Concentration (ng/ml)	Males change (%)	Females change (%)	Smoking × sex Interaction P
**All**	637 (568–740)	614 (538–705)	624 (539–717)	593 (512–669)	**-4.2[-6.2 : -2.1]**	**-5.6[-7.6 : -3.6]**	0.31
**18–29**	662 (587–765)	598 (513–690)	661 (580–750)	592 (506–669)	-1.1[-5.0 : 3.0]	-2.5[-6.3 : 1.4]	0.614
**30–39**	652 (570–754)	616 (534–724)	629 (558–723)	598 (513–659)	**-4.5[-8.7 : -0.21]**	**-6.7[-11 : -2.5]**	0.475
**40–49**	627 (560–715)	602 (524–676)	619 (528–688)	587 (501–652)	-4.6[-9.8 : 0.93]	**-7.2[-12 : -2.1]**	0.479
**50–59**	625 (551–727)	619 (546–700)	582 (517–689)	593 (518–699)	**-6.2[-10 : -2.1]**	-3.0[-7.0 : 1.3]	0.276
**60–69**	634 (568–729)	634 (565–727)	622 (539–693)	600 (527–660)	**-6.5[-11 : -1.3]**	**-7.8[-12 : -2.9]**	0.723

Supplementary Table 45: TIE2 (TEK tyrosine kinase, CD202B, CMCM, Uniprot:Q02763)

Sex and age-stratified median concentrations

AGE GROUP	Biomarker/cohort information		Median Concentration			Age Group (% change in concentration)		
	N measured	Detection Range	Males	Females	Sex difference	Males	Females	Sex × age
		(% in range)	(pg/ml)	(pg/ml)	F relative to M (%)	change/10 years (%)	change/10 years (%)	Interaction P
**All**	9866	100	5409 (4792–6084)	5084 (4449–5702)	**-21[-24 : -18]**	**-2.4[-2.9 : -1.9]**	**1.2[0.74 : 1.8]**	**6.62E-24**
**18–29**	1991	100	5666 (5074–6307)	5012 (4373–5666)	-20[-32 : -5.3]	-1.8[-6.4 : 3.0]	2.0[-2.7 : 6.9]	0.271
**30–39**	2009	100	5553 (4963–6176)	5045 (4373–5618)	-16[-37 : 13]	0.79[-5.0 : 6.9]	2.4[-3.5 : 8.5]	0.719
**40–49**	2010	100	5368 (4718–6063)	4964 (4361–5482)	-27[-47 : 0.92]	-3.0[-7.7 : 2.0]	1.8[-3.3 : 7.2]	0.186
**50–59**	1983	99.9	5263 (4650–5943)	5205 (4528–5843)	**-53[-70 : -25]**	-5.8[-11 : -0.070]	7.5[1.3 : 14]	**0.002**
**60–69**	1873	100	5180 (4570–5846)	5214 (4652–5831)	6.8[-45 : 107]	-7.8[-14 : -0.89]	-8.8[-15 : -1.5]	0.844

BMI and age-stratified median concentrations

AGE GROUP	Normal BMI		Overweight BMI					Obese BMI				
	Males	Females	Males	Females	Males	Females	Overweight × sex	Males	Females	Males	Females	Obese × sex
	Concentration (pg/ml)	Concentration (pg/ml)	Concentration (pg/ml)	Concentration (pg/ml)	%change	%change	Interaction P	Concentration (pg/ml)	Concentration (pg/ml)	%change	%change	Interaction P
**All**	5472 (4872–6143)	5092 (4453–5740)	5342 (4693–6013)	5114 (4472–5716)	-2.2[-3.6 : -0.69]	1.1[-0.96 : 3.2]	0.032	5402 (4840–6088)	4988 (4397–5537)	-0.44[-2.6 : 1.8]	0.060[-3.6 : 3.8]	0.292
**18–29**	5721 (5082–6360)	5000 (4405–5666)	5663 (5004–6264)	5042 (4308–5667)	-0.51[-3.8 : 2.9]	2.2[-2.5 : 7.1]	0.467	5514 (5104–6224)	4951 (4270–5516)	-1.2[-6.7 : 4.6]	2.0[-6.3 : 11]	0.75
**30–39**	5618 (5005–6224)	5082 (4377–5684)	5450 (4949–6094)	5032 (4450–5624)	-2.5[-6.0 : 1.2]	0.49[-4.3 : 5.5]	0.471	5556 (4862–6112)	4851 (4152–5406)	-0.78[-5.8 : 4.5]	-2.1[-10 : 7.0]	0.254
**40–49**	5373 (4814–6084)	4968 (4362–5464)	5336 (4612–5951)	5049 (4434–5586)	-2.1[-5.1 : 1.0]	2.5[-1.7 : 6.9]	0.094	5498 (4970–6220)	4902 (4390–5363)	3.8[-0.61 : 8.4]	0.87[-6.1 : 8.4]	0.142
**50–59**	5297 (4630–5967)	5223 (4551–5840)	5238 (4672–5907)	5205 (4527–5849)	-0.35[-4.0 : 3.4]	-1.3[-6.2 : 3.7]	0.514	5271 (4637–6007)	5163 (4493–5751)	-1.7[-6.8 : 3.7]	-1.5[-10 : 8.2]	0.692
**60–69**	5201 (4641–5909)	5241 (4648–5910)	5152 (4536–5859)	5167 (4632–5774)	-0.62[-3.7 : 2.5]	-2.6[-6.5 : 1.6]	0.74	5194 (4624–5803)	5166 (4660–5630)	1.2[-3.7 : 6.3]	-2.4[-9.7 : 5.6]	0.833

Smoking and age-stratified median concentrations

AGE GROUP	Non-smokers		Smokers		% Change in Concentration smokers vs non-smokers		
	Males	Females	Males	Females	Males	Females	Smoking × sex
	Concentration (pg/ml)	Concentration (pg/ml)	Concentration (pg/ml)	Concentration (pg/ml)	change (%)	change (%)	Interaction P
**All**	5427 (4812–6100)	5113 (4477–5729)	5286 (4676–5975)	4941 (4335–5598)	**-3.3[-5.3 : -1.3]**	**-3.4[-5.3 : -1.4]**	0.977
**18–29**	5684 (5060–6307)	5015 (4369–5685)	5598 (5045–6399)	4983 (4483–5595)	-1.5[-5.4 : 2.6]	-1.2[-5.1 : 2.8]	0.929
**30–39**	5555 (4976–6184)	5102 (4428–5654)	5517 (4955–6151)	4674 (4134–5326)	-2.3[-7.2 : 2.8]	**-9.4[-14 : -4.8]**	0.036
**40–49**	5379 (4772–6086)	5002 (4401–5499)	5238 (4581–5960)	4904 (4327–5413)	-1.4[-5.7 : 3.1]	0.050[-4.1 : 4.4]	0.632
**50–59**	5340 (4694–6018)	5207 (4541–5847)	4948 (4310–5459)	5165 (4490–5798)	**-9.3[-14 : -4.6]**	-0.95[-5.9 : 4.3]	0.016
**60–69**	5189 (4606–5863)	5232 (4675–5875)	5177 (4379–5605)	5030 (4262–5749)	-2.0[-6.3 : 2.4]	**-5.0[-9.0 : -0.90]**	0.319

Supplementary Table 46: TSLP (thymic stromal lymphopoietin, Uniprot:Q969D9)

Sex and age-stratified median concentrations

AGE GROUP	Biomarker/cohort information		Median Concentration			Age Group (% change in concentration)		
	N measured	Detection Range (% in range)	Males (pg/ml)	Females (pg/ml)	Sex difference F relative to M (%)	Males change/10 years (%)	Females change/10 years (%)	Sex × age Interaction P
**All**	9808	87.6	0.42 (0.26–0.64)	0.35 (0.20–0.55)	**-30[-38 : -20]**	**13[11 : 16]**	**17[15 : 19]**	0.02
**18–29**	1983	81.7	0.33 (0.18–0.53)	0.27 (0.15–0.45)	18[-42 : 138]	16[-5.4 : 42]	-0.76[-19 : 21]	0.279
**30–39**	2000	84	0.38 (0.22–0.58)	0.29 (0.16–0.48)	-47[-82 : 60]	-2.0[-22 : 22]	7.7[-14 : 35]	0.555
**40–49**	1994	87.6	0.41 (0.26–0.64)	0.35 (0.20–0.51)	21[-68 : 356]	18[-4.0 : 45]	6.9[-14 : 32]	0.517
**50–59**	1978	91.6	0.46 (0.32–0.69)	0.40 (0.26–0.60)	-36[-86 : 181]	-1.4[-19 : 19]	4.3[-14 : 27]	0.682
**60–69**	1853	93.6	0.53 (0.36–0.78)	0.47 (0.30–0.68)	-87[-99 : 63]	4.8[-20 : 38]	41[5.8 : 89]	0.143

BMI and age-stratified median concentrations

AGE GROUP	Normal BMI		Overweight BMI					Obese BMI				
	Males Concentration (pg/ml)	Females Concentration (pg/ml)	Males Concentration (pg/ml)	Females Concentration (pg/ml)	Males %change	Females %change	Overweight × sex Interaction P	Males Concentration (pg/ml)	Females Concentration (pg/ml)	Males %change	Females %change	Obese × sex Interaction P
**All**	0.38 (0.22–0.58)	0.33 (0.18–0.52)	0.44 (0.28–0.66)	0.38 (0.21–0.57)	**12[5.8 : 19]**	-1.5[-8.8 : 6.4]	0.636	0.55 (0.36–0.82)	0.42 (0.27–0.66)	**47[35 : 60]**	4.6[-9.2 : 21]	0.197
**18–29**	0.30 (0.17–0.47)	0.27 (0.15–0.43)	0.37 (0.18–0.55)	0.27 (0.15–0.46)	17[1.7 : 36]	-16[-32 : 1.9]	0.099	0.57 (0.32–0.88)	0.36 (0.21–0.57)	**88[47 : 140]**	-2.5[-32 : 40]	0.095
**30–39**	0.34 (0.19–0.52)	0.25 (0.14–0.45)	0.38 (0.22–0.57)	0.28 (0.17–0.49)	16[0.76 : 33]	7.4[-11 : 29]	0.764	0.52 (0.33–0.77)	0.38 (0.24–0.60)	**78[47 : 117]**	29[-7.7 : 81]	0.761
**40–49**	0.38 (0.23–0.57)	0.32 (0.19–0.49)	0.43 (0.26–0.65)	0.34 (0.19–0.51)	12[-1.7 : 28]	-4.9[-20 : 13]	0.411	0.53 (0.35–0.74)	0.40 (0.26–0.59)	**43[19 : 71]**	2.4[-24 : 38]	0.309
**50–59**	0.44 (0.30–0.68)	0.38 (0.24–0.59)	0.45 (0.32–0.67)	0.42 (0.28–0.59)	2.2[-9.4 : 15]	3.2[-12 : 21]	0.355	0.54 (0.37–0.79)	0.50 (0.33–0.74)	21[1.7 : 43]	7.5[-21 : 45]	0.711
**60–69**	0.49 (0.31–0.71)	0.45 (0.27–0.63)	0.53 (0.38–0.79)	0.48 (0.32–0.70)	19[5.3 : 34]	4.6[-11 : 22]	0.915	0.65 (0.42–0.92)	0.60 (0.37–0.84)	**39[15 : 67]**	0.56[-25 : 36]	0.701

Smoking and age-stratified median concentrations

AGE GROUP	Non-smokers		Smokers		% Change in Concentration smokers vs non-smokers		
	Males Concentration (pg/ml)	Females Concentration (pg/ml)	Males Concentration (pg/ml)	Females Concentration (pg/ml)	Males change (%)	Females change (%)	Smoking × sex Interaction P
**All**	0.41 (0.25–0.62)	0.35 (0.20–0.55)	0.49 (0.29–0.75)	0.38 (0.21–0.57)	**20[11 : 30]**	**14[5.7 : 23]**	0.367
**18–29**	0.32 (0.17–0.50)	0.27 (0.15–0.44)	0.38 (0.22–0.59)	0.29 (0.18–0.47)	17[-1.6 : 39]	12[-5.3 : 33]	0.748
**30–39**	0.38 (0.21–0.57)	0.28 (0.16–0.48)	0.39 (0.23–0.61)	0.31 (0.18–0.49)	8.5[-11 : 32]	13[-6.7 : 36]	0.777
**40–49**	0.41 (0.25–0.62)	0.34 (0.20–0.50)	0.51 (0.33–0.78)	0.36 (0.21–0.57)	**32[9.4 : 58]**	**23[3.7 : 47]**	0.621
**50–59**	0.45 (0.30–0.66)	0.40 (0.26–0.62)	0.53 (0.37–0.79)	0.45 (0.27–0.59)	**27[7.9 : 49]**	6.6[-9.6 : 26]	0.138
**60–69**	0.51 (0.36–0.76)	0.47 (0.29–0.69)	0.66 (0.44–0.92)	0.50 (0.33–0.72)	16[-2.4 : 37]	13[-3.8 : 33]	0.858

Supplementary Table 47: VEGF-A (Vascular endothelial growth factor A, MVCD1, VPF, Uniprot:P15692)

Sex and age-stratified median concentrations

AGE GROUP	Biomarker/cohort information		Median Concentration			Age Group (% change in concentration)		
	N measured	Detection Range (% in range)	Males (pg/ml)	Females (pg/ml)	Sex difference F relative to M (%)	Males change/10 years (%)	Females change/10 years (%)	Sex × age Interaction P
**All**	9866	100	241 (129–408)	249 (131–436)	14[1.3 : 27]	**9.8[7.8 : 12]**	**8.0[6.1 : 9.9]**	0.197
**18–29**	1991	100	190 (100–329)	230 (119–404)	-4.3[-48 : 76]	-8.0[-23 : 9.9]	0.88[-15 : 20]	0.465
**30–39**	2009	100	223 (120–375)	228 (115–384)	57[-41 : 317]	20[-1.6 : 46]	5.2[-13 : 28]	0.364
**40–49**	2010	100	243 (131–421)	241 (123–395)	-36[-80 : 106]	5.4[-12 : 26]	16[-4.0 : 39]	0.48
**50–59**	1983	99.9	259 (144–450)	267 (147–476)	-74[-94 : 14]	-6.3[-23 : 13]	21[-0.32 : 46]	0.066
**60–69**	1873	99.9	279 (158–473)	317 (173–517)	6.5[-91 : 1203]	3.6[-21 : 36]	3.8[-22 : 38]	0.99

BMI and age-stratified median concentrations

AGE GROUP	Normal BMI		Overweight BMI					Obese BMI				
	Males Concentration (pg/ml)	Females Concentration (pg/ml)	Males Concentration (pg/ml)	Females Concentration (pg/ml)	Males %change	Females %change	Overweight × sex Interaction P	Males Concentration (pg/ml)	Females Concentration (pg/ml)	Males %change	Females %change	Obese × sex Interaction P
**All**	222 (116–383)	241 (124–413)	246 (137–427)	265 (145–455)	**8.7[3.2 : 15]**	2.0[-5.0 : 9.5]	0.923	292 (158–485)	269 (145–483)	**25[16 : 35]**	0.15[-12 : 14]	0.154
**18–29**	186 (94–311)	231 (121–397)	194 (115–351)	233 (113–409)	13[0.060 : 28]	-5.9[-21 : 12]	0.125	284 (158–430)	245 (126–453)	**50[21 : 86]**	1.8[-26 : 40]	0.051
**30–39**	205 (114–360)	213 (104–361)	223 (113–363)	243 (122–425)	6.3[-6.0 : 20]	23[4.6 : 45]	0.245	253 (160–432)	258 (130–458)	26[6.6 : 50]	44[7.0 : 94]	0.84
**40–49**	222 (120–405)	221 (112–385)	247 (135–410)	250 (126–410)	8.1[-3.6 : 21]	-4.4[-18 : 11]	0.729	284 (149–507)	278 (155–431)	26[7.5 : 47]	-13[-33 : 13]	0.911
**50–59**	242 (139–417)	260 (130–439)	262 (150–455)	297 (171–484)	11[-1.2 : 25]	6.4[-9.3 : 25]	0.629	343 (172–494)	312 (150–546)	21[2.2 : 44]	-4.4[-29 : 29]	0.758
**60–69**	276 (162–439)	305 (172–493)	278 (156–519)	346 (166–551)	2.6[-8.8 : 16]	0.68[-14 : 18]	0.661	288 (167–474)	284 (180–509)	6.2[-12 : 28]	-8.0[-32 : 24]	0.929

Smoking and age-stratified median concentrations

AGE GROUP	Non-smokers		Smokers		% Change in Concentration smokers vs non-smokers		
	Males Concentration (pg/ml)	Females Concentration (pg/ml)	Males Concentration (pg/ml)	Females Concentration (pg/ml)	Males change (%)	Females change (%)	Smoking × sex Interaction P
**All**	238 (129–408)	246 (130–431)	260 (136–440)	288 (138–465)	**11[3.7 : 20]**	**15[7.5 : 24]**	0.502
**18–29**	191 (99–332)	229 (122–402)	206 (111–348)	247 (113–415)	12[-3.2 : 31]	7.7[-7.1 : 25]	0.685
**30–39**	222 (121–369)	225 (114–374)	244 (127–405)	253 (126–513)	11[-6.1 : 32]	**22[3.7 : 45]**	0.43
**40–49**	243 (130–422)	238 (118–394)	244 (130–433)	282 (133–413)	4.8[-11 : 23]	16[-0.15 : 36]	0.357
**50–59**	249 (146–429)	265 (150–476)	314 (143–583)	297 (145–464)	**18[0.36 : 39]**	5.2[-11 : 24]	0.328
**60–69**	274 (155–474)	301 (165–510)	328 (208–523)	349 (186–575)	18[-0.050 : 40]	**24[5.4 : 46]**	0.697

Supplementary Table 48 VEGF-C (Vascular endothelial growth factor C, Flt-4L, LMPH1D, VRP, Uniprot:P49767)

Sex and age-stratified median concentrations

AGE GROUP	Biomarker/cohort information		Median Concentration			Age Group (% change in concentration)		
	N measured	Detection Range	Males	Females	Sex difference	Males	Females	Sex × age
		(% in range)	(pg/ml)	(pg/ml)	F relative to M (%)	change/10 years (%)	change/10 years (%)	Interaction P
**All**	9866	99.9	406 (302–529)	425 (316–553)	-0.95[-7.3 : 5.8]	**1.7[0.70 : 2.8]**	**3.0[1.9 : 4.0]**	0.1
**18–29**	1991	99.9	390 (287–505)	415 (303–541)	-9.9[-36 : 28]	-2.1[-12 : 8.5]	4.8[-5.2 : 16]	0.348
**30–39**	2009	99.9	396 (293–516)	399 (298–513)	63[-5.0 : 179]	8.9[-2.3 : 21]	-5.8[-15 : 5.0]	0.064
**40–49**	2010	99.9	410 (310–522)	419 (315–534)	-41[-70 : 17]	-3.4[-13 : 7.4]	9.1[-2.2 : 22]	0.116
**50–59**	1983	99.8	415 (307–549)	443 (329–586)	-31[-71 : 65]	1.5[-9.2 : 14]	9.5[-2.1 : 23]	0.349
**60–69**	1873	100	413 (314–547)	463 (353–599)	-79[-95 : -16]	**-23[-34 : -9.7]**	0.89[-14 : 19]	0.02

BMI and age-stratified median concentrations

AGE GROUP	Normal BMI		Overweight BMI					Obese BMI				
	Males	Females	Males	Females	Males	Females	Overweight × sex	Males	Females	Males	Females	Obese × sex
	Concentration (pg/ml)	Concentration (pg/ml)	Concentration (pg/ml)	Concentration (pg/ml)	%change	%change	Interaction P	Concentration (pg/ml)	Concentration (pg/ml)	%change	%change	Interaction P
**All**	395 (293–512)	413 (310–540)	413 (302–539)	441 (327–577)	0.70[-2.3 : 3.8]	2.2[-1.8 : 6.5]	0.055	435 (329–564)	449 (337–577)	**7.1[2.5 : 12]**	0.010[-7.1 : 7.7]	0.953
**18–29**	383 (275–510)	410 (298–536)	392 (291–494)	425 (317–559)	-0.76[-7.7 : 6.6]	10[-0.44 : 22]	0.148	434 (362–551)	453 (324–587)	13[-0.060 : 28]	19[-0.63 : 43]	0.942
**30–39**	385 (287–497)	379 (290–490)	392 (295–523)	406 (304–517)	1.3[-5.4 : 8.5]	5.0[-4.0 : 15]	0.233	444 (322–564)	437 (317–583)	13[2.8 : 24]	7.4[-8.9 : 27]	0.817
**40–49**	398 (309–496)	414 (316–525)	421 (304–551)	431 (317–577)	6.7[-0.26 : 14]	-8.3[-16 : 0.26]	0.354	440 (328–562)	437 (323–542)	11[1.3 : 22]	-18[-30 : -4.9]	0.423
**50–59**	412 (304–537)	425 (326–575)	414 (303–545)	466 (332–592)	-0.71[-7.4 : 6.4]	7.5[-2.2 : 18]	0.333	444 (330–606)	464 (355–597)	2.3[-7.4 : 13]	12[-6.0 : 34]	0.771
**60–69**	409 (319–549)	457 (348–589)	418 (309–558)	481 (360–625)	-2.1[-8.4 : 4.6]	4.4[-4.4 : 14]	0.06	392 (316–555)	464 (354–607)	-1.5[-11 : 9.4]	-1.1[-16 : 17]	0.375

Smoking and age-stratified median concentrations

AGE GROUP	Non-smokers		Smokers		% Change in Concentration smokers vs non-smokers		
	Males	Females	Males	Females	Males	Females	Smoking × sex
	Concentration (pg/ml)	Concentration (pg/ml)	Concentration (pg/ml)	Concentration (pg/ml)	change (%)	change (%)	Interaction P
**All**	405 (300–527)	423 (315–548)	421 (318–553)	444 (326–581)	**6.3[2.0 : 11]**	3.9[-0.21 : 8.2]	0.434
**18–29**	392 (285–513)	415 (301–538)	387 (288–474)	446 (319–565)	1.1[-7.2 : 10]	0.57[-7.6 : 9.5]	0.927
**30–39**	398 (295–521)	396 (295–507)	392 (289–500)	402 (299–519)	2.3[-6.9 : 12]	-2.0[-11 : 7.5]	0.525
**40–49**	410 (306–517)	417 (313–530)	473 (351–567)	445 (336–580)	**11[1.2 : 22]**	6.8[-2.4 : 17]	0.536
**50–59**	411 (303–537)	440 (330–581)	466 (340–584)	451 (331–608)	**11[1.4 : 23]**	6.2[-3.5 : 17]	0.486
**60–69**	412 (312–541)	460 (353–588)	447 (333–624)	494 (357–663)	6.6[-3.0 : 17]	6.3[-2.9 : 16]	0.974

Supplementary Table 49: VEGF-D (Vascular endothelial growth factor D, c-fos induced growth factor, Uniprot:O43915)

Sex and age-stratified median concentrations

AGE GROUP	Biomarker/cohort information		Median Concentration			Age Group (% change in concentration)		
	N measured	Detection Range	Males	Females	Sex difference	Males	Females	Sex × age
		(% in range)	(pg/ml)	(pg/ml)	F relative to M (%)	change/10 years (%)	change/10 years (%)	Interaction P
**All**	9866	100	1081 (772–1489)	1256 (912–1781)	**38[27 : 49]**	**5.5[4.2 : 6.8]**	**2.1[0.85 : 3.3]**	**1.57E-04**
**18–29**	1991	99.9	1004 (733–1336)	1225 (922–1889)	42[-5.8 : 114]	**20[6.3 : 35]**	16[3.1 : 30]	0.699
**30–39**	2009	99.9	1024 (740–1437)	1203 (879–1679)	**257[83 : 595]**	17[2.2 : 34]	-15[-26 : -2.9]	**0.001**
**40–49**	2010	100	1080 (755–1484)	1233 (906–1651)	10[-50 : 145]	4.9[-7.3 : 19]	6.2[-6.5 : 21]	0.887
**50–59**	1983	99.9	1160 (824–1526)	1292 (944–1832)	-41[-78 : 62]	-1.9[-14 : 12]	11[-2.5 : 27]	0.184
**60–69**	1873	100	1179 (833–1676)	1338 (929–1835)	84[-71 : 1064]	5.2[-14 : 29]	-2.7[-21 : 20]	0.596

BMI and age-stratified median concentrations

AGE GROUP	Normal BMI		Overweight BMI					Obese BMI				
	Males	Females	Males	Females	Males	Females	Overweight × sex	Males	Females	Males	Females	Obese × sex
	Concentration (pg/ml)	Concentration (pg/ml)	Concentration (pg/ml)	Concentration (pg/ml)	%change	%change	Interaction P	Concentration (pg/ml)	Concentration (pg/ml)	%change	%change	Interaction P
**All**	1095 (778–1515)	1322 (970–1885)	1081 (769–1468)	1221 (900–1708)	-3.2[-6.7 : 0.33]	-4.6[-9.2 : 0.14]	0.016	1058 (786–1492)	1120 (805–1487)	-2.4[-7.5 : 3.0]	-7.5[-15 : 1.2]	**3.31E-06**
**18–29**	1019 (737–1389)	1296 (978–1929)	1011 (736–1305)	1174 (873–1745)	-0.86[-8.9 : 7.9]	1.9[-9.4 : 14]	0.201	941 (711–1199)	1080 (744–1497)	-14[-26 : -0.98]	3.1[-17 : 28]	0.528
**30–39**	1095 (773–1469)	1280 (945–1845)	1005 (721–1362)	1165 (856–1536)	-4.5[-12 : 3.9]	-12[-21 : -1.5]	0.058	1007 (792–1587)	1082 (788–1372)	5.6[-6.1 : 19]	-11[-28 : 8.7]	**7.24E-04**
**40–49**	1101 (750–1507)	1262 (917–1676)	1061 (755–1467)	1252 (957–1720)	-1.3[-8.7 : 6.6]	4.0[-6.2 : 15]	0.638	1049 (777–1446)	1162 (836–1464)	1.7[-8.6 : 13]	-6.1[-21 : 12]	0.055
**50–59**	1177 (824–1548)	1334 (984–1958)	1159 (789–1488)	1288 (978–1769)	-6.2[-13 : 1.7]	-4.1[-14 : 6.9]	0.856	1129 (829–1596)	1182 (827–1624)	-3.2[-14 : 8.7]	-10[-27 : 9.8]	0.068
**60–69**	1277 (865–1836)	1461 (1049–1920)	1149 (835–1572)	1235 (876–1757)	-7.1[-15 : 1.3]	-5.9[-16 : 5.6]	0.452	1085 (703–1526)	1147 (849–1552)	-8.4[-20 : 5.0]	-8.6[-27 : 14]	0.136

Smoking and age-stratified median concentrations

AGE GROUP	Non-smokers		Smokers		% Change in Concentration smokers vs non-smokers		
	Males	Females	Males	Females	Males	Females	Smoking × sex
	Concentration (pg/ml)	Concentration (pg/ml)	Concentration (pg/ml)	Concentration (pg/ml)	change (%)	change (%)	Interaction P
**All**	1073 (771–1468)	1243 (908–1745)	1171 (796–1639)	1350 (1003–2047)	**8.3[3.0 : 14]**	**9.4[4.2 : 15]**	0.766
**18–29**	989 (738–1278)	1198 (916–1824)	1099 (706–1580)	1398 (1023–2215)	8.1[-2.3 : 20]	**14[3.6 : 26]**	0.428
**30–39**	1043 (748–1440)	1190 (866–1644)	1066 (803–1561)	1256 (960–1854)	**12[0.060 : 26]**	5.3[-6.0 : 18]	0.431
**40–49**	1072 (749–1464)	1227 (908–1631)	1165 (782–1665)	1321 (989–1784)	8.2[-3.1 : 21]	7.4[-3.2 : 19]	0.922
**50–59**	1128 (801–1497)	1283 (940–1811)	1277 (923–1688)	1383 (1043–2209)	**12[0.24 : 25]**	10[-1.2 : 24]	0.871
**60–69**	1177 (835–1667)	1338 (932–1797)	1271 (760–1884)	1387 (1020–1998)	-1.3[-13 : 12]	7.3[-4.7 : 21]	0.341
