# Supplementary material for: Lifestyle and demographic associations with 47 inflammatory and vascular stress biomarkers in 9876 blood donors
Source: Commun Med (Lond). 2024 Mar 16;4:50. doi: 10.1038/s43856-024-00474-2 (PMC10944541; doi:10.1038/s43856-024-00474-2)
Supplement: Supplementary file 3 — Description of Additional Supplementary Files [file 43856_2024_474_MOESM3_ESM.pdf]

Description of Additional Supplementary Files

File Name: Supplementary data 1
Description: *Available number of measurements for each marker and number and percentage below and above detection range. The count with questionnaire column is the number of measurements available with questionnaire answers available for analysis adjusted for smoking and BMI*

File Name: Supplementary Data 2
Description: *Estimated percentage change in concentration for each assay per storage year of sample. The analyses were adjusted for region and analysis date.*

File Name: Supplementary Data 3
Description: *The association (as % change pr donation) between biomarkers and number of donations within three years adjusted for age, BMI, smoking, region, sample storage time, and measurement date. P values were adjusted by multiplying with number of markers in the group and significant effects are in bold.*

File Name: Supplementary Data 4
Description: *The difference (%) between regions with the largest region (capital region) as reference adjusted for age, BMI, smoking, sample time of day, sample storage time, and measurement date. P values were adjusted by multiplying with number of markers in the group and significant effects are in bold.*

File Name: Supplementary Data 5
Description: *Mean and standard deviation for each assay stratified by age and sex.*

File Name: Supplementary Data 6
Description: *Females aged 50 or more compared to those below adjusted for BMI, age, smoking, sample storage time, region, and measurement date. P values were adjusted by multiplying with number of markers in the group and significant effects are in bold. Estimates are reported as % change .*

File Name: Supplementary Data 7
Description: *The association (% change pr 1 BMI unit increase) between biomarkers and BMI as a continuous variable in each age group stratified by sex adjusted for age, smoking, sample storage time, region, and measurement date. P values were adjusted by multiplying with number of markers in the group and significant associations are in bold.*

File Name: Supplementary Data 8
Description: contains source data for median scaled biomarker concentrations visualized in the fields in the heatmaps in Figure 2–4

File Name: Supplementary Data 9

Description: contains source data for the radar chart in Figure 5. Values are medians of scaled biomarker concentrations for the youngest and oldest age group and the 1st and 3rd quartiles used as min and max for axis limits.

File Name: Supplementary Data 10

Description: contains source data for the radar chart in Figure 6. Values are medians of scaled biomarker concentrations for the normal weight, overweight and obese groups and the 1st and 3rd quartiles used as min and max for axis limits.

File Name: Supplementary Data 11

Description: contains source data for the radar chart in Figure 7. Values are medians of scaled biomarker concentrations for the smokers and non-smokers and the 1st and 3rd quartiles used as min and max for axis limits.
