# Supplementary material for: Lifestyle and demographic associations with 47 inflammatory and vascular stress biomarkers in 9876 blood donors
Source: Commun Med (Lond). 2024 Mar 16;4:50. doi: 10.1038/s43856-024-00474-2 (PMC10944541; doi:10.1038/s43856-024-00474-2)
Supplement: Supplementary file 15 — Reporting summary [file 43856_2024_474_MOESM15_ESM.pdf]

nature portfolio

Corresponding author(s): Bertram Kjerulff

Last updated by author(s): Feb 5, 2024

Reporting Summary

Nature Portfolio wishes to improve the reproducibility of the work that we publish. This form provides structure for consistency and transparency in reporting. For further information on Nature Portfolio policies, see our Editorial Policies and the Editorial Policy Checklist.

Statistics

For all statistical analyses, confirm that the following items are present in the figure legend, table legend, main text, or Methods section.

n/a	Confirmed	
☐	☒	The exact sample size (*n*) for each experimental group/condition, given as a discrete number and unit of measurement
☐	☒	A statement on whether measurements were taken from distinct samples or whether the same sample was measured repeatedly
☐	☒	The statistical test(s) used AND whether they are one- or two-sided<br>*Only common tests should be described solely by name; describe more complex techniques in the Methods section.*
☐	☒	A description of all covariates tested
☐	☒	A description of any assumptions or corrections, such as tests of normality and adjustment for multiple comparisons
☐	☒	A full description of the statistical parameters including central tendency (e.g. means) or other basic estimates (e.g. regression coefficient) AND variation (e.g. standard deviation) or associated estimates of uncertainty (e.g. confidence intervals)
☐	☒	For null hypothesis testing, the test statistic (e.g. *F*, *t*, *r*) with confidence intervals, effect sizes, degrees of freedom and *P* value noted<br>*Give P values as exact values whenever suitable.*
☒	☐	For Bayesian analysis, information on the choice of priors and Markov chain Monte Carlo settings
☒	☐	For hierarchical and complex designs, identification of the appropriate level for tests and full reporting of outcomes
☐	☒	Estimates of effect sizes (e.g. Cohen's *d*, Pearson's *r*), indicating how they were calculated

Our web collection on statistics for biologists contains articles on many of the points above.

Software and code

Policy information about availability of computer code

Data collection	No software was used.
Data analysis	No software was used. Data analysis code is available online (https://doi.org/10.5281/zenodo.10217149).

For manuscripts utilizing custom algorithms or software that are central to the research but not yet described in published literature, software must be made available to editors and reviewers. We strongly encourage code deposition in a community repository (e.g. GitHub). See the Nature Portfolio guidelines for submitting code & software for further information.

Data

Policy information about availability of data

All manuscripts must include a data availability statement. This statement should provide the following information, where applicable:

- Accession codes, unique identifiers, or web links for publicly available datasets
- A description of any restrictions on data availability
- For clinical datasets or third party data, please ensure that the statement adheres to our policy

The DBDS is a platform for studies carried out by the Danish blood centres and collaborators. The study is managed by a steering committee who respond to enquiries regarding collaboration. The blood donors participate in the DBDS to increase the scope of their donation, i.e. to help produce valuable research for the benefit of future patients. Additional information can be found at our home page [http://www.dbds.dk]. We invite researchers to collaborate by contacting the steering committee [info@dbds.dk]. Data access requires that projects and applicants obtain permission from the Regional Committees on Health Research Ethics

and the Danish Data Protection Agency [http://www.datatilsynet.dk]. Data analysis code is available online (https://doi.org/10.5281/zenodo.10217149). Source data for figures in the main manuscript have been uploaded to Communications Medicine as excel files.

Human research participants

Policy information about studies involving human research participants and Sex and Gender in Research.

Reporting on sex and gender	The term sex (biological attribute) has been used throughout this manuscript.
Population characteristics	The study population comprised 9,876 blood donors participating in the Danish Blood Donor Study (DBDS). Participants for this study, DBDS 10K Inflammatory Biomarker Cohort, were selected among all DBDS participants who had inclusion samples stored in the biobank. Among the available samples, we selected selected samples from participants ensuring equal numbers of participants in sex- and age-stratified groups. The median age was 45 years and we stratified into five equal-sized age groups.
Recruitment	In this study, participants are blood donors recruited at the bleeding facilities in Denmark while they donate blood. Because blood donors must comply with strict health criteria to donate, they do not perfectly represent the background population. They are generally healthier, have a higher income and education level than the background population, and the prevalence of donors is higher in urban areas. Additionally, a larger proportion has at least one Danish parent, and for males there is an association with cohabitation with a female, as previously described in published article (PMID: 28182624). Because of the selection bias of healthy donors, we have taken into account for the 'healthy-donor-effect' which is equivalent to the well-described 'healthy-worker-effect'.
Ethics oversight	Oral and written informed consent was obtained from all study participants. The DBDS was approved by the Danish Data Protection Agency (P-2019-99) and the Committees on Health Research Ethics in the Central Denmark Region (1-10-72-95-13) and the Zealand Region (SJ-740).

Note that full information on the approval of the study protocol must also be provided in the manuscript.

Field-specific reporting

Please select the one below that is the best fit for your research. If you are not sure, read the appropriate sections before making your selection.

☒ Life sciences ☐ Behavioural & social sciences ☐ Ecological, evolutionary & environmental sciences

For a reference copy of the document with all sections, see nature.com/documents/nr-reporting-summary-flat.pdf

Life sciences study design

All studies must disclose on these points even when the disclosure is negative.

Sample size	Participants for the DBDS 10K Inflammatory Biomarker Cohort were selected among 128,017 available inclusion samples from DBDS participants. The first randomly chosen 982 samples served as a pilot study, which were followed by an additional 9,000 samples. Among the available samples, 10,277 were picked, ensuring equal numbers of participants in sex- and age-stratified groups.
Data exclusions	We excluded 1,383 samples due to quality concerns (freeze-thaw cycles, low sample volume, caps that had become loose in storage), missing samples, or withdrawal of consent prior to the final data analysis. A total of 9,876 samples were included in the final cohort.
Replication	The samples were analyzed using the Meso Scale Discovery (MSD) V-PLEX Human Biomarker 54-plex kit according to the manufacturer's instructions. Quality control measures were implemented to ensure data quality and reproducibility. We found that several of the TH17 panel 1 biomarkers had quality control issues that could not be resolved despite numerous efforts from the manufacturer MSD and the involved laboratories. The TH17 panel measurements were therefore discontinued and excluded from further analysis.
Randomization	The randomization process for sample selection is detailed in the 'Sample Size' section.
Blinding	Inclusion into the DBDS occurred at donation facilities as time permitted and was not blinded. Participants were randomly selected for the DBDS 10K Inflammatory Biomarker Cohort using an automated algorithm.

Reporting for specific materials, systems and methods

We require information from authors about some types of materials, experimental systems and methods used in many studies. Here, indicate whether each material, system or method listed is relevant to your study. If you are not sure if a list item applies to your research, read the appropriate section before selecting a response.

Materials & experimental systems

n/a	Involved in the study
☒	☐ Antibodies
☒	☐ Eukaryotic cell lines
☒	☐ Palaeontology and archaeology
☒	☐ Animals and other organisms
☒	☐ Clinical data
☒	☐ Dual use research of concern

Methods

n/a	Involved in the study
☒	☐ ChIP-seq
☒	☐ Flow cytometry
☒	☐ MRI-based neuroimaging
